# Versatile control of the submolecular motion of di(acylamino)pyridine-based [2]rotaxanes†
†Electronic supplementary information (ESI) available: Experimental procedures, spectroscopic and mass spectrometry data for all new compounds, electrochemical studies, and full crystallographic details of **1c** and **2b**. CCDC 1051908 and 1051909. For ESI and crystallographic data in CIF or other electronic format see DOI: 10.1039/c5sc00790a



**DOI:** 10.1039/c5sc00790a

**Published:** 2015-03-18

**Authors:** Alberto Martinez-Cuezva, Aurelia Pastor, Giacomo Cioncoloni, Raul-Angel Orenes, Mateo Alajarin, Mark D. Symes, Jose Berna

**Affiliations:** a Departamento de Química Orgánica , Facultad de Química , Regional Campus of International Excellence “Campus Mare Nostrum” , Universidad de Murcia , E-30100 , Murcia , Spain . Email: ppberna@um.es; b WestCHEM , School of Chemistry , University of Glasgow , University Avenue , Glasgow G12 8QQ , UK; c SAI , Universidad de Murcia , E-30100 , Murcia , Spain

## Abstract

A cyclic network of chemical reactions has been conceived for exchanging the dynamic behaviour of di(acylamino)pyridine-based rotaxanes and surrogates. X-ray diffraction studies revealed the intercomponent interactions in these interlocked compounds and were consistent with those found in solution by dynamic NMR experiments. This particular binding site was incorporated into a molecular shuttle enabled for accessing two states with an outstanding positional discrimination through chemical manipulation. Furthermore, the ability of the di(acylamino)pyridine domain to associate with external binders with a complementary array of HB donor and acceptor sites was exploited for the advance of an unprecedented electrochemical switch operating through a reversible anion radical recognition process.

## Abstract 2

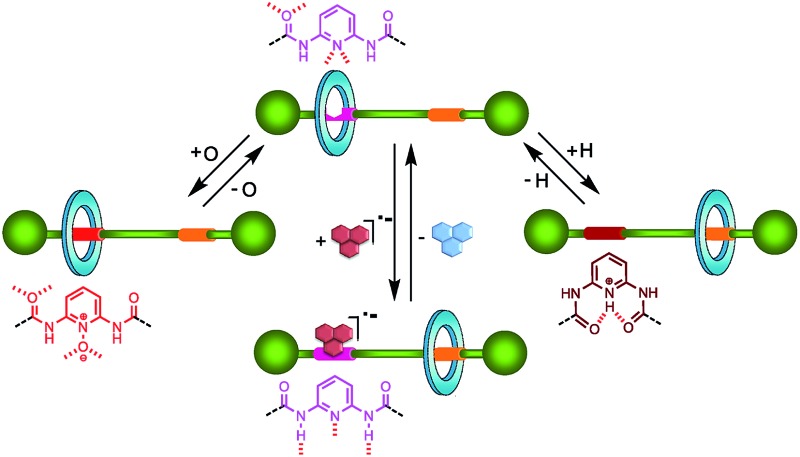
Di(acylamino)pyridine motifs enable the building of switchable interlocked systems in which their dynamics can be exchanged between different states.

## Introduction

The development of synthetic structures that mimic dynamic aspects of biological systems is a major focus nowadays.^1^ Essential functions inside living organisms are achieved by natural molecular motors, which perform the herculean task of controlling the incessant Brownian motion.^2^ Restricted motion of entwined mobile parts constitutes one of the most intriguing features of these functional natural compounds.^3^ An increasing number of interdisciplinary research teams have established many methods, not only in the assembly of artificial interlocked systems, but also in the minimalist mirroring of vital processes, by means of the precise control of the relative motion of their components. In this sense, the role of non-covalent interactions, in particular that of hydrogen bonding, is essential to manipulate the relative position and directionality of movement within these artificial intertwined structures.^2c,4^

We recently added an unprecedented motion-triggering mechanism to the interlocked hydrogen-bonded machinery toolbox.^5^ Thus, the di(acylamino)pyridine (DAP) function arises as an effective template for the formation of hydrogen-bonded tetralactam rotaxanes through five-component clipping reactions. Nevertheless, the added value of DAP-based rotaxanes is the presence of a well-defined donor–acceptor–donor (D–A–D) hydrogen bond pattern. The molecular recognition^6,7^ between the di(acylamino)pyridine subunit and complementary external binders restricts the amplitude of the ring motion in degenerate molecular shuttles.^5^ The original translation can be restored through a competitive recognition event.

At this point, we were inspired not only by the supramolecular ability of the di(acylamino)pyridine platform but by its rich molecular chemistry. We considered that the rearrangement of the protonated DAP subunit into a folded conformation could dramatically decrease its affinity to the macrocycle. Conversely, the corresponding *N*-oxide would increase the attraction between the oxidized station and the ring. Furthermore, electrochemical reduction of poor binders into the corresponding radical anions would noticeably increase the association ability by the DAP-station. Thus, our interest was redirected to the construction of versatile chemical and electrochemically-driven molecular shuttles^8^ by exploiting the multiple properties of the DAP domain.

Herein, we report the synthesis of three-state switchable systems^9^ by using two rather simple and reversible chemical transformations: protonation and oxidation of the DAP function. The effects of both processes on the pirouetting or shuttling motions of single-binding site rotaxanes or molecular shuttles containing a second station of different nature have been evaluated. Moreover, we have combined a molecular recognition event with an electrochemical input to control the motion of the macrocycle in DAP-based molecular shuttles.

## Results and discussion

### Switching of the rotational motion of the macrocycle in di(acylamino)pyridine-based [2]rotaxanes

**Scheme 1:**
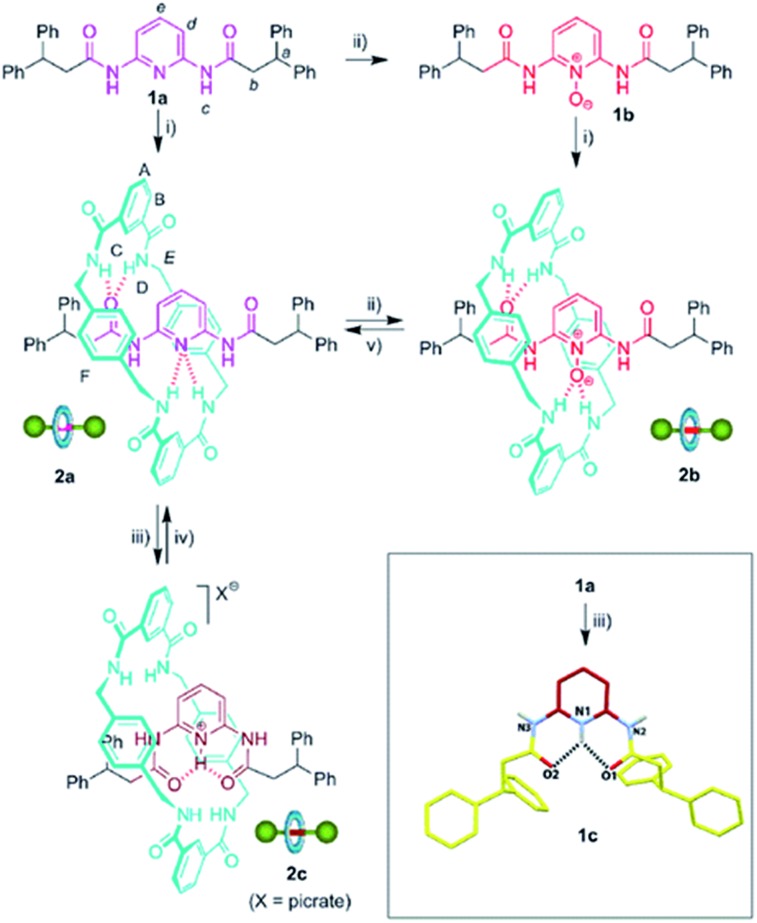
Synthesis and interconversion of di(acylamino)pyridine-based [2]rotaxanes and surrogates. Reagent and conditions: (i) isophthaloyl dichloride, *p*-xylylenediamine, Et_3_N, CHCl_3_, **2a**, 33% (ref. 5); **2b**, 14%; (ii) *m*-CPBA, CHCl_3_, **1b**, 96%; **2b**, 98%; (iii) picric acid, CHCl_3_, **1c**, quant.; **2c**, quant.; (iv) Amberlyst® A-21, quant.; (v) MoO_2_Cl_2_(DMF)_2_ (10%), diphenylphosphino-polystyrene, PhCH_3_–CH_3_CN, 96%. Full experimental procedures can be found in the ESI.† Inset: molecular structure of **1c** (CCDC 1051908)† in the solid state. For clarity, the picrate anion and a methanol co-solvate molecule have been omitted. Carbon atoms are shown in yellow, except those of the pyridine ring which are in maroon; oxygen atoms are depicted in red, nitrogen atoms in blue, and selected hydrogen atoms in white. Hydrogen-bond lengths and angles are given in the ESI.†

The design of controllable interlocked molecular shuttles requires the estimation of the ring affinity towards the embedded binding sites. In benzylic amide [2]rotaxanes, this affinity is closely related to the energy barrier for the rotation of the macrocyclic tetralactam around a complementary H-bond acceptor, such as an acyclic 1,2-dicarboxamide, placed in the thread.^10^ Being aware that the di(acylamino)pyridine function is a reasonable good template for benzylic amide rings, we explored the rotational energy barrier of DAP-based [2]rotaxanes and surrogates in their protonated/oxidized forms (Scheme 1). We have previously reported the synthesis of the corresponding thread **1a** and the rotaxane **2a**, obtained from the former through a five-component clipping reaction with *p*-xylylenediamine and isophthalic acid dichloride.^5^ The smooth oxidation of **2a** with *m*-CPBA efficiently afforded the corresponding *N*-oxide **2b**. The treatment of **2a** with picric acid quantitatively led to the protonated interlocked compound **2c**. The recovering of the initial rotaxane **2a** from both chemically modified states was accomplished by using polymer-supported reagents in order to enhance the feasibility of an iterative exchange between the three interlocked forms. Thus, the deprotonation of **2c** was straightforward by using Amberlyst® A-21. Unsurprisingly, the steric hindrance of the active groups of diphenylphosphino-polystyrene precluded the reduction of the interlocked *N*-oxide **2b**.^11^ Nevertheless this transformation could be achieved by using the same polymer in the presence of 0.1 equiv. of MoO_2_Cl_2_(DMF)_2_ as catalyst.^12^ It is worth noting that this protocol notably improves those described in the literature for the reduction of carboxamidopyridine (amido-substituted pyridine) *N*-oxides.^13^ The routes delineated in Scheme 1 allow the exchange between three rotaxanes **2a–c** differing in the chemical state of their pyridine N atom.

It should be pointed out that [2]rotaxane **2b** is also available through a five-component clipping reaction from the corresponding *N*-oxide of the thread, **1b** (Scheme 1). Note that [2]rotaxane **2a** is obtained from **1a** in a higher yield^5^ (33%) compared to that of **2b** from the corresponding thread, **1b** (14%). Taking into account the better H-bond acceptor ability of the pyridine *N*-oxide function (**1b**) compared to the less basic pyridine ring (**1a**),^14^ this lower yield of the interlocked compound seems to indicate that the geometrical organization of the H-bond acceptors of **2b** are moving away from the ideal one for an efficient templation, highlighting the optimal spatial arrangement in the amide macrocyclization chemistry developed by Leigh and co-workers.^15^ On the other hand, the protonation of **1a** promotes a 180° rotational isomerization of the amide groups^16*a*^ of **1c** for adopting an alternative double s-*cis* conformation which is stabilized by a bifurcated hydrogen bond (see inset of Scheme 1 for its molecular structure in the solid state). This thread conformation in the rotaxane **2c** would be responsible for the striking change in the ring dynamics of **2a** following its protonation (see below).

We next focused our attention on estimating the energy barrier of rotation of the tetralactam ring in rotaxanes **2a–c**. At a given temperature, if the intercomponent interactions are relative weak a fast exchange between the methylene protons of the macrocycle should be observed on the NMR time scale. Obviously, the rate of this exchange process would decrease on lowering the temperature, resulting in the splitting of the broad singlet corresponding to these protons in the ^1^H-NMR spectrum.^17^ This is why we next carried out low-temperature ^1^H NMR experiments with deuterated dichloromethane solutions of **2a–c** (see Fig. S1–S3†).

**Fig. 1:**
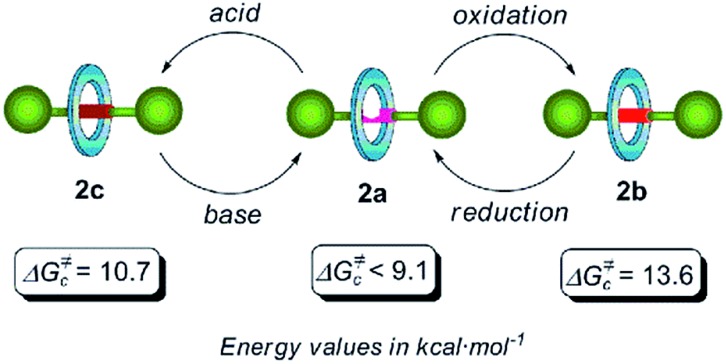
Chemical switching of the three rotational dynamic states in di(acylamino)pyridine-based [2]rotaxanes and surrogates, and their rotational energy barriers.

Concerning rotaxane **2a**, bearing a di(acylamino)pyridine station, we could only estimate a maximum limit of 9.1 kcal mol^–1^ since the expected splitting was not observed in the temperature range studied here (300–200 K) (Fig. S1†). By carrying out a similar NMR study with the rotaxane **2b** we calculated an energy barrier of 13.6 kcal mol^–1^ (*T*_c_ = 298 K) for the pirouetting of the macrocycle (Fig. S2†). The presence of the *N*-oxide function in this rotaxane notably improves the ability as a hydrogen bond acceptor of the binding subunit, when compared with the pyridine ring in **2a**, thus causing a noticeable reduction of the spinning rate of the tetralactam macrocycle. On the other hand, the VT-NMR analysis of the picrate **2c** disclosed that the energy barrier of the rotational motion of the amide benzylic macrocycle is 10.7 kcal mol^–1^ (*T*_c_ = 231 K) (Fig. S3†). As we hypothesized, the protonation of the nitrogen atom of the pyridine ring seems to promote a conformational rearrangement^16*a*^ of the axis **1c** resulting into a clear steric interference of the ring rotation.^10c,18^ These three switchable dynamic states are summarized in Fig. 1. Both oxidation and protonation processes slow down the spinning of the macrocycle, allowing this particular template, based on the di(acylamino)pyridine functionality, to be employed for constructing an exchangeable three dynamic states system (Fig. 1).

### Molecular structure of the hydrogen-bonded rotaxane **2b** in the solid state

**Fig. 2:**
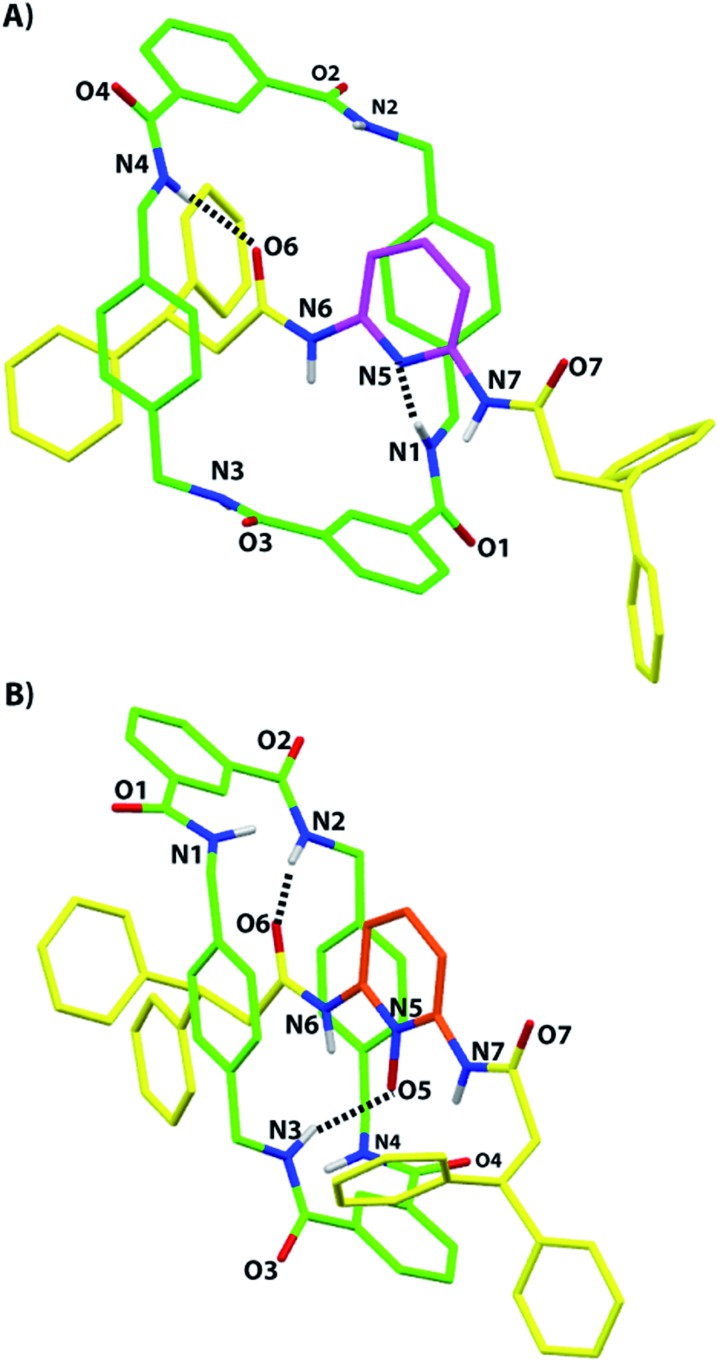
X-ray crystal structures of (A) the 2,6-di(acylamino)pyridine [2]rotaxane **2a**, taken from ref. 5, and (B) its *N*-oxide **2b** (CCDC 1051909).† For clarity, the carbon atoms of the macrocycle are shown in green, the carbon atoms of the thread in yellow, and the carbon atoms of the pyridine ring of **2a** in magenta and the same of **2b** in pale red; oxygen atoms are depicted in red, nitrogen atoms in blue, and selected hydrogen atoms in white. Intramolecular hydrogen-bond lengths [Å] (and angles [°]) for **2a**: N1–H01–N5 2.20 (170.8); N4–H04–O6 2.03 (168.4) and for **2b**: N2–H02–O6 2.07 (170.6); N3–H03–O5 1.99 (160.9).

Suitable monocrystals for X-ray diffraction measurements were obtained by slow cooling of a solution of the *N*-oxide **2b** in acetonitrile. As with the previously reported structure of **2a**,^5^ the resulting structure of **2b** displays the presence of two hydrogen bonds between two NH groups of the macrocycle and two of the three available acceptors of the thread: one of the two oxygen atoms of the diacylamino functionality (2.07 Å, 171°) and the *N*-oxide oxygen atom (1.99 Å, 161°). It is interesting to note the similarity of the overall conformations of **2a** and **2b** in the solid state (Fig. 2). Unfortunately, we were unable to obtain suitable monocrystals of **2c** from a plethora of solvent combinations and by using a variety of interchanged anions.

### Chemically driven mechanical motion in di(acylamino)pyridine-based molecular shuttles

**Scheme 2:**
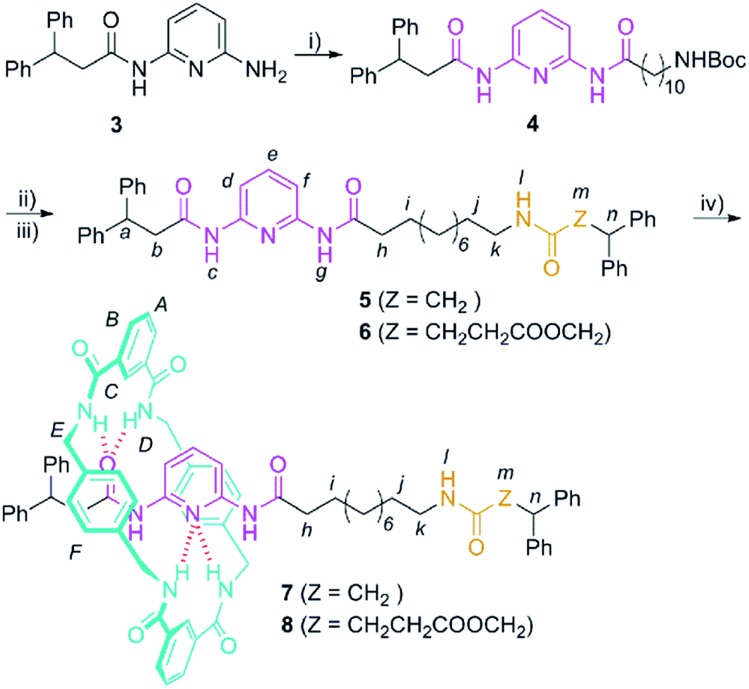
Synthesis of the DAP-based threads **5** and **6** and the corresponding [2]rotaxanes **7** and **8**. Reagents and conditions: (i) 11-(*tert*-butoxycarbonylamino)undecanoic acid (**9**), DMAP, EDCI, CH_2_Cl_2_, 70%; (ii) TFA, CHCl_3_; (iii) 3,3-diphenylpropanoic acid (**10**) or 2,2-diphenylethyl monosuccinate (**11**), DMAP, EDCI, CH_2_Cl_2_, **5** (39%); **6** (38%) (*yields over two steps*); (iv) isophthaloyl dichloride, *p*-xylylenediamine, Et_3_N, CHCl_3_; **7** (Z = CH_2_), 28%; **8** (Z = CH_2_CH_2_CO_2_CH_2_), 23%.

At this point, we envisaged that the efficiency of the chemical interconversions described above for DAP-based rotaxanes could be used for the building of chemically-driven molecular shuttles. Thus, we designed the two-station [2]rotaxanes **7** and **8** (Scheme 2). Both rotaxanes consist of a stoppered linear component, containing one DAP unit and one amide (**7**) or succinic amide ester (**8**) binding site, threaded through a tetrabenzylic amide macrocycle.

The synthesis of the interlocked compounds **7** and **8** was accomplished by means of well-known methods (Scheme 2). Hence, the coupling reaction of the monoacylamine^5^ **3** with 11-(*tert*-butoxycarbonylamino)undecanoic acid^19^ (**9**) provided the *N*-Boc protected amine **4**. The carbamate deprotection of **4** led to the corresponding tethered amine, which was further reacted with 3,3-diphenylpropanoic acid (**10**) or 2,2-diphenylethyl monosuccinate^20^ (**11**) to provide the corresponding threads **5** and **6** having the two binding sites. Finally, the two-station [2]rotaxanes **7** and **8** were obtained in 28 and 23% yield, respectively, through a five-component clipping reaction involving *p*-xylylenediamine and isophthaloyl chloride in the presence of triethylamine (Scheme 2).

Next, we investigated the levels of occupancy of the di(acylamino)pyridine site by the tetraamide ring in rotaxanes **7** and **8** by using well-established methodologies.^11f,20^ The percentage occupancy can be estimated by comparing the upfield shift experienced by the proton at the 4-position of the pyridine ring (H_e_ in structures **7** and **8**, Scheme 2) following rotaxane formation, with the same shift occurring in the synthesis of rotaxane **2a** (Table S1†). This later shift is associated with a complete occupation of the DAP binding station. This analysis revealed that the occupation of that station in **7** (68%) is notably higher than in **8** (28%). Most probably, this difference is due to the participation of the second carbonyl acceptor of the succinic ester function of **8** in the binding event, which notably decreases the occupation of the competing DAP-based station.

**Scheme 3:**
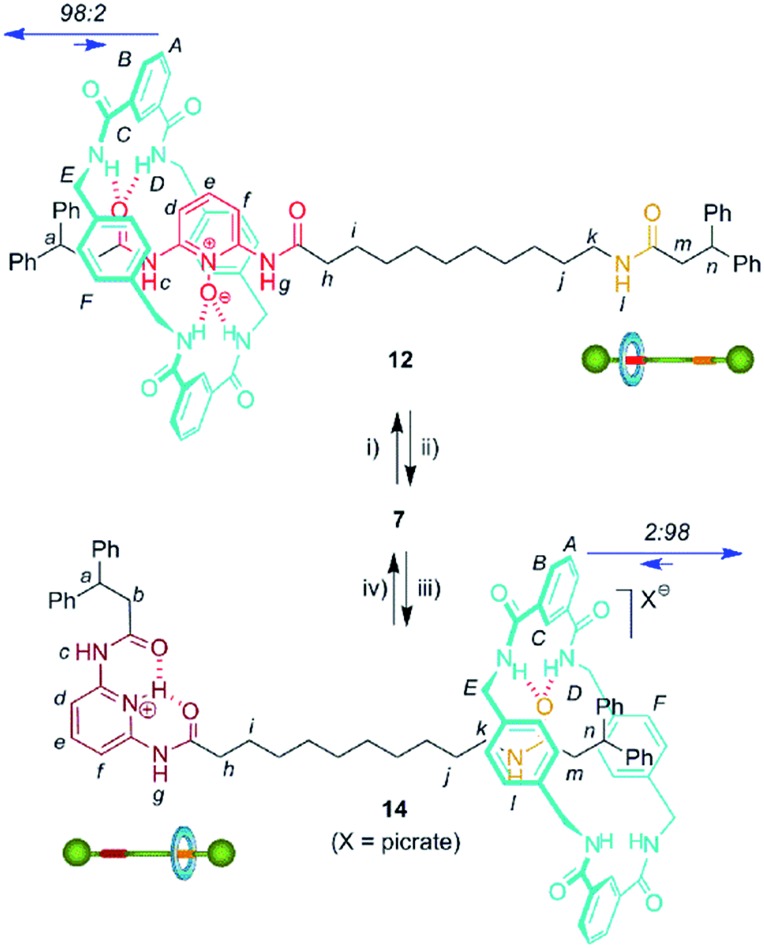
Interconversion of DAP-based [2]rotaxanes **7**, **12** and **14**. Reagents and conditions: (i) *m*-CPBA, CHCl_3_, 99%; (ii) MoO_2_Cl_2_(DMF)_2_ (10%), diphenylphosphino-polystyrene, PhCH_3_–CH_3_CN, 96%; (iii) picric acid, CHCl_3_, quant.; (iv) Amberlyst® A-21, quant. Full experimental procedures can be found in the ESI.†

**Scheme 4:**
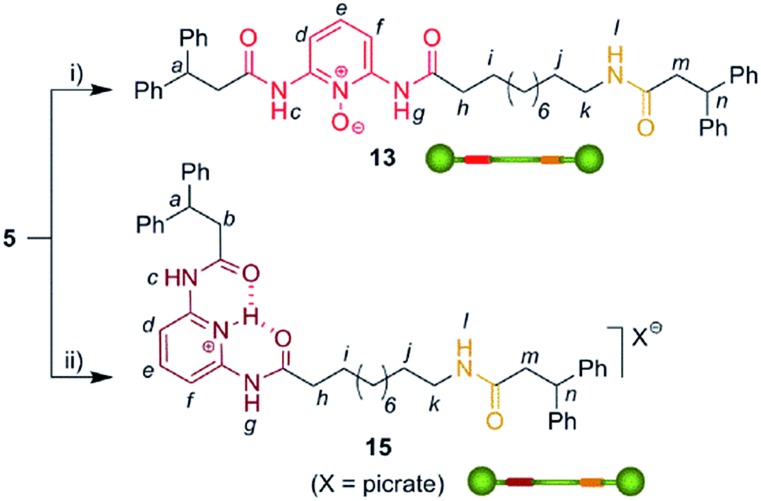
Synthesis of the threads **13** and **15**. Reagents and conditions: (i) *m*-CPBA, CHCl_3_, 96%; (ii) picric acid, CHCl_3_, quant. (see ESI†).

We then selected rotaxane **7** for exploring the effect of the protonation and oxidation of the di(acylamino)pyridine station on the ring-shuttling motion. The treatment of **7** with *m*-CPBA quantitatively led to the interlocked *N*-oxide **12** (reaction conditions (i), Scheme 3). For comparison, the corresponding non-interlocked *N*-oxide (**13**) was prepared from **5** by using similar reaction conditions (Scheme 4). The protonation of the DAP unit of the entwined **7** with picric acid led to **14** (reaction conditions (iii), Scheme 3). Likewise the protonated thread **15** was obtained from the corresponding neutral compound **5** (see ESI†).

**Fig. 3:**
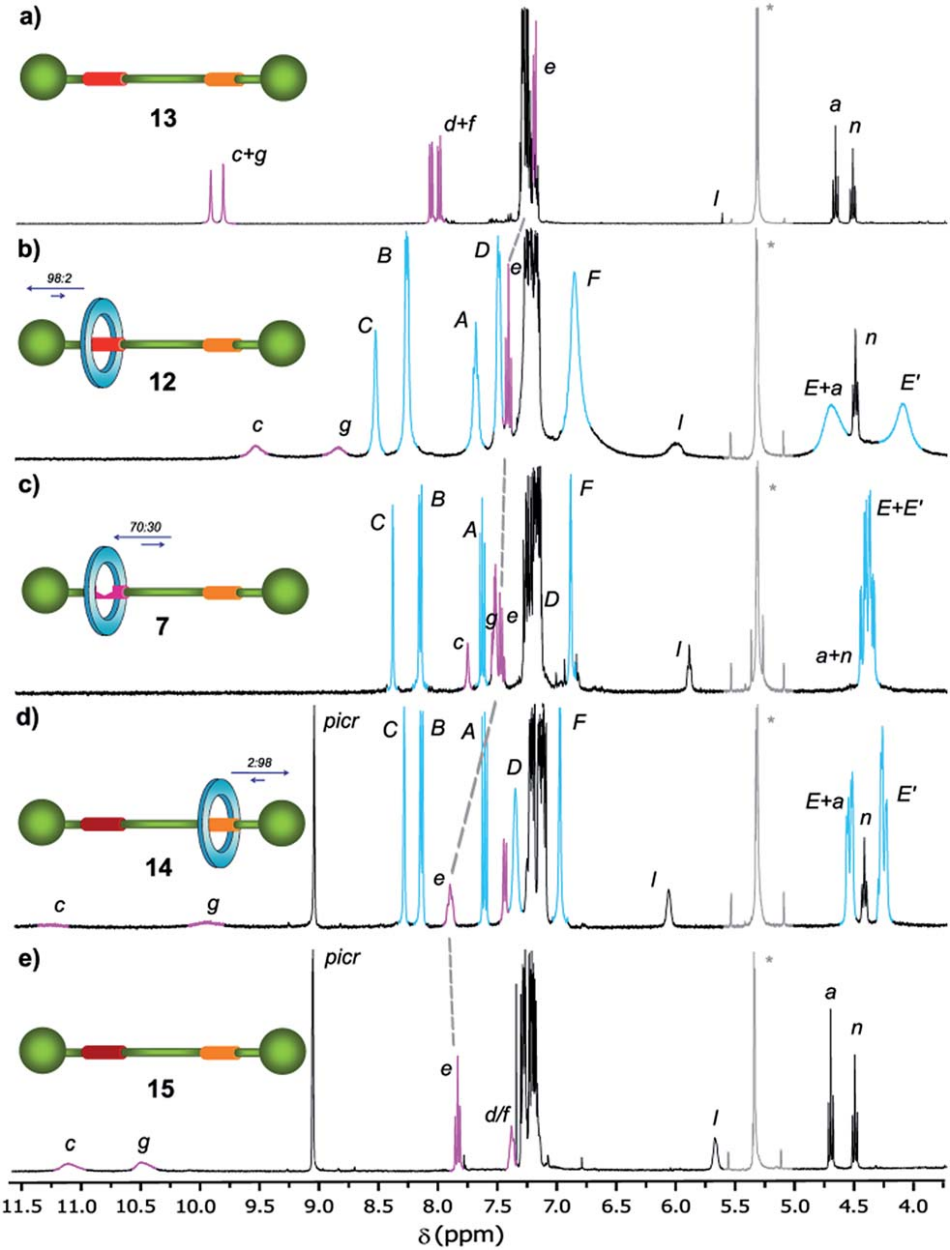
^1^H NMR spectra (400 MHz, CD_2_Cl_2_, 298 K) of (a) *N*-oxide thread **13**, (b) *N*-oxide rotaxane **12**, (c) DAP-based rotaxane **7**, (d) protonated DAP-based rotaxane **14** and (e) protonated DAP-based thread **15**. The residual solvent (dichloromethane) signal is indicated by an asterisk. The assignments correspond to the lettering shown in Scheme 2.

In order to analyse the effect of the oxidation of shuttle **7** on its ring distribution we compared the ^1^H NMR spectra of the molecular shuttle **12** and its corresponding thread **13**. The difference in the chemical shift of the proton H_e_ (Fig. 3) between **12** and **13** [Δ*δ*(H_e_) = +0.13 ppm] is the same as that between **2b** and **1b** (Table S1†). This result suggests that the occupation of the di(acylamino)pyridine station is complete in the shuttle **12**. As we expected, the benzylic amide ring of **12** strongly interacts with the *N*-oxide DAP binding site enhancing notably the modest positional integrity of the ring in the non-oxidized precursor **7**.

On the other hand, the chemical shift of H_e_ in the protonated rotaxane **14** and its thread **15** (see Table S1†) are nearly identical whereas the shift of H_e_ in the simpler pyridinium picrates **2c** and **1c** are clearly different [Δ*δ*(H_e_) = –0.22 ppm]. This divergence reveals that the protonation of the DAP unit of **7** precludes the siting of the ring on this station of the resulting **14** due to its conformational rearrangement driven by the formation of intramolecular hydrogen bonds^16*a*^ between the proton of the pyridinium moiety and the carbonyl amido groups (Scheme 3). The change in the conformation of shuttle **14**, and consequently the translocation of the macrocycle,^16b,c^ is supported by its ^1^H,^1^H-NOESY spectrum, which reveals the lack of crosspeaks relating the protons of the macrocycle and those of the di(acylamino)pyridine station (Fig. S11†). Indeed, the spectrum shows intense crosspeaks between the F and C hydrogens of the ring and those close to the amido binding site (Fig. S12†).

We were able to establish the exchange between rotaxanes **7**, **12** and **14** in an efficient manner. Thus, the treatment of the protonated shuttle **14** with a commercially available basic resin (reaction conditions (iv), Scheme 3) quantitatively afforded **7**. In this process the DAP domain recovers its native conformation, causing the ring to shuttle to occupy predominantly the DAP binding site. Finally, in order to complete a full cycle of chemical transformations, the deoxygenation of **12** with the dioxomolybdenum(vi) catalyst in the presence of diphenylphosphino-polystyrene (reaction conditions (ii), Scheme 3) efficiently regenerated rotaxane **7** and consequently, the original ring distribution of the interlocked system.

### Controlling the translational motion by molecular recognition with a neutral guest

**Fig. 4:**
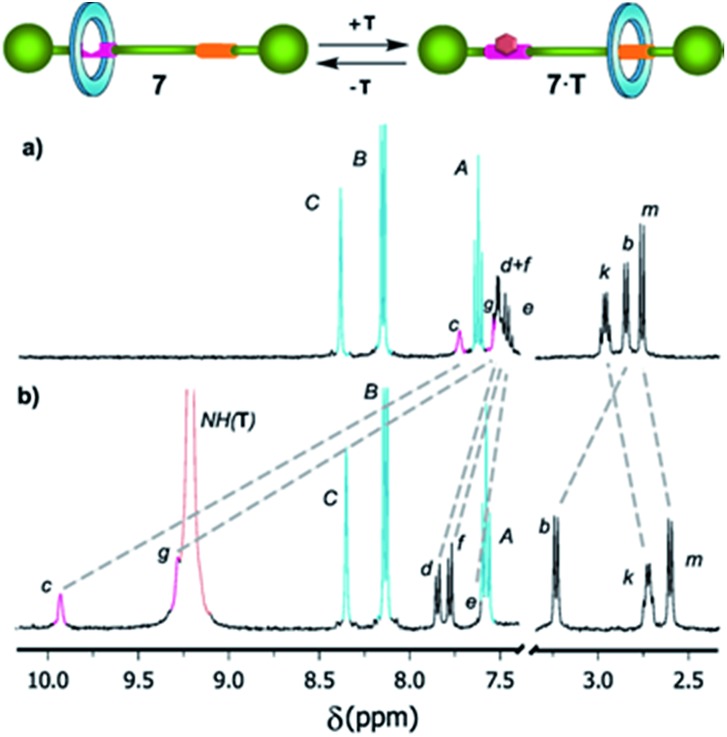
Selected regions of the ^1^H NMR spectra (400 MHz, CD_2_Cl_2_, 298 K) of (a) shuttle **7** and (b) after adding an excess (20 equiv.) of *N*-hexylthymine (**T**) (see Scheme 2 for signal assignments).

We have recently shown that the complexation with external binders containing a complementary array of H-bond donor and acceptor sites dynamically blocks the binding sites of DAP-based rotaxanes and, consequently, alters the amplitude of the ring motion.^5^ In this context, we wondered if the level of occupancy of the DAP-based shuttle **7** could be modified by complexation with *N*-hexylthymine. For that purpose, we performed titration experiments of rotaxane **7** using *N*-hexylthymine (**T**) as a guest, and then we calculated the corresponding association constant *K*_assoc_. For comparison, we also determined *K*_assoc_ for the complexation of its thread **5** with the same binder, **T**. For that purpose, we monitored the changes in the ^1^H-NMR spectra of thread **5** and rotaxane **7** by progressive addition of *N*-hexylthymine (Fig. S4 and S5†). We found *K*_assoc_ = 615 M^–1^ for the formation of the 1 : 1 complex **5**·**T**. As expected, the association constant for the complex **7**·**T** (*K*_assoc_ = 286 M^–1^) was notably lower due to the competition between the macrocycle and the guest for the occupation of the DAP station. The complexation between the thymine surrogate and the DAP binding site of the rotaxane drives the displacement of the macrocycle to the amide station. This fact was also supported by the observed shifting of the signal corresponding to the H_e_ proton of **7** (initially at 7.48 ppm, Fig. 4) to lower field by the progressive addition of the guest. The initial state could be easily restored by using a competing binder^21^ which associates with the thymine derivative more efficiently than the DAP station of **7**. Alternatively, the initial state could be also recovered by chromatography.

### Electrochemically driven mechanical motion in di(acylamino)pyridine-based molecular shuttles

**Fig. 5:**
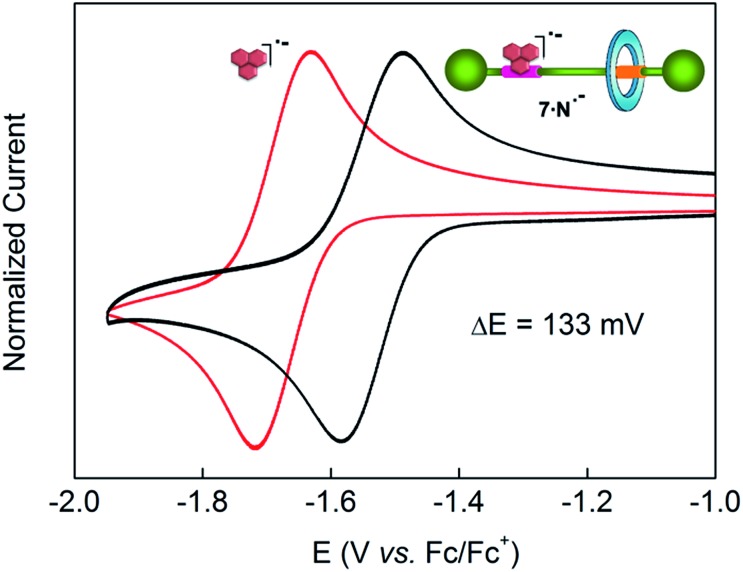
Cyclic voltammetry response of naphthalimide (**N**) *vs.* ferrocene (in CH_2_Cl_2_ with 1 M TBAPF_6_ as supporting electrolyte at 23 °C). The red trace corresponds to the free naphthalimide in solution (2.5 mM) (*E*_1/2_ = –1.67 V), the black trace shows the voltammogram at values of maximal shift, Δ*E*_1/2_ (also quoted on the graph), in the presence of an excess of rotaxane **7**. Scan rate: 100 mV s^–1^. Working electrode: glassy carbon. The *y*-axis currents have been normalized for ease of comparison.

Control of the strength of the hydrogen bonding network built between DAP arrays and suitable guests, such as flavin derivatives^22^ or naphthalimide,^23^ can be achieved using electrochemical methods. Such guests undergo reversible one-electron reduction in aprotic media to form radical anions. With this in mind we decided to explore how this redox process would affect the translational ring motion in the DAP-based rotaxane **7** using naphthalimide (**N**) as the guest. First, we carried out a standard ^1^H NMR titration with the fully oxidized form of **N** to obtain a *K*_assoc_ = 73 M^–1^ for the complex **7**·**N**. This rather low value is fully consistent with the reported ones for similar complexes.^23b,c^ Electrochemical generation of the corresponding radical anion of naphthalimide (**N˙^–^**) should dramatically change the association constant as it transforms a rather poor binder into a good one. In order to quantify the binding of **N˙^–^** to the DAP binding site of **7**, we investigated the change in the half-wave reduction potential (*E*_1/2_) of naphthalimide upon addition of aliquots of the interlocked host in CH_2_Cl_2_. Addition of **7** resulted in a substantial shift of *E*_1/2_ to less negative values (Fig. 5), indicating the significant stabilization of the radical anion through hydrogen bonding with the DAP station of the rotaxane.^24^ All the voltammograms were fully reversible, suggesting that there are no accompanying proton transfers during reduction and re-oxidation of the naphthalimide.

In this experiment, the shift in the half-potential is directly convertible into the association constants using the following equation:^25^ *K*_a_(red)/*K*_a_(ox) = exp[(*nF*/*RT*)(*E*_1/2_(bound) – *E*_1/2_(unbound))] = exp[(*nF*/*RT*)(Δ*E*_1/2_)]where Δ*E*_1/2_ is the maximum shift observed for the 1,8-naphthalimide redox wave (*x*-axis of Fig. 5), *n* = number of electrons in the wave (= 1, as determined by bulk electrolysis of naphthalimide at –1.9 V *vs.* ferrocene/ferrocenium – see ESI†), *F* is the Faraday constant, *R* is the molar gas constant and *T* is the temperature. This maximum value of Δ*E*_1/2_ was reached after the addition of around 6 equiv. of **8** (see Fig. S7†), and corresponds to 133 ± 4 mV, a value agreeing well with that obtained by Smith and co-workers for the maximum shift in *E*_1/2_ of 1,8-naphthalimide upon binding to 2,6-dipropylamidopyridine (128 mV).^23*a*^ Consequently, we determined the *K*_a_(red)/*K*_a_(ox) ratio of our system to be 178, thus resulting in an association constant of ∼13 000 M^–1^ for the complex **7**·**N˙^–^**. This high value seems to point out that the mechanical perturbation at the DAP site caused by the threaded tetralactam ring is negligible when compared with the scenario during the association of a neutral guest ruled by a *K*_assoc_ two orders of magnitude lower. Such strong binding of the preorganized radical anion of naphthalimide (A–D–A) and the DAP station (D–A–D) seems completely insensitive to the competence of a non-preorganized neutral guest as the tetralactam ring.

**Fig. 6:**
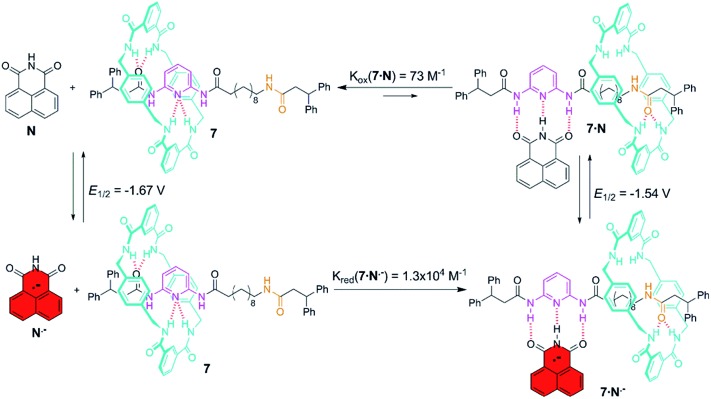
Anion radical-driven ring shuttling in the two-station [2]rotaxane **7**.

Molecular recognition of **7** with naphthalimide led to a very labile H-bonded complex in which the ring efficiently competed for the DAP binding site as the oxidized form of this acceptor–donor–acceptor is a poor H-bond donor (Fig. 6). In fact, the addition of one or two equivalents of **N** is incapable of altering the native statistical ring distribution of **7** (see Fig. S6c†). Upon reduction, the generated radical anion preferentially occupies the DAP binding site and pushes the ring to the amide station since the reduced guest is now a powerful H-bond donor. Upon re-oxidation, the original distribution is restored, the ring sitting predominantly over the DAP station.

## Conclusions

The incorporation of a di(acylamino)pyridine fragment as a binding site of hydrogen-bonded [2]rotaxanes enables the building of switchable interlocked systems in which their submolecular dynamics can be swapped between different states. By means of oxidation or protonation reactions it was possible to slow down the rotation rate of the benzylic amide macrocycle of these hydrogen-bonded rotaxanes, although through different mechanisms. Reverse transformations mediated by polymer-supported reagents cleanly recovered the native dynamics of this single binding site system. These results allowed the design and synthesis of two-station [2]rotaxanes containing a DAP-based binding site and an amido-based station which were shown to have a modest positional integrity. However, the natural statistical distribution of the macrocycle of this particular shuttle was chemically manipulated for accessing efficiently two states with an excellent positional discrimination. Moreover, the embedded DAP unit of this interlocked system tolerated a reversible anion radical recognition process giving rise to a fully controllable electrochemical switch. We deem that the extensive versatility of the described DAP-based systems underlines the capability of these hosting interlocked compounds to be incorporated into multi-responsive materials for a variety of usages including chemical sensing or drug delivery.

## References

[cit1] (a) Molecular Motors, ed. M. Schliwa, Wiley-VCH, Weinheim, 2003.

[cit2] Astumian R. D. (2007). Phys. Chem. Chem. Phys..

[cit3] Lewandowski B., De Bo G., Ward J. W., Papmeyer M., Kuschel S., Aldegunde M. J., Gramlich P. M. E., Heckmann D., Goldup S. M., D'Souza D. M., Fernandes A. E., Leigh D. A. (2013). Science.

[cit4] (b) Balzani V., Venturi M. and Credi A., Molecular Devices and Machines. A Journey into the Nanoworld, Wiley-VCH, Weinheim, Germany, 2003.

[cit5] Martinez-Cuezva A., Berna J., Orenes R.-A., Pastor A., Alajarin M. (2014). Angew. Chem., Int. Ed..

[cit6] (a) Special Issue on “Molecular Recognition”, Chem. Rev., 1997, 97, 1231 1734.11851448

[cit7] Tron A., Thornton P. J., Rocher M., Jacquot De Rouville H. P., Desvergne J. P., Kauffmann B., Buffeteau T., Cavagnat D., Tucker J. H. R., McClenaghan N. D. (2014). Org. Lett..

[cit8] Ma X., Cao J., Wang Q., Tian H. (2011). Chem. Commun..

[cit9] Zhou W., Li J., He X., Li C., Lv J., Li Y., Wang S., Liu H., Zhu D. (2008). Chem.–Eur. J..

[cit10] Gatti F. G., Leon S., Wong J. K. Y., Bottari G., Altieri A., Morales M. A. F., Teat S. J., Frochot C., Leigh D. A., Brouwer A. M., Zerbetto F. (2003). Proc. Natl. Acad. Sci. U. S. A..

[cit11] Parham A. H., Windisch B. B., Vögtle F. (1999). Eur. J. Org. Chem..

[cit12] Sanz R., Escribano J., Fernández Y., Aguado R., Pedrosa M. R., Arnaiz F. J. (2005). Synlett.

[cit13] Shi X., Barkigia K. M., Fajer J., Drain C. M. (2001). J. Org. Chem..

[cit14] Valderrey V., Escudero-Adan E. C., Ballester P. (2012). J. Am. Chem. Soc..

[cit15] Leigh D. A., Venturini A., Wilson A. J., Wong J. K. Y., Zerbetto F. (2004). Chem.–Eur. J..

[cit16] (b) For a recent example of pH-modulated nanomechanical motions see: Stadler A.-M. Lehn J.-M. P., J. Am. Chem. Soc., 2014, 136 , 3400 –3409 . 24547897 10.1021/ja408752m

[cit17] During these VT-NMR experiments the desymmetrisation of the initial spectrum at room temperature was observed. This is consistent with a likely deviation of the average plane of the macrocycle from the bisecting symmetry plane of the compound, thus avoiding repulsive non-bonding interactions of the pyridine ring of the thread and the inner periphery of the macrocycle. The coalescence of the signals associated to the aliphatic protons of the thread occurs at 223 K in deuterated dichloromethane, corresponding to an energy barrier of 9.9 kcal mol^–1^ (*T* _c_ = 223 K) for **2a**, 13.6 kcal mol^–1^ (*T* _c_ = 318 K) for **2b** and 10.5 kcal mol^–1^ (*T* _c_ = 238 K) for **2c** (see Fig. S1–S3)

[cit18] The influence of a counterion exchange on the pirouetting ring motion in amide-based [2]rotaxanes has been recently disclosed: Farras P. Escudero-Adan E. C. Viñas C. Teixidor F., Inorg. Chem., 2014, 53 , 8654 –8661 . 25061912 10.1021/ic501246e

[cit19] Bottari G., Leigh D. A., Perez E. M. (2003). J. Am. Chem. Soc..

[cit20] Altieri A., Bottari G., Dehez F., Leigh D. A., Wong J. K. Y., Zerbetto F. (2003). Angew. Chem., Int. Ed..

[cit21] Muehldorf A. V., Van Engen D., Warner J. C., Hamilton A. D. (1988). J. Am. Chem. Soc..

[cit22] Legrand Y.-M., Gray M., Cooke G., Rotello V. M. (2003). J. Am. Chem. Soc..

[cit23] Ge Y., Lilienthal R. R., Smith D. K. (1996). J. Am. Chem. Soc..

[cit24] For an intramolecular example of stabilization of an interlocked radical anion through hydrogen bonding see ref. 9*b*

[cit25] Miller S. R., Gustowski D. A., Chen Z. H., Gokel G. W., Echegoyen L., Kaifer A. E. (1988). Anal. Chem..

