# Supplementary material for: Versatile control of the submolecular motion of di(acylamino)pyridine-based [2]rotaxanes
Source: Chem Sci. 2015 Mar 18;6(5):3087–94. doi: 10.1039/c5sc00790a (PMC5490047; doi:10.1039/c5sc00790a)

Electronic Supplementary Material (ESI) for Chemical Science.
This journal is © The Royal Society of Chemistry 2015

Electronic Supplementary Information

Versatile Control of the Submolecular Motion of Di(acylamino)pyridine-Based [2]Rotaxanes

Alberto Martínez-Cuezva,[a] Aurelia Pastor,[a] Giacomo Cioncoloni,[b] Raúl-Ángel Orenes,[c] Mateo Alajarín,[a] Mark D. Symes,[b] and José Berná[a]*

[a]*Departamento de Química Orgánica, Facultad de Química, Regional Campus of International Excellence "Campus Mare Nostrum", Universidad de Murcia, E-30100 Murcia, Spain.*
[b]*WestCHEM, School of Chemistry, University of Glasgow, University Avenue, Glasgow G12 8QQ, United Kingdom.*
[c]*Sección Universitaria de Instrumentación Científica (SUIC), Servicio de Apoyo a la Investigación (SAI), Regional Campus of International Excellence "Campus Mare Nostrum", Universidad de Murcia, E-30100 Murcia, Spain.*

E-mail: *ppberna@um.es*

Table of Contents………………………………………………………………………………….. S2

1. General experimental section………………………………………………………………. S3

2. Synthesis of thread 1a ……………………………………………………………………… S4

3. Synthesis of threads 5-6 ……………………………………………………………………... S4

4. General procedure for the preparation of benzylic amide macrocycle containing [2]rotaxanes . S7

5. General procedure for the oxidation of threads and rotaxanes …………………………………. S9

6. General procedure for the reduction of pyridine *N*-oxide derivatives ……..…………………… S12

7. General procedure for the formation of picrate salts ..………………………………..…………... S14

8. General procedure for the deprotonation of picrate salts ..……………………………..……… S16

9. VT-NMR experiments for rotaxanes 2a, 2b and 2c ……………..……..……..……..………….. S16

10. Estimation of the level of occupancy of the DAP unit for rotaxanes 7-8 and 12 ………….…... S19

11. Titration experiments of threads and rotaxanes with neutral guests ……………..…………..... S20

12. Electrochemical studies ……………..………………………………………..…..…..…..…..…... S25

13. Conformational study by NMR spectroscopy for rotaxane 7, picrate salt 14 and complex 7:T S27

14. Crystal data and structure refinement for 1c and 2b ……………..……..……..……………. S32

15. ^{1}H and ^{13}C NMR spectra of synthesized compounds ……………..……..……..…………… S37

1. General Experimental Section

Unless stated otherwise, all reagents were purchased from Aldrich Chemicals and used without further purification. HPLC grade solvents (Scharlab) were nitrogen saturated and were dried and deoxygenated using an Innovative Technology Inc. Pure-Solv 400 Solvent Purification System. Column chromatography was carried out using silica gel (60 Å, 70-200 μm, SDS) as stationary phase, and TLC was performed on precoated silica gel on aluminium cards (0.25 mm thick, with fluorescent indicator 254 nm, Fluka) and observed under UV light. All melting points were determined on a Kofler hot-plate melting point apparatus and are uncorrected. ^{1}H- and ^{13}C-NMR spectra were recorded at 298 K on a Bruker Avance 300 and 400 MHz instruments. ^{1}H NMR chemical shifts are reported relative to Me_4Si and were referenced via residual proton resonances of the corresponding deuterated solvent whereas ^{13}C NMR spectra are reported relative to Me_4Si using the carbon signals of the deuterated solvent. Signals in the ^{1}H and ^{13}C NMR spectra of the synthesized compounds were assigned with the aid of DEPT, APT, or two-dimensional NMR experiments (COSY, HMQC and HMBC). Abbreviations of coupling patterns are as follows: br, broad; s, singlet; d, doublet; t, triplet; q, quadruplet; m, multiplet. Mass spectra were recorded with Agilent 5973 (EI), Agilent VL (ESI) and HPLC/MS TOF 6220 mass spectrometers.

Abbreviation list:
DCM: dichloromethane
DMSO: dimethylsulfoxide
DMAP: dimethylaminopyridine
EDCI: *N*-(3-dimethylaminopropyl)-*N′*-ethylcarbodiimide hydrochloride
TFA: trifluoroacetic acid
AcOEt: ethyl acetate
MeCN: acetonitrile

2. Synthesis of thread 1a

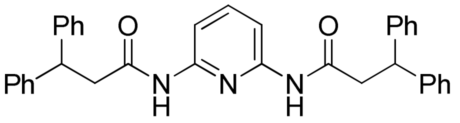

Compound **1a** was synthesized as described in A. Martinez-Cuezva, J. Berna, R.-A. Orenes, A. Pastor, M. Alajarin, *Angew. Chem., Int. Ed.* **2014**, *53*, 6762-6767, and showed identical spectroscopic data as those reported therein.

3. Synthesis of threads 5-6

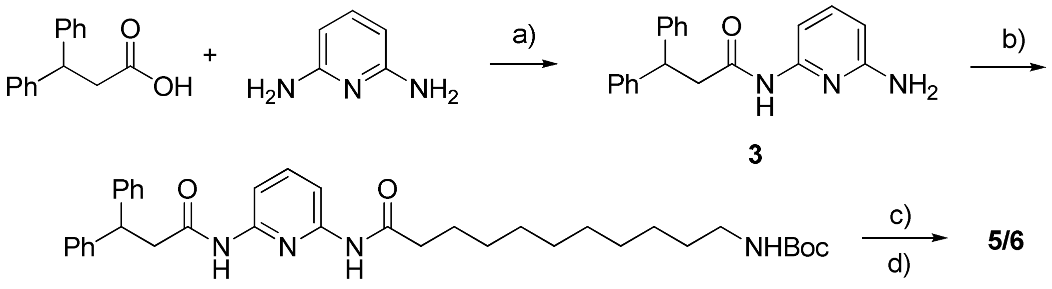

Scheme S1. a) DMAP, EDCI, DCM, rt, 48 h, 78%; b) **9**, DMAP, EDCI, DCM, rt, 48 h, 70%; c) TFA, $CHCl_3$; d) 3,3-diphenylpropanoic acid **10** or **11**, DMAP, EDCI, DCM.

N-(*N*-6-Aminopyridin-2-yl)-3,3-diphenylpropanamide, 3

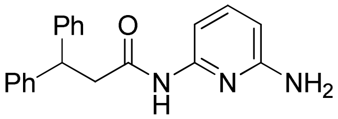

Compound **3** was synthesized as described in A. Martinez-Cuezva, J. Berna, R.-A. Orenes, A. Pastor, M. Alajarin, *Angew. Chem., Int. Ed.* **2014**, *53*, 6762-6767, and showed identical spectroscopic data as those reported therein.

11-(*tert*-Butoxycarbonylamino)undecanoic acid, 9

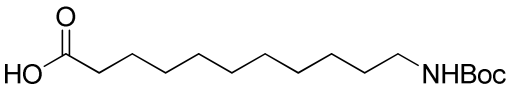

Compound **9** was synthesized as described in G. Bottari, D. A. Leigh, E. M. Pérez, *J. Am. Chem. Soc.* **2003**, *125*, 13360-13361, and showed identical spectroscopic data as those reported therein.

Compound 4

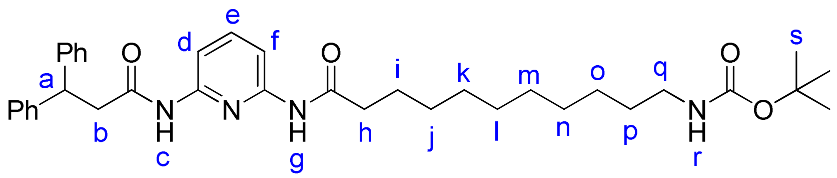

To a solution of 11-(*tert*-butoxycarbonylamino)undecanoic acid (**9**) (1.08 g, 3.50 mmol) in dry DCM (60 mL) under N_2 atmosphere was added DMAP (0.44 g, 3.6 mmol) and EDCI (0.69 g, 3.6 mmol) followed by *N*-(6-aminopyridin-2-yl)-3,3-diphenylpropanamide (**3**) (0.95 g, 3.00 mmol). The reaction mixture was stirred for 24 hours at room temperature after which time the reaction was washed with water (2 x 50 mL) and a concentrated solution of $NaHCO_3$ (2 x 50 mL). The organic phase was dried over anhydrous $MgSO_4$, concentrated under reduced pressure and purified by column chromatography on silica gel using a hexane/AcOEt (60/40) mixture as eluent, to give the title product as a colourless oil (**4**, 1.25 g, 2.1 mmol, 70%); ^{1}H NMR (400 MHz, $CDCl_3$): δ = 7.87 (d, *J* = 8.0 Hz, 1H, H_d), 7.81 (d, *J* = 8.0 Hz, 1H, H_f), 7.65-7.55 (m, 3H, NH_{c+g} + H_e), 7.31-7.24 (m, 8H, Ph), 7.21-7.17 (m, 2H, Ph), 4.69 (t, *J* = 7.8 Hz, 1H, H_a), 4.54 (bs, 1H, NH_r), 3.12-3.08 (m, 4H, H_{b+q}), 2.34 (t, *J* = 7.6 Hz, 2H, H_h), 1.76-1.66 (m, 2H, H_i), 1.50-1.40 (m, 11H), 1.45-1.25 (m, 12H); ^{13}C NMR (100 MHz, $CDCl_3$): δ = 171.7 (CO), 169.6 (CO), 156.2 (CO), 149.5 (C), 149.3 (C), 143.5 (C), 140.9 (CH), 128.8 (CH), 127.8 (CH), 126.8 (CH), 109.59 (CH), 109.56 (CH), 79.2 (C), 47.0 (CH), 44.2 (CH_2), 40.7 (CH_2), 37.9 (CH_2), 30.2 (CH_2), 29.5 (CH_2), 29.4 (CH_2), 29.33 (CH_2), 29.30 (CH_2), 29.22 (CH_2), 28.6 (CH_3), 26.9 (CH_2), 25.4 (CH_2); HRMS (ESI) calcd for $C_{36}H_{49}N_4O_4$ $[M + H]^+$ 601.3748, found 601.3775.

2,2-Diphenylethyl monosuccinate, 11

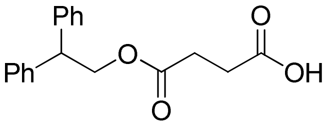

This compound was synthesized as described in A. Altieri, G. Bottari, F. Dehez, D. A. Leigh, J. K. Y. Wong, F. Zerbetto, *Angew. Chem., Int. Ed.* **2003**, *42*, 2296-2300, and showed identical spectroscopic data as those reported therein.

Thread 5

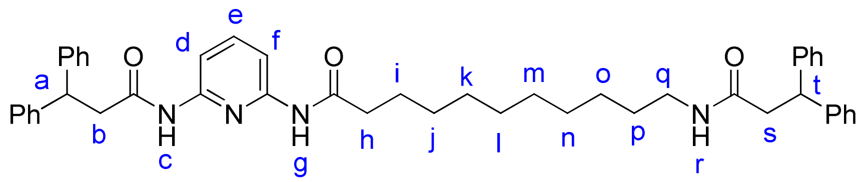

To a solution of *tert*-butyl 11-(6-(3,3-diphenylpropanamido)pyridin-2-ylamino)-11-oxoundecylcarbamate (**4**) (1.40 g, 2.33 mmol) in $CHCl_3$ (20 mL) was added TFA (2 mL). The reaction mixture was stirred for 5 hours at room temperature after which time the reaction was washed with a concentrated solution of $NaHCO_3$ (2 x 20 mL). The organic phase was dried over anhydrous $MgSO_4$ and concentrated under reduced pressure. The residue was then added to a solution of 3,3-diphenylpropanoic acid **10** (534 mg, 2.80 mmol), DMAP (341 mg, 2.80 mmol) and EDCI (477 mg, 2.50 mmol) in dry DCM (60 mL) under N_2 atmosphere. The mixture was stirred for 48 hours at room temperature after which time the reaction was washed with water (2 x 50 mL), a concentrated solution of $NaHCO_3$ (2 x 50 mL) and brine (2 x 50 mL). The organic phase was dried over anhydrous $MgSO_4$, concentrated under reduced pressure and purified by column chromatography on silica gel using a hexane/AcOEt (1/3) mixture as eluent to give the title product as a white solid (**5**, 650 mg, 0.92 mmol, 39%). M.p. 63-64 ºC; ^{1}H NMR (300 MHz, CD_2Cl_2): δ = 7.88 (s, 1H, NH_c), 7.85 (d, J = 8.1 Hz, 1H, H_d), 7.81 (s, 1H, NH_g), 7.78 (d, J = 8.1 Hz, 1H, H_f), 7.62 (t, J = 8.1 Hz, 1H, H_e), 7.39-7.20 (m, 20H, Ph), 5.51 (t, J = 5.3 Hz, 1H, NH_r), 4.65 (t, J = 7.8 Hz, 1H, H_a), 4.56 (t, J = 7.9 Hz, 1H, H_t), 3.10 (d, J = 7.9 Hz, 2H, H_b), 3.14-3.02 (m, 2H, H_q), 2.87 (d, J = 7.9 Hz, 2H, H_s), 2.32 (t, J = 7.5 Hz, 2H, H_h), 1.70-1.62 (m, 2H, H_i), 1.40-1.15 (m, 14H); ^{13}C NMR (75 MHz, CD_2Cl_2): δ = 172.2 (CO), 171.2 (CO), 169.9 (CO), 150.4 (C), 150.1 (C), 144.6 (C), 144.3 (C), 141.0 (CH), 129.2 (CH), 129.1 (CH), 128.20 (CH), 128.16 (CH), 127.1 (CH), 127.0 (CH), 109.6 (CH), 109.5 (CH), 47.9 (CH_2), 47.4 (CH_2), 44.0 (CH_2), 43.5 (CH_2), 39.8 (CH_2), 38.1 (CH_2), 29.9 (CH_2), 29.8 (CH_2), 29.7 (CH_2), 29.6 (CH_2), 27.1 (CH_2), 25.8 (CH_2); HRMS (ESI) calcd for $C_{46}H_{52}N_4O_3$ $[M + H]^+$ 709.4112, found 709.4117.

Thread 6

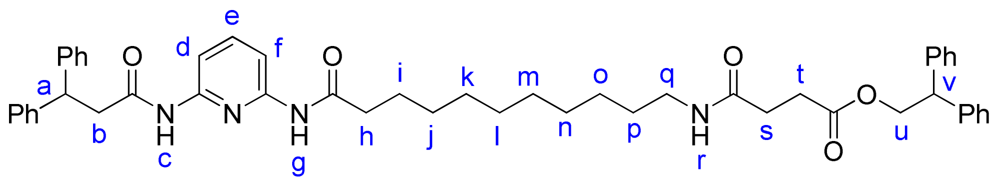

To a solution of *tert*-butyl 11-(6-(3,3-diphenylpropanamido)pyridin-2-ylamino)-11-oxoundecylcarbamate (**4**) (1.30 g, 2.25 mmol) in $CHCl_3$ (20 mL) was added TFA (2 mL). The reaction mixture was stirred for 5

hours at room temperature after which time the reaction was washed with a concentrated solution of $NaHCO_3$ (2 x 20 mL). The organic phase was dried over anhydrous $MgSO_4$ and concentrated under reduced pressure. The residue was then added to a solution of 2,2-diphenylethyl monosuccinate **11** (655 mg, 2.20 mmol), DMAP (305 mg, 2.50 mmol) and EDCI (477 mg, 2.50 mmol) in dry DCM (50 mL) under N_2 atmosphere. The reaction mixture was stirred for 48 hours at room temperature after which time the reaction was washed with water (2 x 50 mL),a concentrated solution of $NaHCO_3$ (2 x 50 mL) and brine (2 x 50 mL). The organic phase was dried over anhydrous $MgSO_4$, concentrated under reduced pressure and purified by column chromatography on silica gel using a hexane/AcOEt (1/1) mixture as eluent to give the title product as a white solid (**6**, 650 mg, 0.83 mmol, 38%). M.p. 65-66 ºC; ^{1}H NMR (300 MHz, $CDCl_3$): δ = 8.20-7.65 (m, 4H, NH_{c+g}+ H_{d+f}), 7.61 (t, J = 8.0 Hz, 1H, H_e), 7.32-7.06 (m, 20H, Ph), 5.59 (s, 1H, NH_r), 4.69 (t, J = 7.7 Hz, 1H, H_a), 4.61 (d, J = 7.6 Hz, 2H, H_u), 4.32 (t, J = 7.6 Hz, 1H, H_v), 3.20-3.04 (m, 4H, H_{b+q}), 2.58 (t, J = 6.8 Hz, 2H, H_t), 2.33 (t, J = 6.8 Hz, 2H, H_s), 2.38-2.28 (m, 2H, H_h) 1.74-1.61 (m, 2H, H_i), 1.47-1.37 (m, 2H, H_p), 1.32-1.15 (m, 12H); ^{13}C NMR (75 MHz, $CDCl_3$): δ = 173.1 (CO), 172.3 (CO), 171.4 (CO), 170.2 (CO), 149.5 (C), 143.6 (C), 140.9 (C), 140.5 (C), 128.5 (2 x CH), 128.1 (CH), 127.6 (CH), 126.8 (CH), 126.5 (CH), 109.4 (2 x CH), 66.9 (CH_2), 49.7 (CH), 46.7 (CH), 43.3 (CH_2), 39.6 (CH_2), 37.3 (CH_2), 30.9 (CH_2), 29.7 (CH_2), 29.4 (CH_2), 29.2 (CH_2), 29.15 (CH_2), 29.1 (CH_2), 29.0 (CH_2), 26.7 (CH_2), 25.2 (CH_2); HRMS (ESI) calcd for $C_{49}H_{57}N_4O_5$ $[M + H]^+$ 781.4323, found 781.4354.

4. General procedure for the preparation of benzylic amide macrocycle containing [2]rotaxanes

The thread (1 mmol) and Et_3N (24 equiv.) in anhydrous $CHCl_3$ (300 mL) were stirred vigorously whilst solutions of *p*-xylylene diamine (12 equiv.) in anhydrous $CHCl_3$ (20 mL) and the corresponding acid dichloride (12 equiv.) in anhydrous $CHCl_3$ (20 mL) were simultaneously added over a period of 5 h using motor-driven syringe pumps. After a further 4 h the resulting suspension was filtered through a Celite® pad, washed with water (2 x 50 mL), a saturated solution of $NaHCO_3$ (2 x 50 mL) and brine (2 x 50 mL). The organic phase was dried over $MgSO_4$ and the solvent removed under reduced pressure. The resulting solid was subjected to column chromatography (silica gel) to yield unconsumed thread, [2]rotaxane and [2]catenane. The formation of the [3]rotaxane was not observed in any case.

Rotaxane 2a

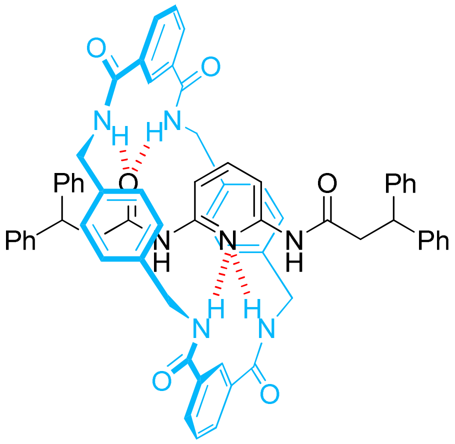

Rotaxane **2a** was synthesized as described in A. Martínez-Cuezva, J. Berna, R.-A. Orenes, A. Pastor, M. Alajarin, *Angew. Chem., Int. Ed.* **2014**, *53*, 6762-6767, and showed identical spectroscopic data as those reported therein.

Rotaxane 7

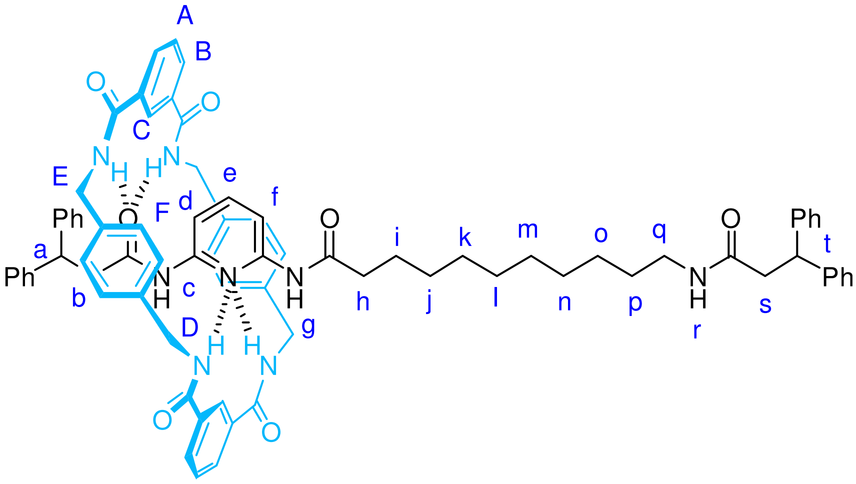

Rotaxane **7** was obtained following the described method from thread **5** (600 mg, 0.85 mmol). The solid crude was subjected to column chromatography on silica gel using AcOEt/hexane (80/20) mixture as eluent to give the title product as a white solid (**7**, 273 mg, 0.22 mmol, 28%). M.p. 120-122 ºC; ^{1}H NMR (400 MHz, CD_2Cl_2): δ = 8.41 (s, 2H, H_C), 8.20-8.14 (m, 5H, H_B+NH_c), 7.69 (s, 1H, NH_g), 7.60 (t, *J* = 7.8 Hz, 2H, H_A), 7.55-7.49 (m, 2H, H_{d+f}), 7.48-7.40 (m, 5H, NH_D+H_e), 7.28-7.12 (m, 20H, Ph), 6.89 (s, 8H, H_F), 6.21 (t, *J* = 5.3 Hz, 1H, NH_r), 4.48-4.30 (m, 10H, H_{E+a+t}), 2.93 (dd, *J* = 12.9, 6.6 Hz, 2H, H_q), 2.83 (d, *J* = 7.6 Hz, 2H, H_b), 2.77 (d, *J* = 7.7 Hz, 2H, H_s), 1.67 (t, *J* = 7.4 Hz, 2H, H_h), 1.25-0.90 (m, 16H); ^{13}C NMR (100 MHz, CD_2Cl_2) δ = 173.6 (CO), 171.9 (CO), 171.1 (CO), 166.9 (CO), 149.9 (C), 149.8 (C), 144.6 (C), 144.4 (C), 141.0 (CH), 137.7 (C), 134.7 (C), 131.5(CH), 129.8 (CH), 129.14 (CH), 129.08 (CH), 129.06 (CH), 128.2 (CH), 128.1 (CH), 127.1 (CH), 127.0 (CH), 126.1 (CH), 109.8 (2 x CH), 47.8 (CH), 47.2 (CH), 44.7 (CH_2), 43.1 (CH_2), 42.9 (CH_2), 39.9 (CH_2), 37.2 (CH_2), 29.7 (CH_2), 29.6 (CH_2),

29.5 (CH_2), 29.4 (CH_2), 29.3 (CH_2), 29.3 (CH_2), 27.0 (CH_2), 25.2 (CH_2); HRMS (ESI) calcd for $C_{78}H_{81}N_8O_7$ $[M + H]^+$ 1241.6223, found 1241.6220.

Rotaxane 8

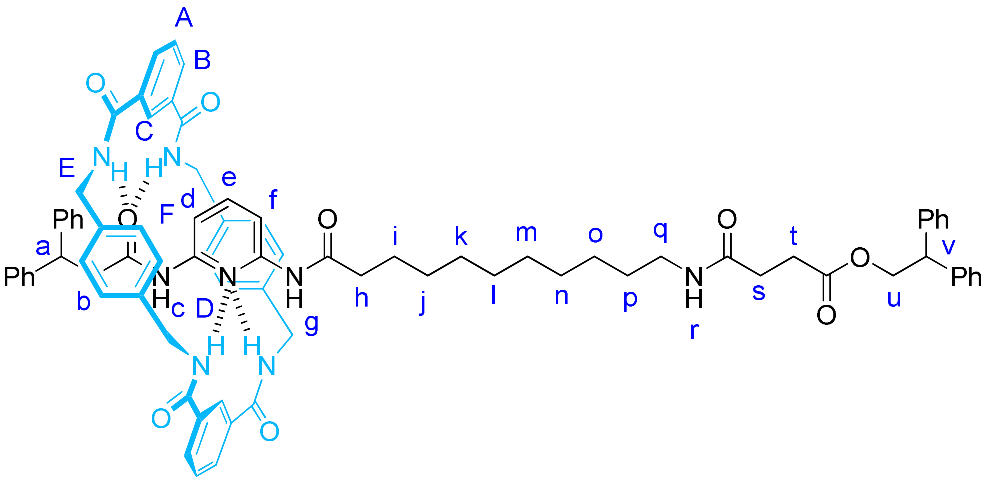

Rotaxane **8** was obtained following the described method from thread **6** (0.60 g, 0.77 mmol). The solid crude was subjected to column chromatography on silica gel using AcOEt/hexane (80/20) mixture as eluent to give the title product as a white solid (**8**, 0.140 g, 0.11 mmol, 23%). M.p. 131-132 ºC; ^{1}H NMR (300 MHz, CD_2Cl_2): δ = 8.34 (s, 1H, NH_c), 8.32 (s, 2H, H_C), 8.15 (dd, J = 7.8, 1.5 Hz, 4H, H_B), 7.88 (s, 1H, NH_g), 7.73-7.64 (m, 2H, H_{d+f}), 7.58 (t, J = 7.8 Hz, 2H, H_A), 7.55 (t, J = 8.1 Hz, 1H, H_e), 7.46 (t, J = 5.1 Hz, 4H, NH_D), 7.32-7.12 (m, 20H, Ph), 6.99 (s, 8H, H_F), 6.51 (t, J = 5.4 Hz, 1H, NH_r), 4.55 (t, J = 7.7 Hz, 1H, H_a), 4.50-4.40 (m, 10H, H_{E+u}), 4.21 (t, J = 7.5 Hz, 1H, H_v), 3.03 (d, J = 7.7 Hz, 2H, H_b), 2.95 (dd, J = 13.0, 6.7 Hz, 2H, H_q), 2.02 (t, J = 7.5 Hz, 2H, H_h), 1.82-1.73 (m, 2H, H_t), 1.52-1.32 (m, 6H, H_{i+p+s}), 1.22-1.10 (m, 12H); ^{13}C NMR (75 MHz, CD_2Cl_2) δ = 174.4 (CO), 172.8 (CO), 172.5 (CO), 170.6 (CO), 167.0 (CO), 150.2 (C), 144.4 (C), 141.6 (C), 140.9 (CH), 138.1 (C), 134.8 (C), 131.6 (CH), 129.7 (CH), 129.4 (CH), 129.2 (CH), 129.1 (CH), 128.6 (CH), 128.2 (CH), 127.5 (CH), 127.1 (CH), 125.4 (CH), 109.7 (2 x CH), 67.9 (CH_2), 50.2 (CH), 47.3 (CH), 44.6 (CH_2), 43.5 (CH_2), 40.3 (CH_2), 37.7 (CH_2), 30.2 (CH_2), 29.7 (CH_2), 29.6 (CH_2), 29.5 (CH_2), 29.4 (CH_2), 27.2 (CH_2), 25.6 (CH_2); HRMS (ESI) calcd for $C_{81}H_{85}N_8O_9$ $[M + H]^+$ 1313.6434, found 1313.6441.

5. General procedure for the oxidation of threads and rotaxanes.

To a solution of the corresponding thread or rotaxane (1 equiv.) in $CHCl_3$ (2 mL) was added *m*-CPBA (2 equiv.). The reaction mixture was stirred overnight at room temperature after which time Amberlyst A-21® basic resin (1 gr/mmol $_{\text{RTX or Thread}}$) was added. The mixture was stirred for 1 hour and then filtered. The residue was washed with 1 mL of $CHCl_3$. The organic phase was concentrated under reduced pressure, yielding the titled compounds.

Thread 1b

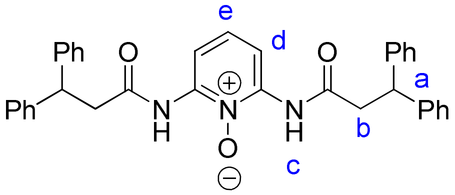

Thread **1b** (61.1 mg, 0.11 mmol, 91%) was obtained as a white solid following the described method from thread **1a** (65 mg, 0.124 mmol). M.p. 224-225 ºC; ^{1}H NMR (400 MHz, CD_2Cl_2, 298 K): δ = 9.94 (s, 2H, NH_c), 8.01 (d, J = 8.6 Hz, 2H, H_d), 7.33-7.28 (m, 17H, Ph), 7.24-7.18 (m, 4H, Ph+H_e), 4.68 (t, J = 7.8 Hz, 2H, H_a), 3.30 (d, J = 7.8 Hz, 4H, H_b); ^{13}C NMR (100 MHz, CD_2Cl_2, 298 K): δ = 170.3 (CO), 144.1 (C), 142.7 (C), 130.2 (CH), 129.2 (CH), 128.1 (CH), 127.2 (CH), 108.9 (CH), 47.3 (CH), 44.0 (CH_2); HRMS (ESI) calcd for $C_{35}H_{32}N_3O_3$ $[M + H]^+$ 542.2438, found 542.2456.

Thread 13

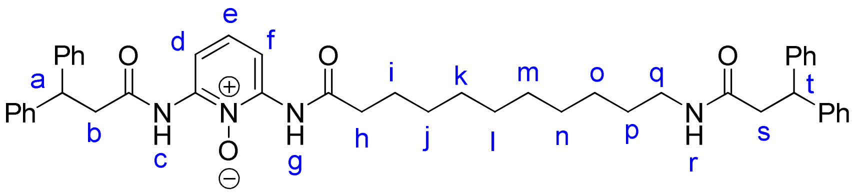

Thread **13** (19.6 mg, 0.027 mmol, 96%) was obtained as a white solid following the described method from thread **5** (20 mg, 0.028 mmol). M.p. 70-71 ºC; ^{1}H NMR (400 MHz, $CDCl_3$): δ = 9.83 (s, 1H, NH_c), 9.77 (s, 1H, NH_g), 8.10 (dd, J = 8.5 Hz, 1.7 Hz, 1H, H_d), 8.04 (dd, J = 8.6 Hz, 1.7 Hz, 1H, H_f), 7.35-7.15 (m, 21H, Ph + H_e), 5.23 (m, 1H, NH_r), 4.70 (t, J = 7.8 Hz, 1H, H_a), 4.56 (t, J = 7.8 Hz, 1H, H_t), 3.27 (d, J = 7.8 Hz, 2H, H_b), 3.08 (dd, J = 12.7, 6.8 Hz, 2H, H_q), 2.88 (d, J = 7.8 Hz, 2H, H_s), 2.50 (t, J = 7.5 Hz, 2H, H_h), 1.78-1.69 (m, 2H, H_i), 1.40-1.00 (m, 14H); ^{13}C NMR (100 MHz, $CDCl_3$): δ = 172.0 (CO), 171.2 (CO), 169.8 (CO), 143.8 (C), 143.2 (C), 142.3 (C), 142.2 (C), 130.5 (CH), 128.9 (CH), 128.7 (CH), 127.9 (CH), 127.8 (CH), 126.9 (CH), 126.7 (CH), 107.9 (2 x CH), 47.7 (CH), 47.0 (CH), 44.2 (CH_2), 43.6 (CH_2), 39.6 (CH_2), 38.1 (CH_2), 29.8 (CH_2), 29.5 (CH_2), 29.4 (CH_2), 29.36 (CH_2), 29.3 (CH_2), 29.2 (CH_2), 26.7 (CH_2), 25.2 (CH_2); HRMS (ESI) calcd for $C_{46}H_{53}N_4O_4$ $[M + H]^+$ 725.4061, found 725.4050.

Rotaxane 2b

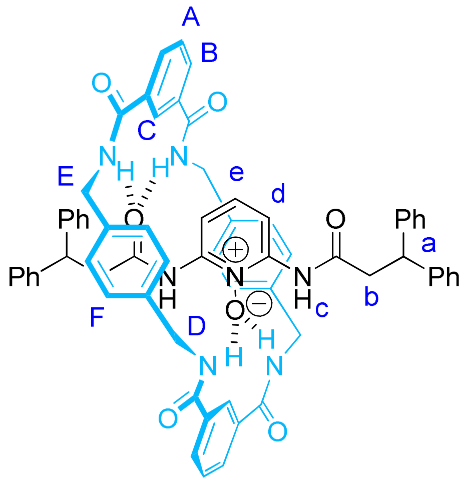

Rotaxane **2b** (40 mg, 0.037 mmol, 98%) was obtained as a white solid following the described method from rotaxane **2a** (40 mg, 0.038 mmol). M.p. > 300 ºC; ^{1}H NMR (400 MHz, $C_2D_2Cl_4$, **373K**): δ = 9.05 (s, 2H, NH_c), 8.50 (t, J = 1.5 Hz, 2H, H_C), 8.40 (dd, J = 7.8 Hz, 1.5 Hz, 4H, H_B), 7.82 (d, J = 8.5 Hz, 2H, H_d), 7.76 (t, J = 7.8 Hz, 2H, H_A), 7.29-7.19 (m, 13H, Ph+H_e), 7.14 (t, J = 5.0 Hz, 4H, NH_D), 7.08-7.02 (m, 8H, Ph), 6.68 (s, 8H, H_F), 4.40 (d, J = 5.3 Hz, 4H, H_E), 4.29 (t, J = 7.2 Hz, 2H, H_a), 2.75 (t, J = 7.2 Hz, 4H, H_b); ^{13}C NMR (100 MHz, $C_2D_2Cl_4$, **373K**) δ = 173.3 (CO), 168.5 (CO), 145.7 (C), 144.3 (C), 140.3 (C), 137.2 (C), 134.8 (CH), 133.7 (CH), 132.6 (CH), 131.6 (CH), 131.5 (CH), 130.2 (CH), 129.8 (CH), 125.6 (CH), 110.8 (CH), 49.3 (CH), 46.9 (CH_2), 46.1 (CH_2); HRMS (ESI) calcd for $C_{67}H_{60}N_7O_7$ $[M + H]^+$ 1074.4549, found 1074.4536.

<u>NOTE</u>: Rotaxane **2b** (14% yield) was also synthesized following the described method for the synthesis of [2]rotaxanes from thread **1b**.

Rotaxane 12

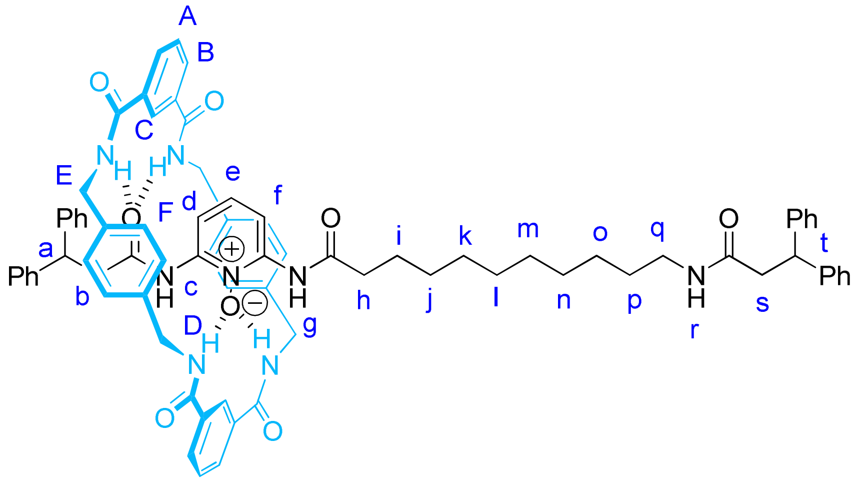

Rotaxane **12** (50.6 mg, 0.050 mmol, 99%) was obtained as a white solid following the described method from rotaxane **7** (50 mg, 0.040 mmol). M.p. 138-139 ºC; ^{1}H NMR (400 MHz, $C_2D_2Cl_4$, **373K**): δ = 9.31 (s, 1H, NH_c), 9.06 (s, 1H, NH_g), 8.49 (s, 2H, H_C), 8.33 (dd, *J* = 7.8 Hz, 1.6 Hz, 4H, H_B), 8.01 (d, *J* = 8.5 Hz, 1H, H_d), 7.85 (d, *J* = 8.5 Hz, 1H, H_f), 7.71 (t, *J* = 7.8 Hz, 2H, H_A), 7.35 (t, *J* = 7.8 Hz, 1H, H_e), 7.32-7.12 (m, 24H, Ph+ NH_D), 6.88 (s, 8H, H_F), 5.44 (s, 1H, NH_r), 4.59 (dd, *J* = 14.1, 5.5 Hz, 4H, H_E), 4.50 (t, *J* = 7.6 Hz, 1H, H_t), 4.46 (t, *J* = 7.4 Hz, 1H, H_a), 4.35 (dd, *J* = 14.1, 4.7 Hz, 4H, H_E), 3.05-2.92 (m, 4H, H_{q+b}), 2.82 (d, *J* = 7.6 Hz, 2H, H_s), 1.96-1.84 (m, 2H, H_h), 1.36-1.04 (m, 16H); ^{13}C NMR (100 MHz, $C_2D_2Cl_4$, **373K**) δ = 172.5 (CO), 170.6 (CO), 169.9 (CO), 165.4 (CO), 143.9 (C), 142.6 (C), 141.6 (C), 141.3 (C), 137.4 (C), 133.9 (C), 131.5 (CH), 130.4 (CH), 129.3 (CH), 128.6 (CH), 128.5 (CH), 128.2 (CH), 127.5 (CH), 127.2 (CH), 126.7 (CH), 126.1 (CH), 122.8 (CH), 107.9 (CH), 107.5 (CH), 47.2 (CH), 46.7 (CH), 43.9 (CH_2), 43.6 (CH_2), 42.8 (CH_2), 39.4 (CH_2), 37.1 (CH_2), 29.1 (CH_2), 28.8 (CH_2), 28.7 (CH_2), 28.6 (CH_2), 28.5 (2 x CH_2), 26.4 (CH_2), 24.3 (CH_2); HRMS (ESI) calcd for $C_{78}H_{81}N_8O_8$ $[M + H]^+$ 1257.6172, found 1257.4506.

6. General procedure for the reduction of pyridine *N*-oxide derivatives[1]

To a solution of the corresponding pyridine *N*-oxide (1 equiv.) in MeCN, PPh_3 (polymer-supported, 3 mmol PPh_3/g polymer) (15 equiv.) and $MoO_2Cl_2(DMF)_2$ (10 mol%) were added. The reaction mixture was heated for 2 hours in a closed-vial after which time the reaction was complete. The solution was diluted with a solution of $CHCl_3$:MeOH (95:5) (2 mL) and filtered through a pad of silica. The residue was concentrated under reduced pressure to give the corresponding di(acylamino)pyridine derivative.

Thread 1a

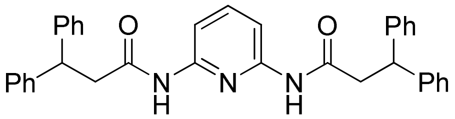

Thread **1a** (18.5 mg, 0.035 mmol, 95%) was obtained following the described method from thread **1b** (20 mg, 0.037 mmol), using PPh_3 (polymer-supported) (15 equiv.), $MoO_2Cl_2(DMF)_2$ (10 mol%) in 1 mL of MeCN at 100ºC for 2 hours.

[1] Related procedure for the reduction of *N*-oxides was described in: R. Sanz, J. Escribano, Y. Fernández, R. Aguado, M. R. Pedrosa, F. J. Arnáiz, *Synlett*, **2005**, 1389-1392.

Rotaxane 2a

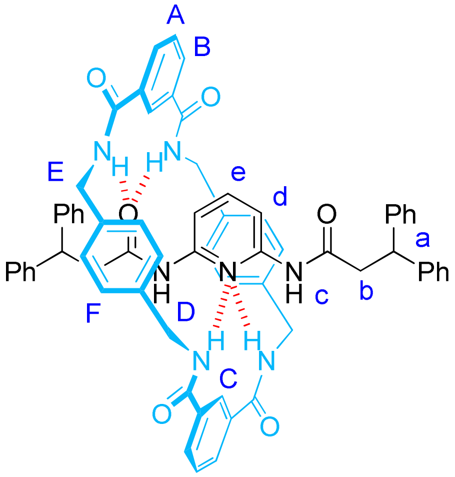

Rotaxane **2a** (9 mg, 0.0085 mmol, 96%) was obtained following the described method from rotaxane **2b** (9.5 mg, 0.009 mmol), using PPh_3 (polymer-supported) (15 equiv.), $MoO_2Cl_2(DMF)_2$ (10 mol%) in 1 mL of MeCN:toluene (1:1) at 120ºC for 4 hours.

Rotaxane 7

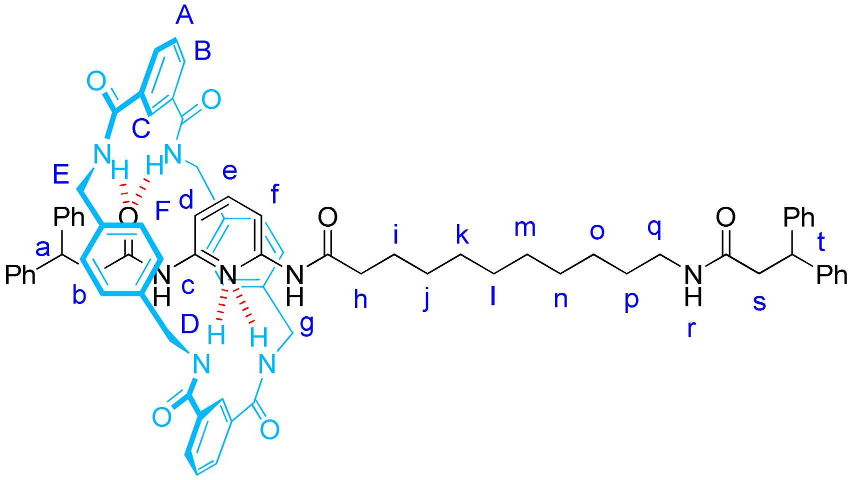

Rotaxane **7** (18 mg, 0.015 mmol, 91%) was obtained following the described method from rotaxane **12** (20 mg, 0.016 mmol), using PPh_3 (polymer-supported) (15 equiv.), $MoO_2Cl_2(DMF)_2$ (10 mol%) in 2 mL of MeCN:toluene (1:1) at 120 ºC for 5 hours.

7. General procedure for the formation of picrate salts

To a solution of the corresponding rotaxane (1 equiv) in $CHCl_3$ (1 mL), picric acid (1 equiv) was added. The reaction mixture was stirred 3 h at room temperature after this time the solution was concentrated under reduced pressure and dried under vacuum, yielding the corresponding salt quantitatively.

Picrate salt 1c

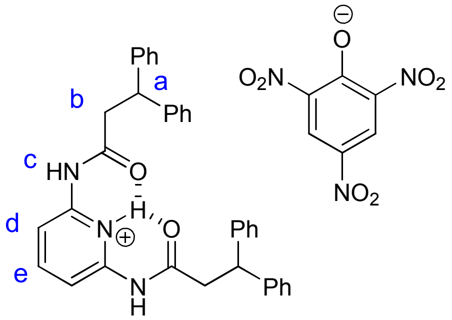

Salt **1c** was obtained following the described method from thread **1a** and picric acid in $CHCl_3$. M.p. 182-183 ºC; ^{1}H NMR (300 MHz, CD_2Cl_2): δ = 9.34 (s, 1H, NH_c), 9.06 (s, 2H, picrate), 7.58 (t, J = 8.2 Hz, 1H, H_e), 7.32-7.14 (m, 22H, Ph + H_d), 4.63 (t, J = 7.7 Hz, 2H, H_a), 3.18 (d, J = 7.7 Hz, 2H, H_b); ^{13}C NMR (75 MHz, CD_2Cl_2) δ = 171.9 (CO), 147.4 (C), 144.2 (C), 143.8 (CH), 139.5 (C), 134.9 (C), 129.2 (CH), 128.1 (CH), 127.3 (CH), 127.1 (CH), 109.2 (CH), 47.1 (CH), 43.8 (CH_2).

Picrate salt 2c

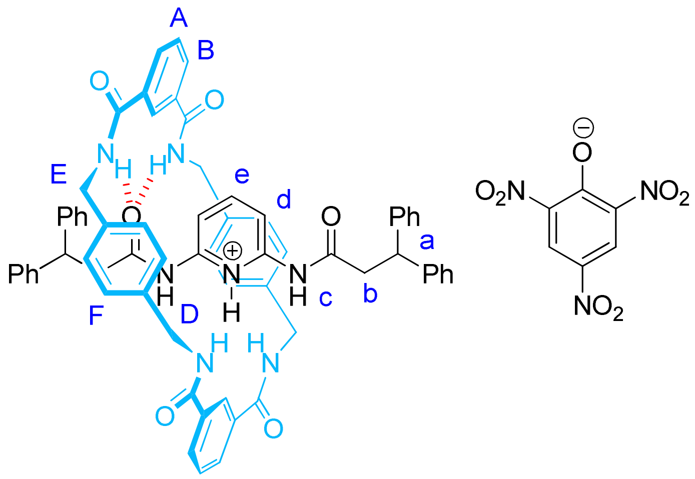

Salt **2c** was obtained following the described method from rotaxane **2a** and picric acid in $CHCl_3$; M.p. 151-152 ºC; ^{1}H NMR (300 MHz, CD_2Cl_2): δ = 9.15 (s, 2H, picrate), 8.37 (s, 2H, H_C), 8.19 (dd, J = 7.7 Hz, 1.6 Hz, 4H, H_B), 7.71 (t, J = 7.7 Hz, 2H, H_A), 7.49-7.41 (m, 1H, H_e), 7.37 (t, J = 7.5 Hz, 2H, H_d), 7.26-7.00 (m, 20H, Ph), 9.92 (s, 4H, NH_D), 6.73 (s, 8H, H_F), 4.34 (d, J = 4.9 Hz, 4H, H_E), 4.18-4.08 (m, 2H, H_a), 2.60 (d, J = 6.9 Hz, 2H, H_b); ^{13}C NMR (100 MHz, CD_2Cl_2) δ = 171.8 (CO), 167.1 (CO), 155.2

(C), 148.6 (C), 144.2 (C), 142.2 (CH), 138.4 (C), 137.5 (C), 134.9 (C), 131.3 (CH), 130.0 (CH), 129.2 (CH), 129.0 (CH), 128.0 (CH), 127.2 (CH), 127.0 (CH), 126.6 (CH), 109.7 (CH), 46.8 (CH), 44.8 (CH_2), 42.7 (CH_2).

Picrate salt 15

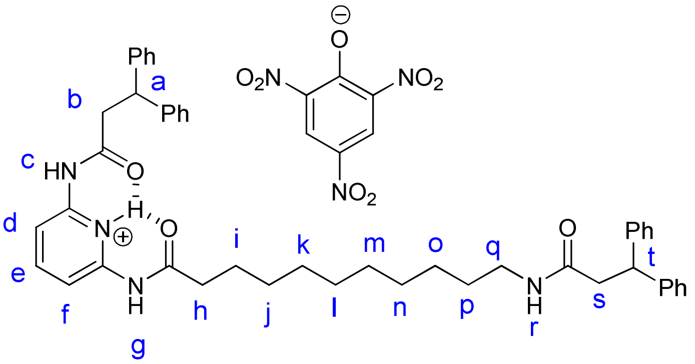

Salt **15** was obtained following the described method from thread **5** and picric acid in $CHCl_3$. M.p. 70-72 ºC; ^{1}H NMR (400 MHz, CD_2Cl_2): δ = 11.10 (s, 1H, NH_c), 10.50 (s, 1H, NH_g), 9.05 (s, 2H, picrate), 7.84 (t, J = 8.3 Hz, 1H, H_e), 7.41-7.10 (m, 22H, Ph + H_{d+f}), 5.67 (s, 1H, NH_r), 4.70 (t, J = 7.9 Hz, 1H, H_a), 4.49 (t, J = 7.9 Hz, 1H, H_t), 3.31 (d, J = 7.9 Hz, 2H, H_b), 3.13 (dd, J = 13.0, 6.9 Hz, 2H, H_q), 2.96 (d, J = 7.6 Hz, 2H, H_s), 2.42-2.36 (m, 2H, H_h), 1.75-1.65 (m, 2H, H_i), 1.20-0.60 (m, 14H); ^{13}C NMR (100 MHz, CD_2Cl_2) δ = 176.6 (CO), 174.6 (CO), 172.5 (CO), 159.1 (C), 146.6 (C), 145.9 (CH), 144.0 (C), 143.8 (C), 140.7 (C), 132.4 (C), 129.2 (CH), 128.0 (CH), 127.3 (CH), 127.2 (CH), 126.9 (CH), 109.1 (CH), 108.4 (CH), 48.2 (CH), 46.9 (CH), 43.4 (CH_2), 43.2 (CH_2), 40.0 (CH_2), 37.4 (CH_2), 30.2 (CH_2), 29.5 (CH_2), 29.1 (CH_2), 29.07 (CH_2), 29.0 (CH_2), 28.96 (CH_2), 26.7 (CH_2), 24.9 (CH_2).

Picrate salt 14

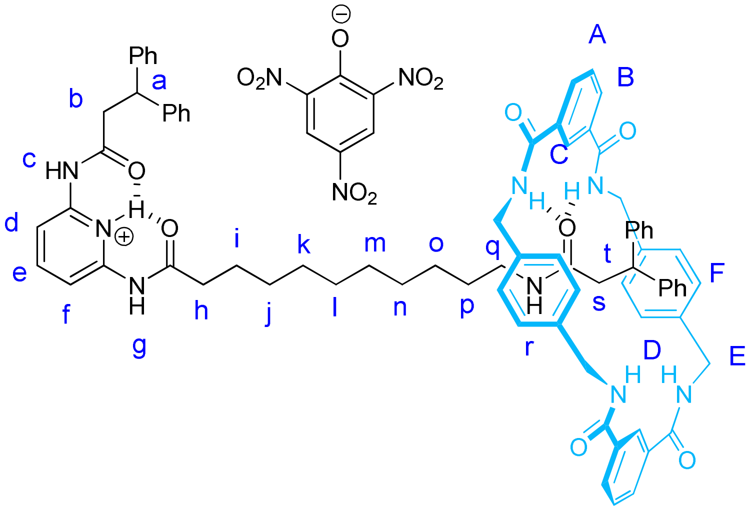

Salt **14** was obtained following the described method from rotaxane **7** and picric acid in $CHCl_3$. M.p. 123-125 ºC; ^{1}H NMR (300 MHz, CD_2Cl_2): δ = 12.06 (s, 1H, NH_c), 10.51 (s, 1H, NH_g), 8.99 (s, 2H, picrate),

8.26 (s, 2H, H_C), 8.13 (dd, J = 7.8 Hz, 1.5 Hz, 4H, H_B), 7.98 (t, J = 8.3 Hz, 1H, H_e), 7.59 (t, J = 7.8 Hz, 2H, H_A), 7.51-7.42 (m, 4H, NH_D), 7.38 (d, J = 8.3 Hz, 1H, H_d), 7.25-7.05 (m, 21H, Ph + H_f), 6.99 (s, 8H, H_F), 6.20 (t, J = 6.1 Hz, 1H, NH_r), 4.57 (dd, J = 14.4, 6.3 Hz, 4H, H_E), 4.42 (t, J = 7.7 Hz, 1H, H_a), 4.30-4.15 (m, 5H, H_{E+t}), 3.25 (d, J = 7.7 Hz, 2H, H_b), 2.78 (dd, J = 13.7, 6.1 Hz, 2H, H_q), 2.64 (d, J = 7.6 Hz, 2H, H_s), 1.60-1.48 (m, 2H, H_h), 1.20-0.60 (m, 16H); ^{13}C NMR (75 MHz, CD_2Cl_2) δ = 176.4 (CO), 175.1 (CO), 172.4 (CO), 166.9 (CO), 158.5 (C), 146.6 (C), 146.1 (C), 145.5 (C), 144.5 (C), 143.7 (C), 140.3 (C), 138.4 (C), 134.6 (C), 133.3 (CH), 131.5 (CH), 130.0 (CH), 129.2 (CH), 129.1 (CH), 129.0 (CH), 128.1 (CH), 128.0 (CH), 127.2 (CH), 127.1 (CH), 126.9 (CH), 125.7 (CH), 110.0 (CH), 108.8 (CH), 47.6 (CH), 46.9 (CH), 44.5 (CH_2), 43.0 (CH_2), 42.5 (CH_2), 39.8 (CH_2), 36.8 (CH_2), 29.3 (CH_2), 29.19 (CH_2), 29.17 (CH_2), 28.7 (CH_2), 28.5 (CH_2), 28.4 (CH_2), 26.7 (CH), 24.2 (CH_2).

8. General procedure for the deprotonation of picrate salts

To a solution of the corresponding rotaxane/thread picrate salt (1 equiv) in $CHCl_3$ (1 mL), Amberlyst A21® resin (1 gr/$mmol_{salt}$) was added. The reaction mixture was stirred 1 h at room temperature after which time the solution was filtered and the filtrate was concentrated under reduced pressure and dried under vacuum, yielding quantitatively the corresponding (diacylaminopyridine) derivative.

9. VT-NMR experiments for rotaxanes 2a, 2b and 2c

Variation temperature ^{1}H NMR experiments were performed for rotaxanes **2a**, **2b** and **2c** in order to get valuable information about the rotational motion of the polyamide macrocycle and estimate the relative strength of the intercomponent interactions between the macrocycle and the corresponding threads. Free energies of activation for the rotation of the macrocycle were calculated using the Eyring equation, $\Delta G_c^{\neq} = -RT_c \cdot ln(k_c h/k_b T_c)$, where $k_c = \pi\sqrt{(\Delta\upsilon^2 + 6J^2)}/\sqrt{2}$ and R, h and k_b are the gas, Planck and Boltzmann constants, respectively.[2]

The temperature dependence of the ^{1}H NMR spectrum of rotaxane **2a** was studied in CD_2Cl_2 (from 298K to 193K). Signals referred to the methylene protons of the macrocycle (H_E) did not split up. On the other hand a coalescence of the signals associated to the aliphatic protons H_a and H_b of the thread was observed, occurring at 223 K.

---

[2] a) F. P. Gasparro, N. H. Kolodny, *J. Chem. Ed.* **1977**, *54,* 258-261; b) *Dynamic NMR Spectroscopy,* ed. J. Sandström, Academic Press, New York, **1982**; c) M. Ōki, in *Applications of Dynamic NMR Spectroscopy to Organic Chemistry,* VCH, Weinheim, **1985**.

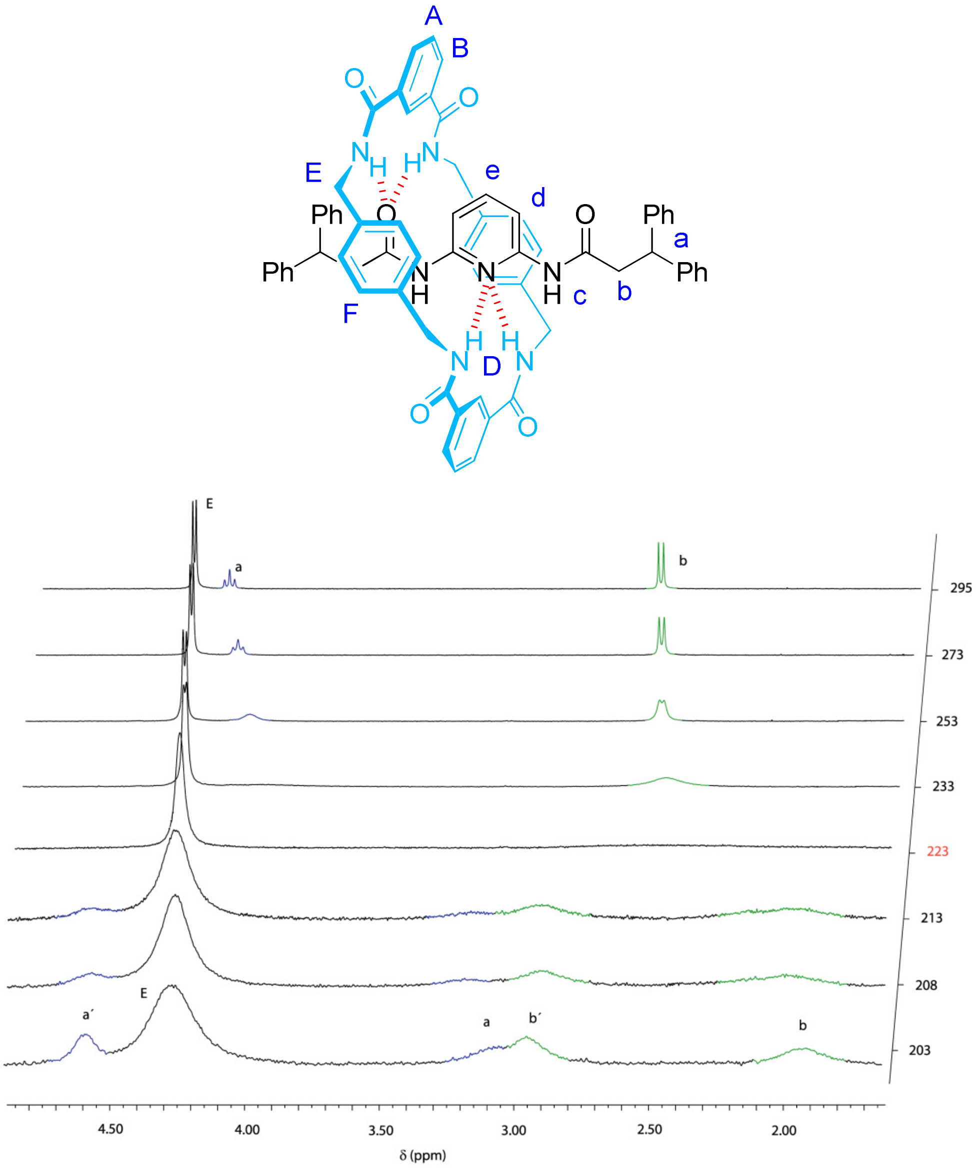

Figure 1. Partial VT-^{1}H NMR spectra (400MHz, CD_2Cl_2) of **2a**.

In the case of the *N*-oxide rotaxane **2b** the range of temperature oscillated between 393 K (in $C_2D_2Cl_4$) and 233 K. In this case we can observe two temperatures where different signals coalesced. At 298 K the signal associated with the methylene protons of the macrocycle (H_E) coalesced, associated with an energy barrier for the macrocycle pirouetting of 13.6 kcal·mol^{-1}. Again a splitting of the signals for the stoppers (H_a and H_b) was observed at 318 K.

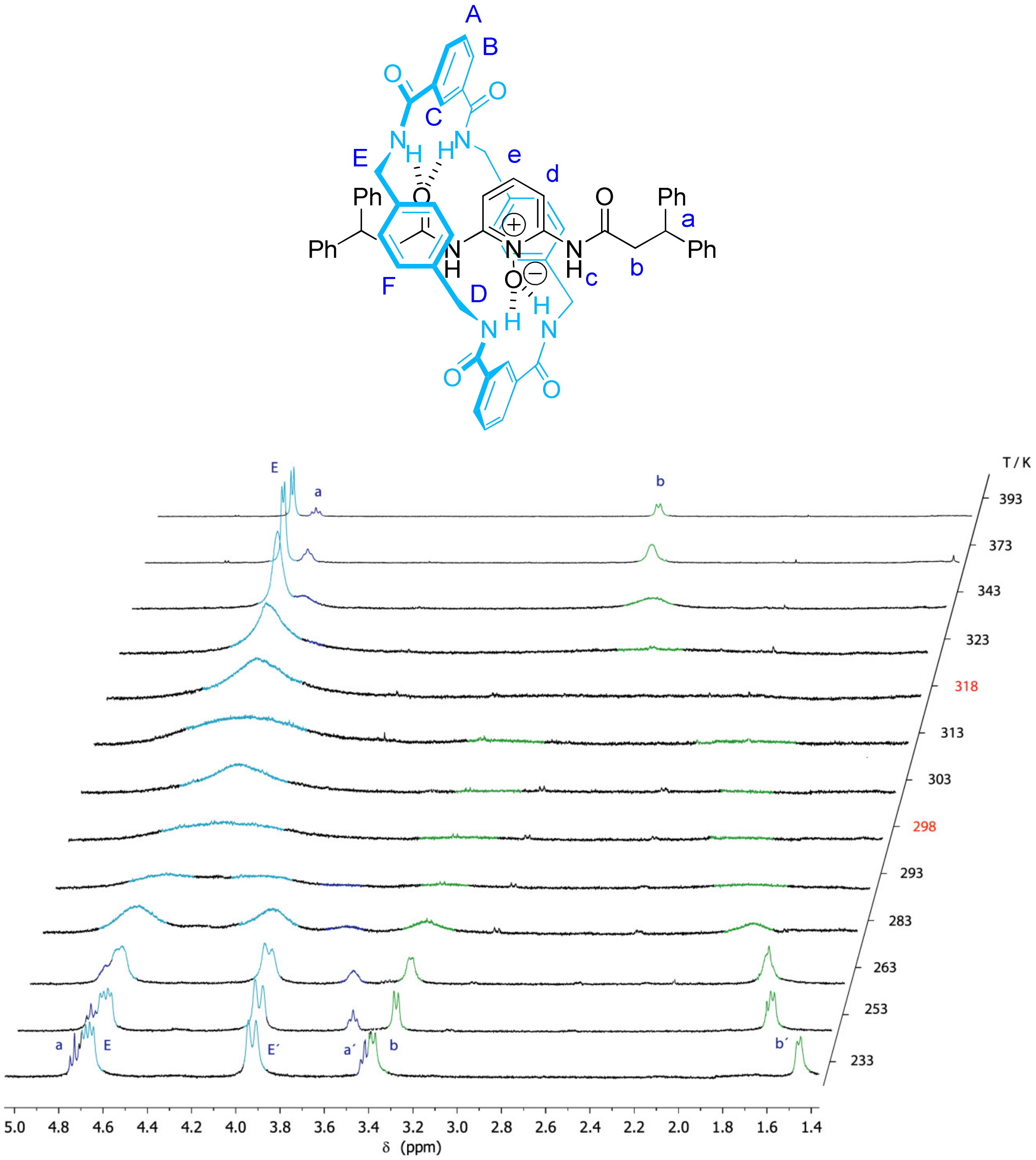

Figure 2. Partial VT-^{1}H NMR spectra (400MHz, $C_2D_2Cl_4$ and CD_2Cl_2) of **2b**.

Similar experiments were carried out for picrate salt **2c**. In this case signals referred to the stoppers H_a and H_b coalesced at the temperature of 238 K, observing a splitting of those signals below that temperature. Interestingly two signals referred to the amide proton NH_c also appeared. At 231 K the signal associated with the methylene protons of the macrocycle (H_E) coalesced, associated with an energy barrier for the macrocycle pirouetting of 10.7 kcal·mol^{-1}.

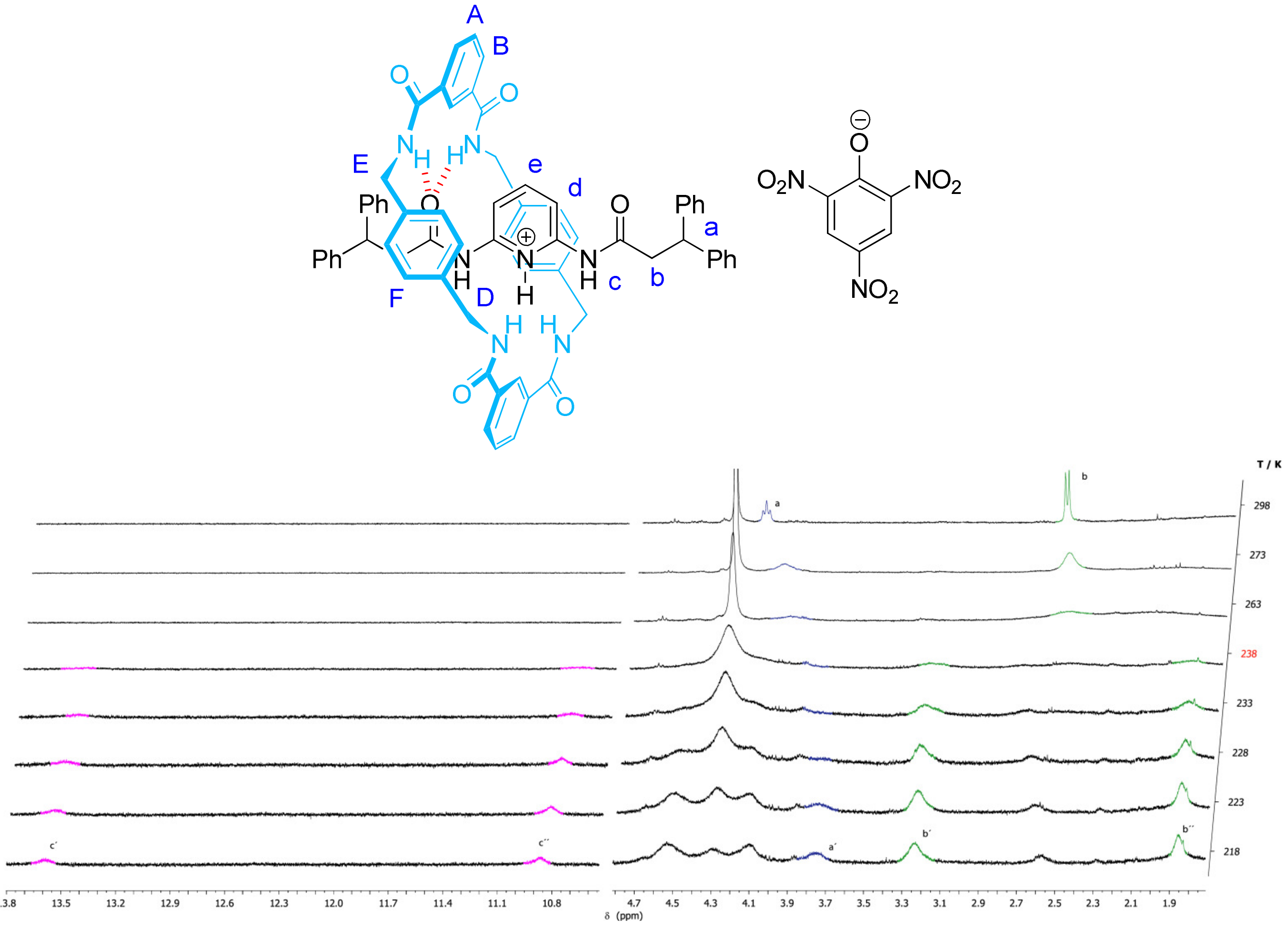

Figure 3. Partial VT-^{1}H NMR spectra (400MHz, CD_2Cl_2) of **2c**.

10. Estimation of the level of occupancy of the DAP unit for rotaxanes 7-8 and 12

In order to estimate the occupancy of the tetraamide macrocycle[3] over the diacylaminopyridine binding site in the synthesized rotaxanes, we focused our attention on the upshifting of the hydrogen atom at 4-position of the pyridine ring (H_e) following the rotaxane formation. These values were compared with the shift of this proton in rotaxanes **2a-c**, where the occupation of the binding site is complete (Table S1). The examination of the chemical shift variations in the ^{1}H NMR of the rotaxanes revealed that the occupation of the DAP station in **7** (68%) is notably higher than in **8** (28%) (Table S1, c .f. entries 4 and 6). In rotaxane **8**, the succinic ester station has stronger affinity for the macrocycle than the DAP moiety. In similar manner, the level of occupancy over the pyridine *N*-oxide function of rotaxane **12** was also calculated, being 100%.

[3] a) A. Altieri, G. Bottari, F. Dehez, D. A. Leigh, J. K. Y. Wong, F. Zerbetto, *Angew. Chem. Int. Ed.* **2003**, *42*, 2296-2300; b) J. Berna, M. Alajarin, R.-A. Orenes, *J. Am. Chem. Soc.* **2010**, *132*, 10741-10747.

Table S1. Level of occupancy of the diacylaminopyridine binding sites of rotaxanes

Entry	Compound	δ H_e (ppm)	Δδ [H_e] (ppm)	% Occupancy
1	**1a**	7.61		
2	**2a**	7.36	-0.25	100
3	**5**	7.65		
4	**7**	7.48	-0.17	68
5	**6**	7.65		
6	**8**	7.58	-0.07	28
7	**1b**	7.22		
8	**2b**	7.35	+0.13	100
9	**13**	7.29		
10	**12**	7.42	+0.13	100
11	**1c**	7.67		
12	**2c**	7.45	-0.22	100

11. Titration experiments of threads and rotaxanes with neutral guests

^{1}H NMR titration spectra were recorded on a Bruker Avance 400 MHz spectrometer, in CD_2Cl_2 at 298 K. Method for the titration with *N*-hexylthymine: A solution of *N*-hexylthymine (40 mM, and 2M in guest) was added to a solution of host (thread or rotaxane) (0.5 mL, 2mM). The chemical shift of a specific host proton was monitored for nineteen titration points (for 0.0-20.0 equivalents of added guest). Two different signals (NH_c and NH_g) have been used to determine the association constant K_{assoc} in the titration of compounds **5** and **7.**

Method for the titration with 1,8-naphthalimide: A solution of rotaxane **7** (40 mM, and 2mM in 1,8-naphthalimimide) was added to a solution of 1,8-naphthalimimide (0.5 mL, 2mM). The chemical shift of the NH proton of the 1,8-Naphthalimimide has been used to determine the association constant K_{assoc} during the titration.

The resulting recordings were analysed using the program HypNMR2008 for curve-fitting NMR titration data.[4] The stoichiometry determination was calculated by plotting the guest mole fraction χ_G *versus* %bound*(1-χ_G), where %bound is $(\delta_{obs}-\delta_0)/(\delta_{max}-\delta_0)$. On the other hand, several separate Job plot experiments (by mixing 2 mM stock solutions of host and guest in varying proportions and acquiring ^{1}H NMR spectra) were also carried out, affording identical stoichiometries to those obtained by the described method.

[4] a) C. Frassineti, S. Ghelli, P. Gans, A. Sabatini, M. S. Moruzzi, A. Vacca, *Anal. Biochem.* **1995**, *231*, 374-382; b) P. Gans, *Protonic Software*, **2008**, "HypNMR" Leeds.

Thread 5 with *N*-hexylthymine

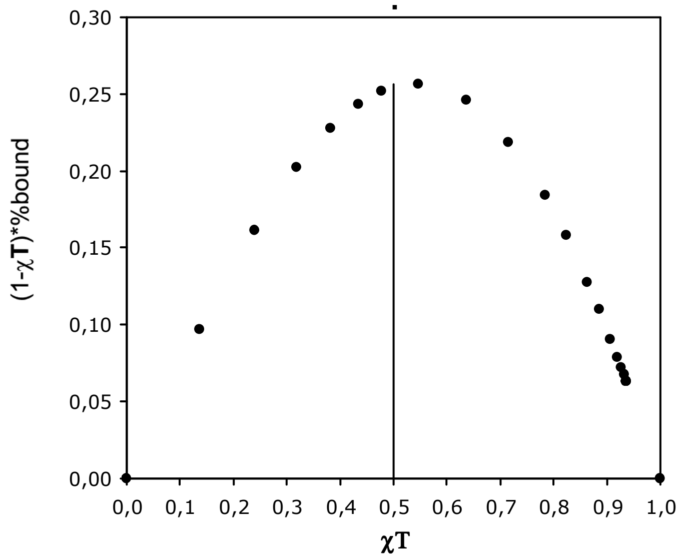

Figure S4a. Job´s plot of thread **5** with *N*-hexylthymine (χ_T vs $(1-\chi_T)$*%bound)

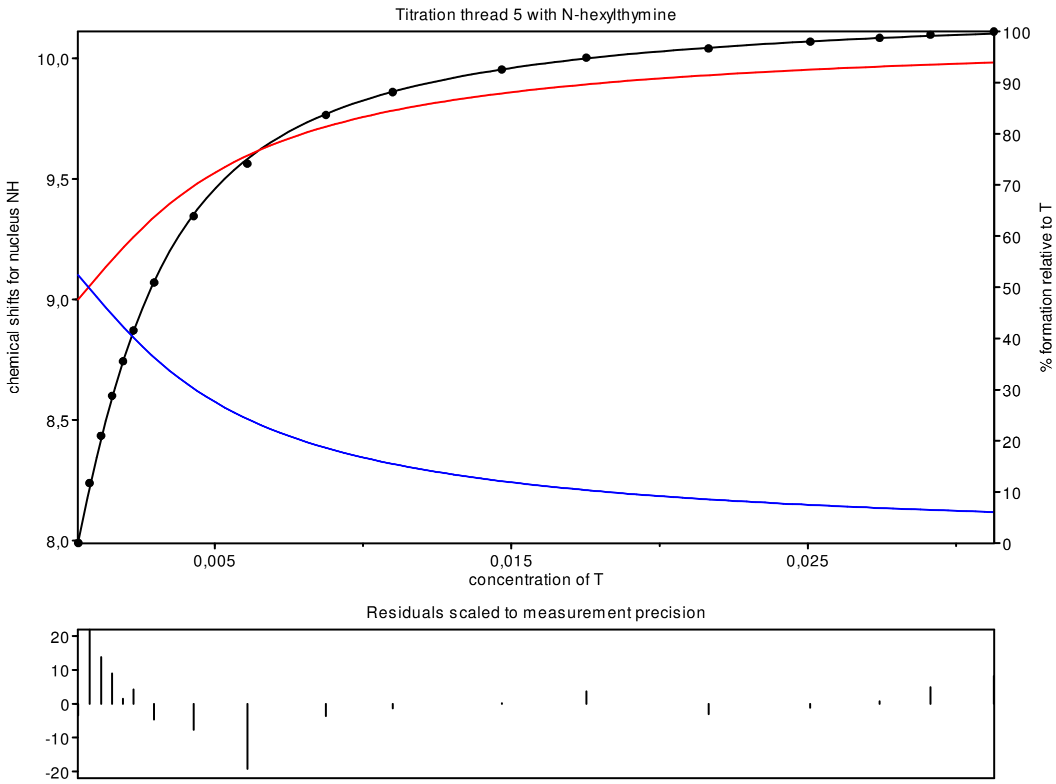

Figure S4b. HypNMR 2008 fitting data for *N*-hexylthymine binding to thread **5** followed by ^{1}H NMR spectra (400 MHz, CD_2Cl_2, 298 K)

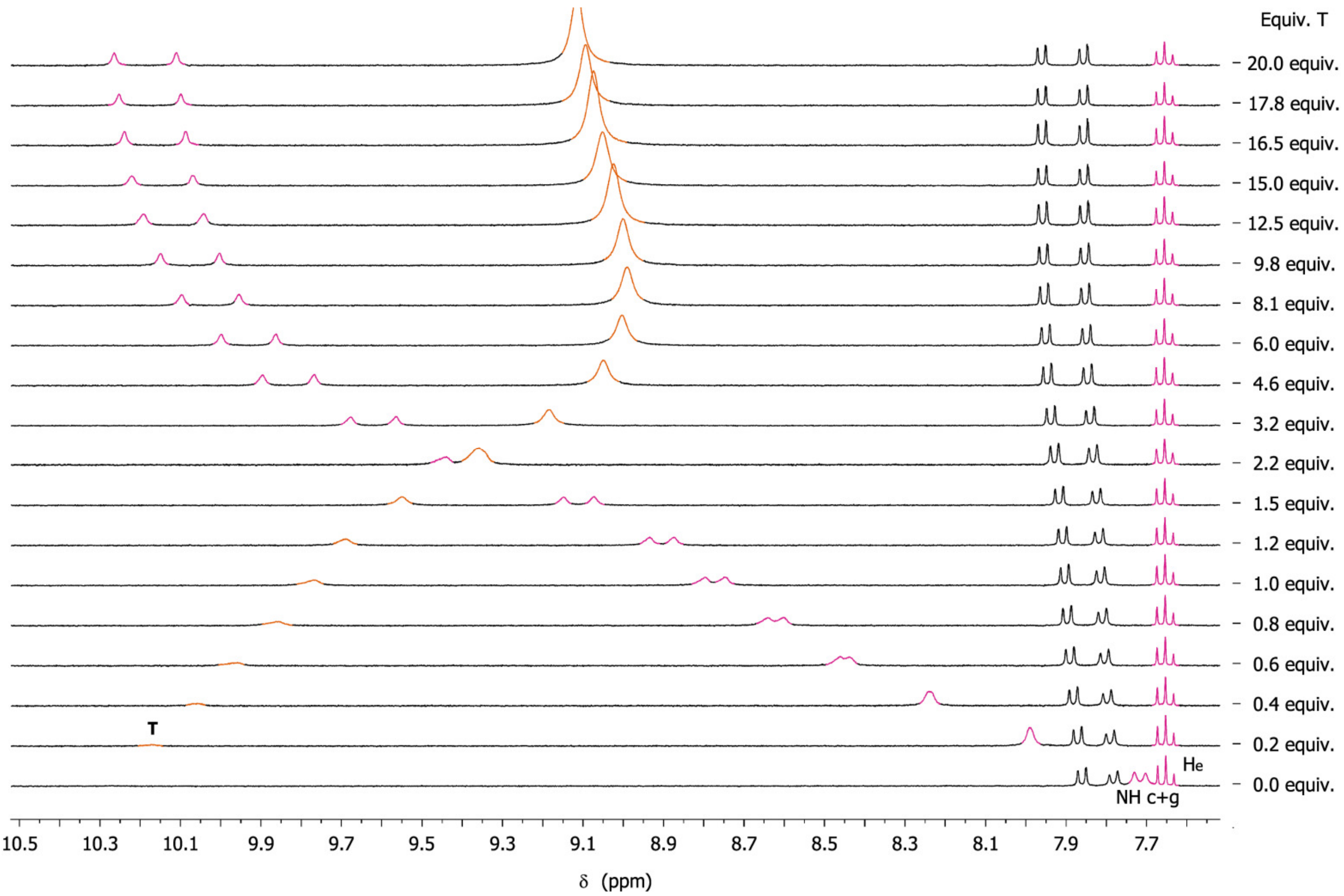

Figure S4c. Partial ^{1}H NMR spectra of titration of thread **5** with *N*-hexylthymine (400 MHz, CD_2Cl_2, 298 K). Chemical shift of amide proton NH_{c+g} (pink) in the presence of *N*-hexylthymine (brown signal)

Rotaxane 7 with *N*-hexylthymine

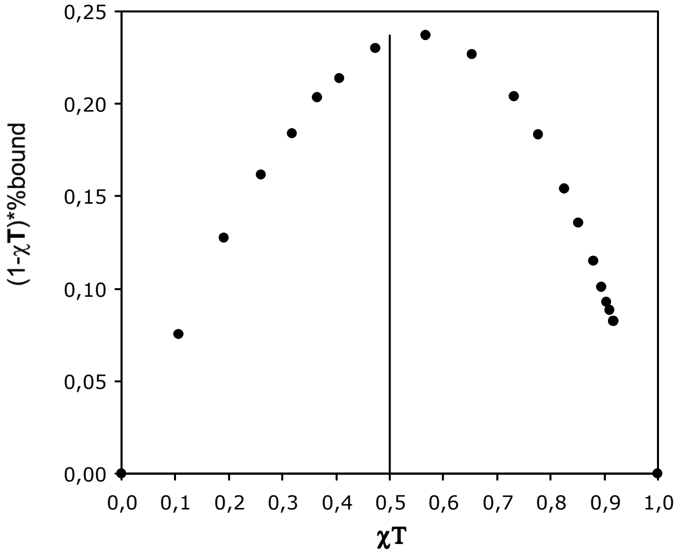

Figure S5a. Job´s plot of rotaxane **7** with *N*-hexylthymine (χ_T vs $(1-\chi_T)$*%bound)

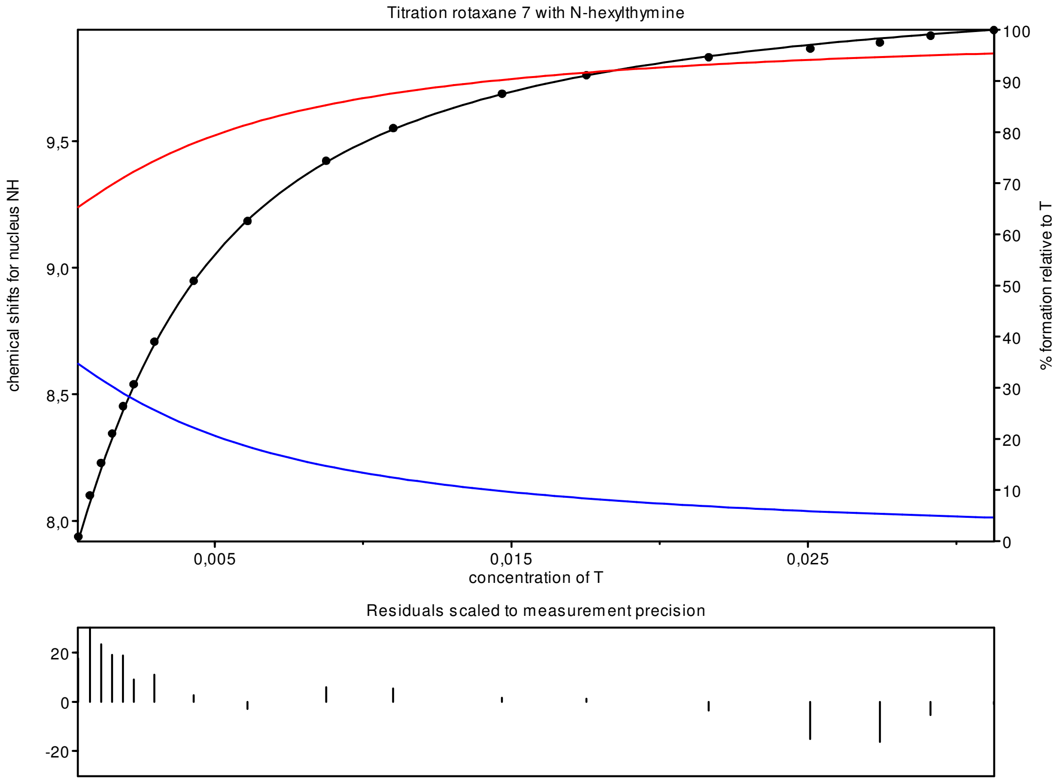

Figure S5b. HypNMR 2008 fitting data for *N*-hexylthymine binding to rotaxane **7** followed by ^{1}H NMR spectra (400 MHz, CD_2Cl_2, 298 K)

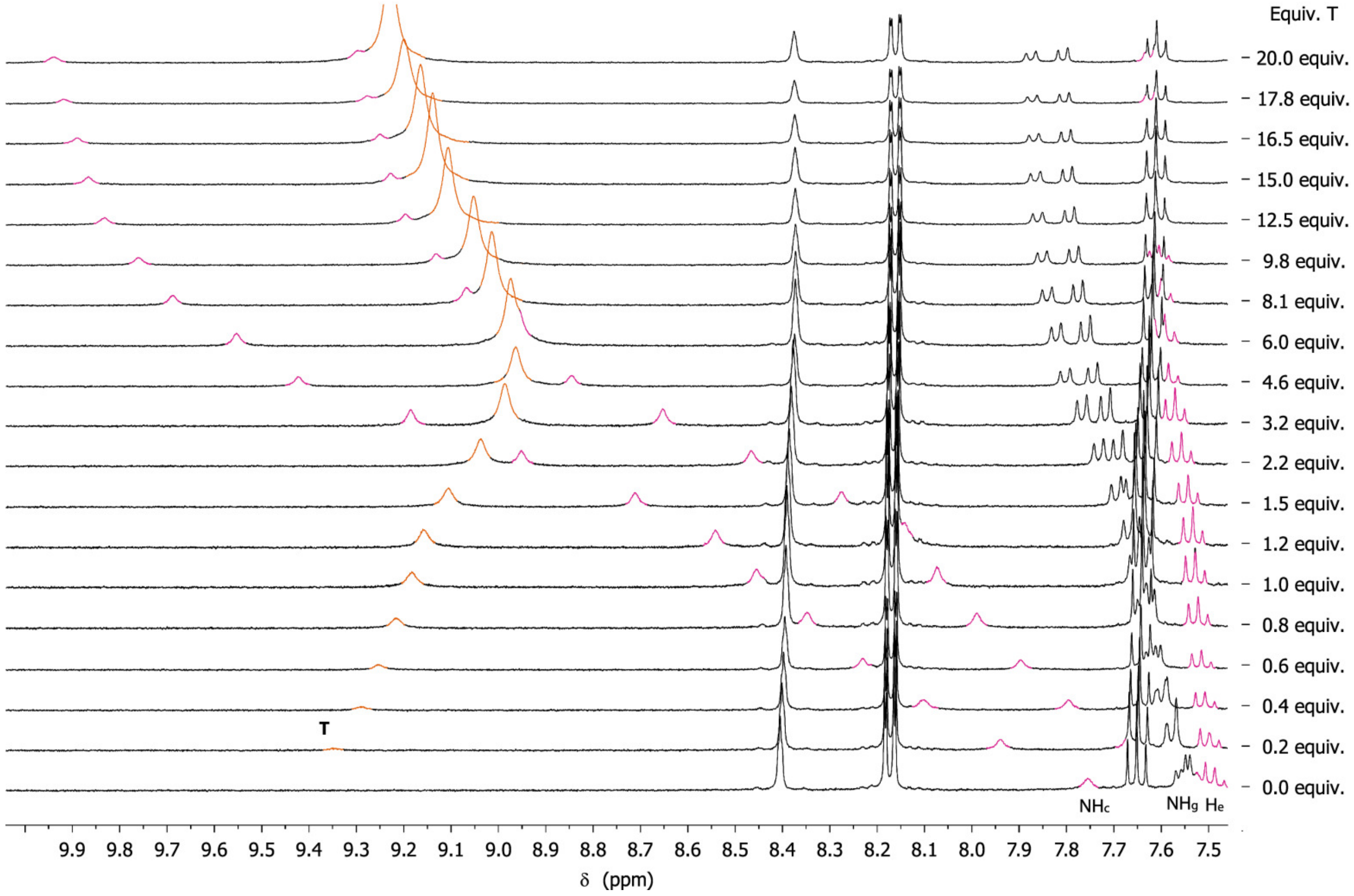

Figure S5c. Partial ^{1}H NMR spectra of titration of rotaxane **7** with *N*-hexylthymine (400 MHz, CD_2Cl_2, 298 K). Chemical shift of amide proton NH_{c+g} (pink) in the presence of *N*-hexylthymine (brown signal)

Rotaxane 7 with 1,8-naphthalimide

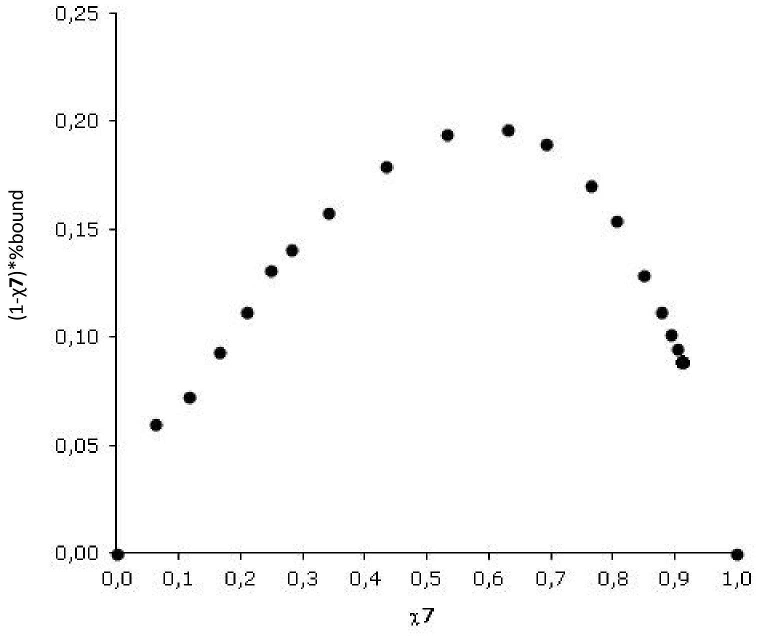

Figure S6a. Job´s plot of rotaxane **7** with 1,8-naphthalimide (χ_7 vs $(1-\chi_7)$*%bound)

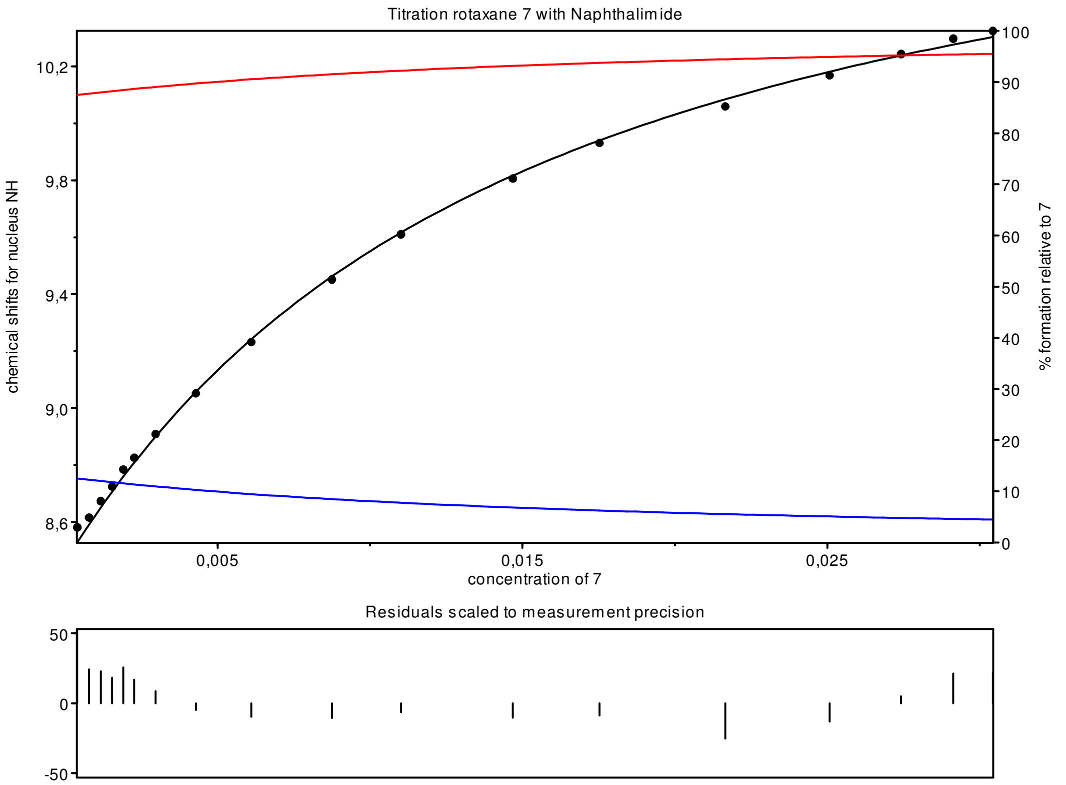

Figure S6b. HypNMR 2008 fitting data for 1,8-naphthalimide binding to rotaxane **7** followed by ^1H NMR spectra (400 MHz, CD_2Cl_2, 298 K)

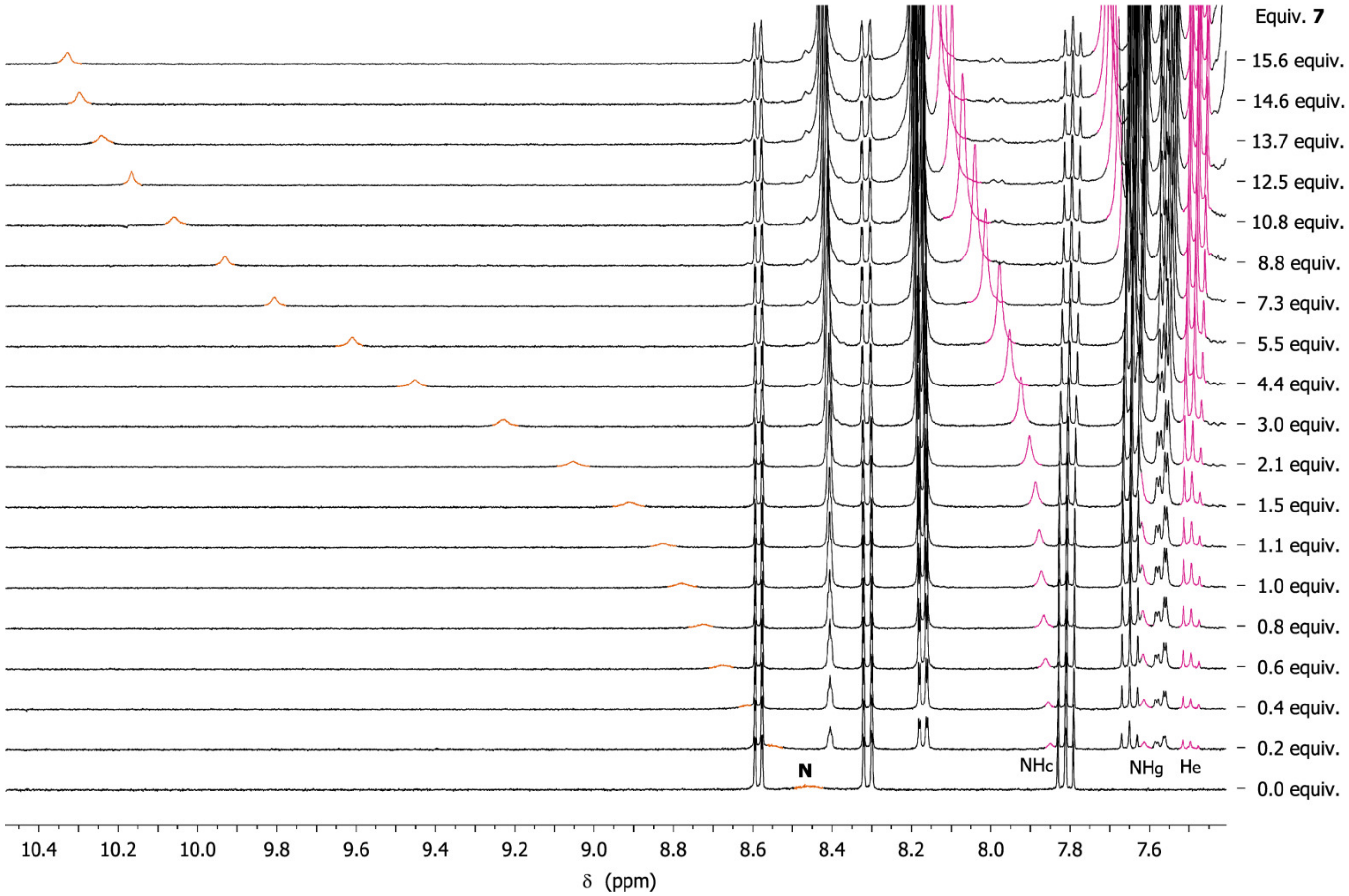

Figure S6c. Partial ^{1}H NMR spectra of titration of 1,8-naphthalimide with rotaxane **7** (400 MHz, CD_2Cl_2, 298 K). Chemical shift of amide proton of the 1,8-naphthalimide (brown signal) in the presence of rotaxane **7** (NH_c, NH_g and H_e pink).

12. Electrochemical Studies.

Electrochemical measurements were performed on a CH Instruments 760D Electrochemical Workstation using CHI Version 10.03 software. Electrochemical experiments for obtaining the cyclic voltammograms (CVs) in the main text were conducted at 298 K using a CH Instruments glassy carbon button working electrode (area = 0.071 cm^2), BASi Ag/$AgNO_3$ pseudo reference electrode, and Pt mesh counter electrode. All electrode potentials were referenced to the ferrocene/ferrocenium couple by doping in samples of ferrocene to the electrolyte. All electrochemical experiments were conducted in electrolyte solutions prepared using HPLC grade DCM, and were thoroughly degassed with argon. The supporting electrolyte was tetrabutylammonium hexafluorophosphate ($TBAPF_6$) at a concentration of 1 mol dm^{-3}. Solutions were agitated between acquisition of individual CVs and CVs were corrected for resistance, using the *iR* compensation function of the potentiostat. All data points were repeated at least twice and gave very similar results. **Figure 5** in the main paper shows representative cyclic voltammograms and the curve shown in **Figure S7** is based on the average $E_{½}$ values obtained from these multiple repeats.

A typical methodology is as follows: To a solution of rotaxane **7** (5 mM) in DCM (with 1 M $TBAPF_6$ as supporting electrolyte), aliquots of a 2.5 mM solution of 1,8-naphthalimide in dry DCM were added. Care was taken to ensure that the overall volume of solvent remained the same in all measurements by allowing added solvents to evaporate in a stream of Ar such that a fixed volume was achieved. All samples were kept under Ar during measurements (CVs were much less reversible under air). CVs were recorded at room temperature and a scan rate of 100 mV/s. Shifts relative to the pseudo reference were converted to shifts vs. ferrocene/ferrocenium by adding ferrocene to the solutions after all other data had been collected.

Rotaxane **7** did not display any redox waves within the window probed (0 to −2 V *vs.* Fc/Fc^+). Within this same region, fully degassed samples of 1,8-naphthalimide displayed a single reversible redox wave with $E_{½}$ = −1.67 V *vs.* Fc/Fc^+ (all references hereafter are quoted *vs.* Fc/Fc^+). Bulk electrolysis to determine the number of electrons associated with this redox wave were conducted in a solution of 1,8-naphthalimide (in 1 M $TBAPF_6$ in dry DCM) using an $Ag/AgNO_3$ pseudo reference electrode, a Pt mesh counter electrode and a large surface area reticulated glassy carbon mesh electrode (BASi) at room temperature (with stirring) and at a potential of −1.9 V. The results indicated that that this redox wave corresponded to a 1-electron redox process.

The shift in the $E_{½}$ value of the 1,8-naphthalimide redox wave was then plotted as a function of the relative amount of rotaxane host compound in solution. A curve of the average shift in these values is shown in **Figure S7** (where 0 equivalents of **7** corresponds to the reduction wave for 1,8-naphthalimide on its own). This shows that maximal shift is achieved in the presence of around 6 equivalents of rotaxane, and that the presence of further equivalents of rotaxane makes little difference to the shift.

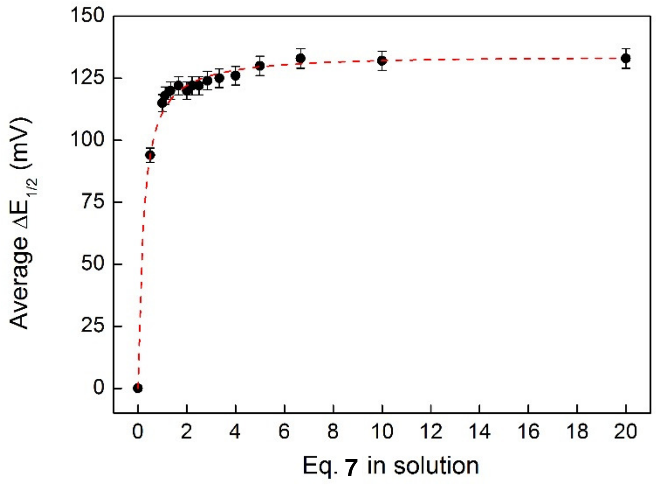

Figure S7. Comparison of the shift in $E_{½}$ value for the 1,8-naphthalimide redox wave in the presence of various equivalents of rotaxane **7**. These values are averages of at least two experiments for each point. Error bars are based on standard deviations and the red dashed line is provided simply as a guide to the eye.

13. Conformational study by NMR spectroscopy for rotaxane 7, picrate salt 14 and complex 7:T.

Rotaxane 7

The occupancy of the tetraamide ring over the di(acylamino)pyridine site takes a value of 68%. It was determined on the basis of the upfield shift of the ^{1}H NMR signal of the proton at the 4-position of the pyridine ring (H_e).

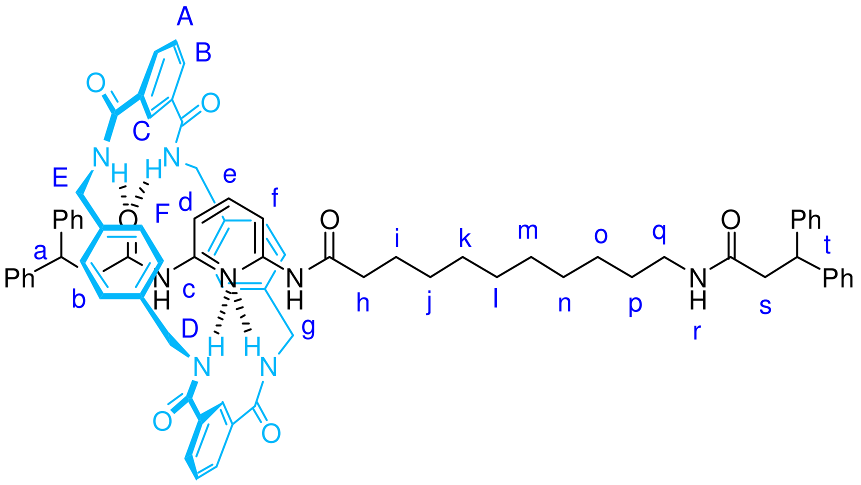

All resonances except those for protons l-o, placed at the aliphatic chain, could be assigned on the basis of the COSY and ^{1}H,^{1}H-NOESY spectra. The ^{1}H,^{1}H-NOESY spectrum shows crosspeaks relating protons from the ring and those of the diacylaminopyridine site compatible with the estimated occupation percentage. Thus, we found crosspeaks relating protons H_C-H_c, H_C-H_g, H_F-$H_{d/f}$ and H_F-H_e in the aromatic region of the spectrum (**Figure S8**). The spectrum also shows crosspeaks relating protons H_F-H_c and H_F-H_g.

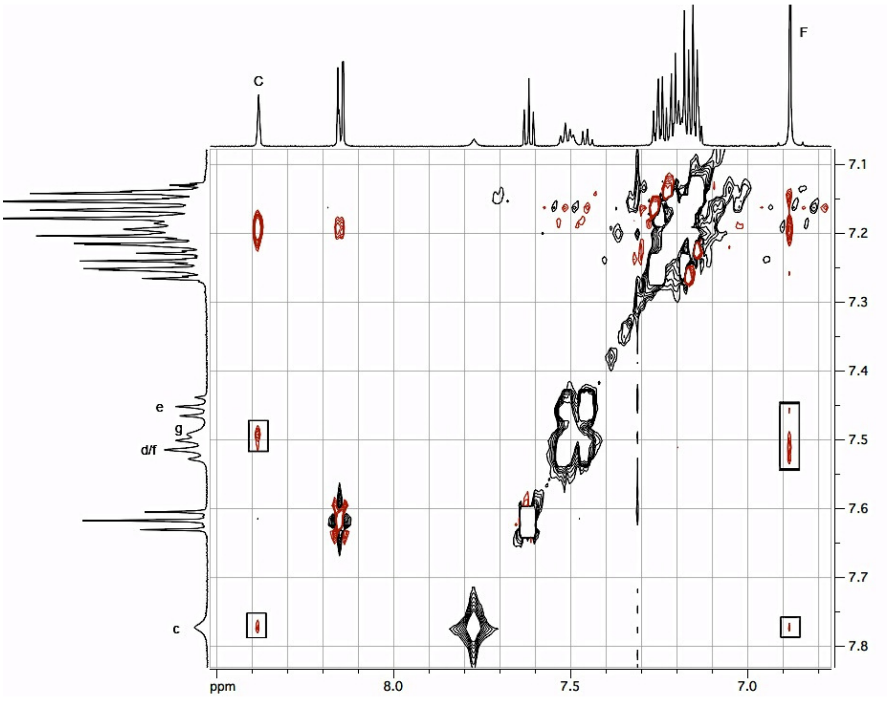

Figure S8. Partial ^{1}H,^{1}H-NOESY spectrum crosspeaks for rotaxane **7**

Additionally, the NOESY spectrum reveals the proximity between the aromatic protons H_F placed at the macrocycle and several aliphatic protons close to the diacylaminopyridine station (H_b, H_h, H_i, H_j and H_k) (**Figures S9** and **S10**). **Weak** crosspeaks relating protons H_F-H_s and H_q support the 32% of occupancy over the amide function.

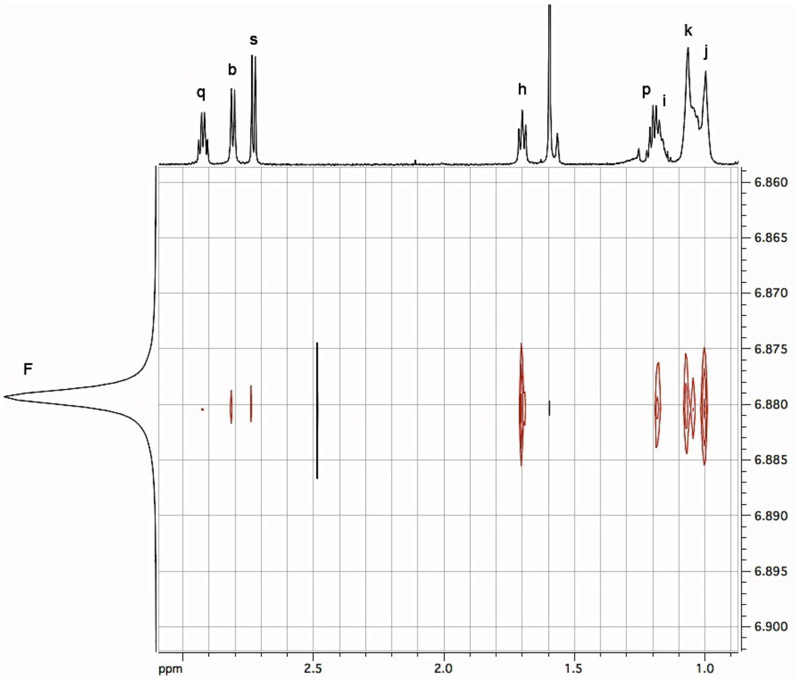

Figure S9. Partial ^{1}H,^{1}H-NOESY spectrum crosspeaks for rotaxane **7**

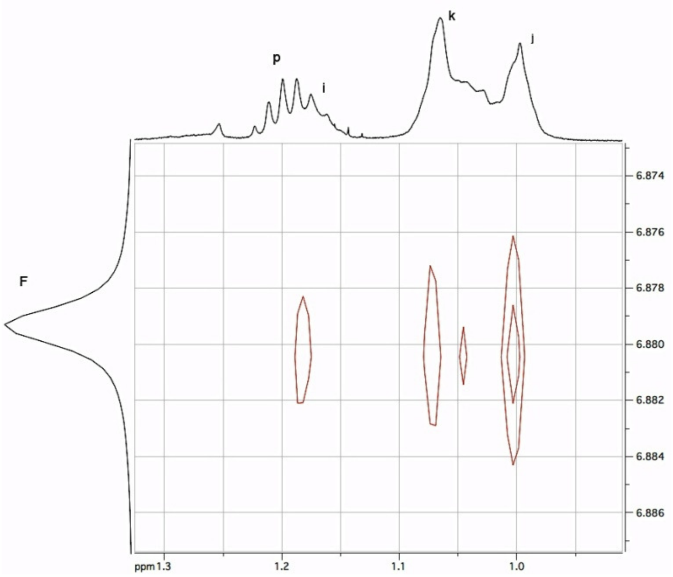

Figure S10. Partial ^{1}H,^{1}H-NOESY spectrum crosspeaks for rotaxane **7**

Picrate salt 14

The protonation of the nitrogen atom at the pyridine ring of rotaxane **7** causes large displacements of the NMR resonances of protons H_d and H_f ($\Delta\delta$ = - 0.10 and - 0.24 ppm), which appear at lower frequencies in spite of the proximity of the positive charge. These displacements to lower frequencies support the conformational change of the diacylaminopyridine station that would keep the oxygen atoms of the

carbonylic groups far apart from these protons. The conformational change experienced by the diacylaminopyridine station induces the translation of the macrocycle along the aliphatic chain to the amide site. The shift to higher frequency observed for H_b ($\Delta\delta$ = + 0.35 ppm) is in agreement with this fact as well as the shifts to lower frequencies underwent by selected protons of the aliphatic chain and H_s ($\Delta\delta$ = - 0.10 ppm). All resonances, except those for protons $H_{m\text{-}n}$ placed at the aliphatic chain, could be assigned on the basis of the COSY and ^{1}H,^{1}H-NOESY spectra**.**

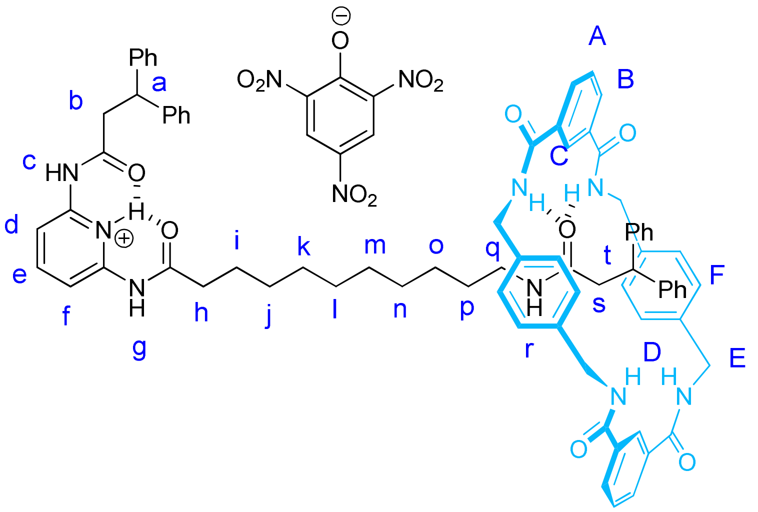

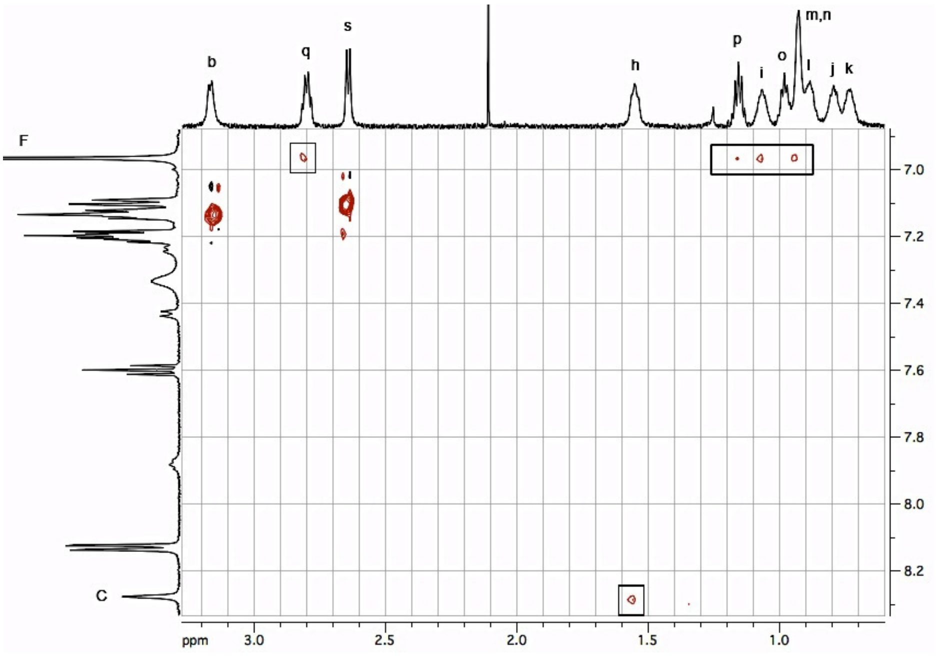

Figure S11. Partial ^{1}H,^{1}H-NOESY spectrum crosspeaks for rotaxane **14**

The ^{1}H,^{1}H-NOESY spectrum does not show crosspeaks relating protons from the macrocycle and those of the diacylaminopyridine station (**Figure S11**). However, the spectrum shows intense crosspeaks between H_F-H_q, H_F-$H_{m,n}$, H_F-H_i and H_C-H_h compatible with translocation of the macrocycle. Also weak crosspeaks relating protons H_F-H_p and H_F-H_j have been found (**Figure S12**).

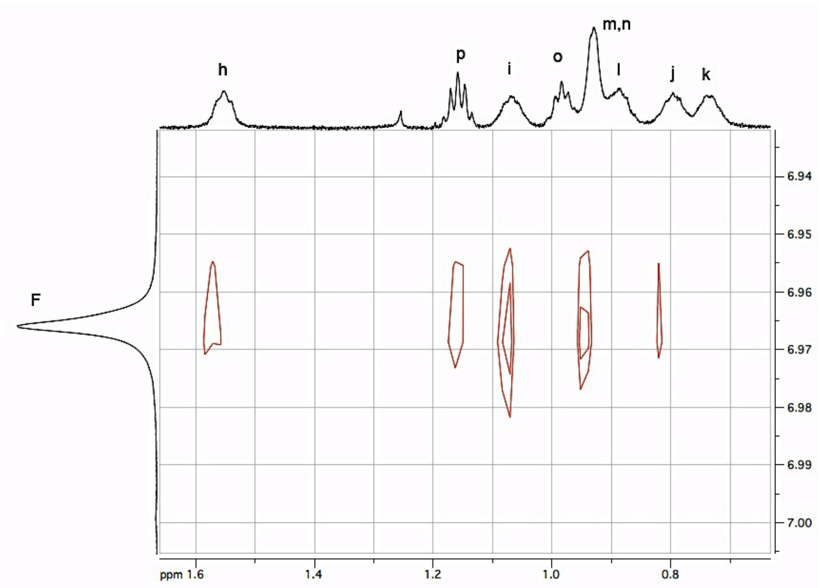

Figure S12. Partial ^{1}H,^{1}H-NOESY spectrum crosspeaks for rotaxane **14**

Rotaxane 7 in the presence of 4 equivalents of *N*-hexylthymine

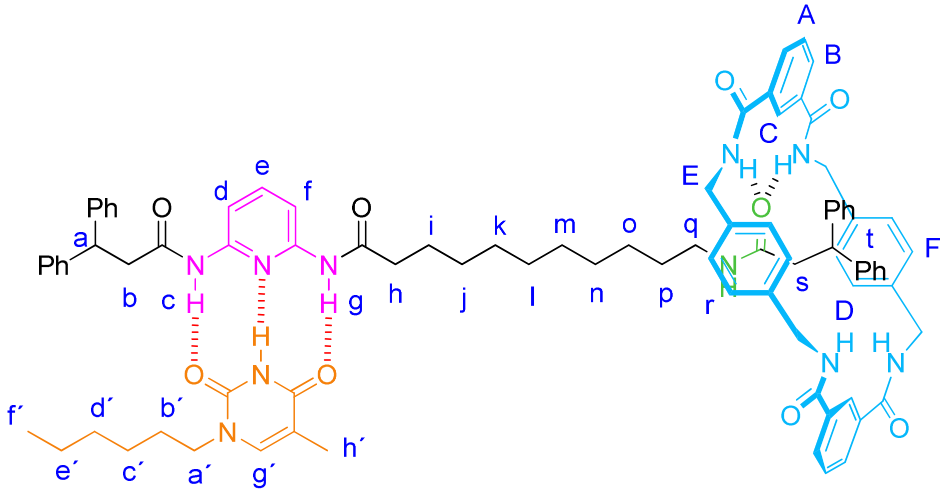

The addition of *N*-hexylthymine (4 equiv.) causes dramatic changes in the chemical shifts of nearly all resonances of the shuttle **7**. Protons H_d, H_e and H_f, placed at the pyridine ring, appear at higher frequencies (Δδ between + 0.11 and + 0.22 ppm) indicating that the shielding effect of the tetraamide ring has faded away. For the same reason, the resonance of H_b moves to higher frequency (Δδ = + 0.34 ppm). On the contrary, the signal for proton H_s appears upfield (Δδ = - 0.16 ppm) as well as all resonances for the aliphatic chain. All these data agree well with a decrease in the occupancy percentage of the macrocycle at the diacylaminopyridine site, due to the association of this moiety to *N*-hexylthymine. In fact, the ratio of occupancy determined as usual takes a value of 32:68.

As expected, the $^{1}H,^{1}H$-NOESY spectrum does not show crosspeaks relating protons from the macrocycle and those of the diacylaminopyridine station (all resonances, except those for protons l-o placed at the aliphatic chain, could be assigned on the basis of the COSY and $^{1}H,^{1}H$-NOESY spectra). On the contrary, the spectrum shows intense crosspeaks relating protons of the macrocycle and those close to the amide site, i.e. H_C-H_r, H_C-H_s, H_F-H_q and H_F-H_s (**Figure S13**). The NOESY spectrum also displays a crosspeak between H_C and H_h. Moreover, strong signals are observed between protons H_F and those of the aliphatic chain $H_{i\text{-}p}$ (**Figure S14**). Finally, the NOESY spectrum displays also weak crosspeaks between H_D-H_q and H_D-H_s (**Figure S15**).

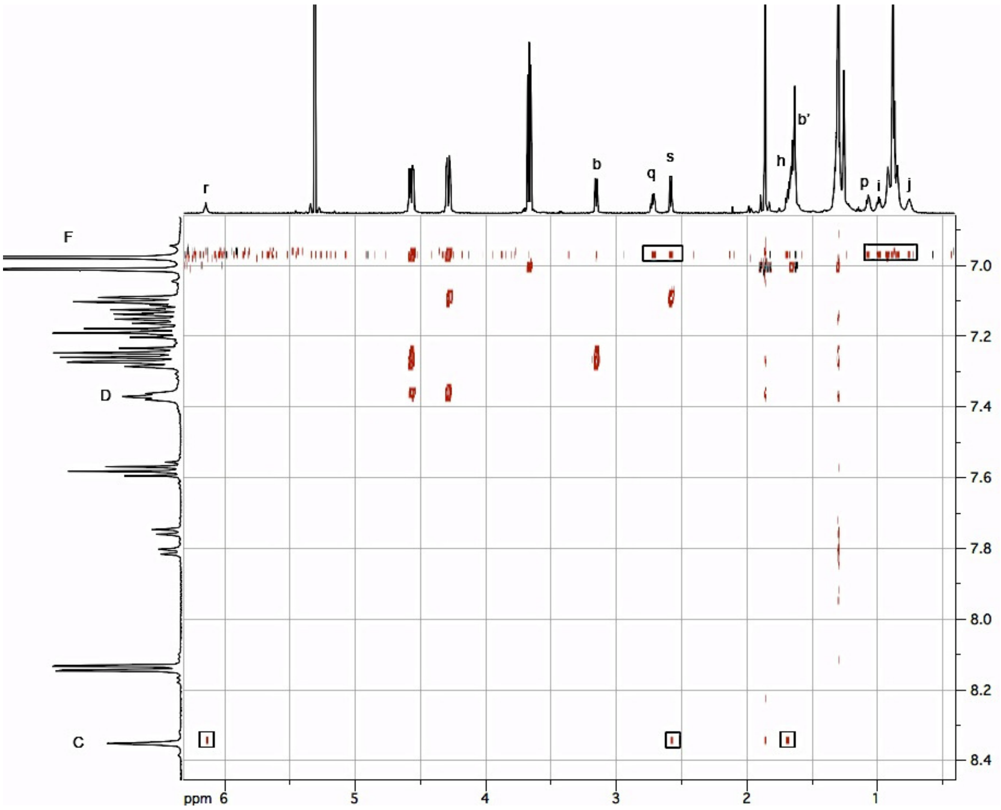

Figure S13. Partial $^{1}H,^{1}H$-NOESY spectrum crosspeaks for rotaxane **7** in the presence of *N*-hexylthymine

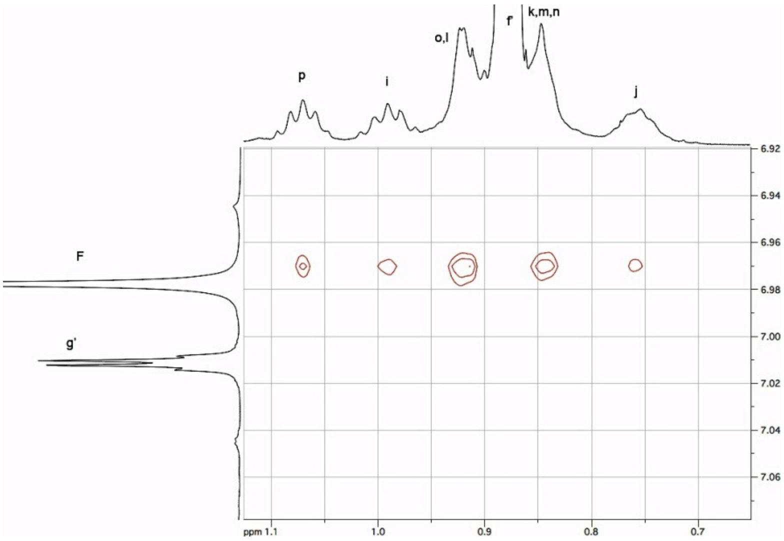

Figure S14. Partial $^{1}H,^{1}H$-NOESY spectrum crosspeaks for rotaxane **7** in the presence of *N*-hexylthymine

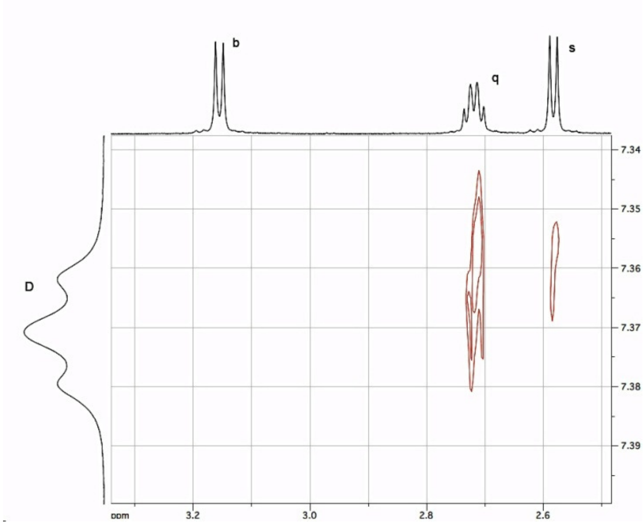

Figure S15. Partial $^{1}H,^{1}H$-NOESY spectrum crosspeaks for rotaxane **7** in the presence of *N*-hexylthymine

14. Crystal data and structure refinement for 1c and 2b

Picrate salt 1c (CCDC 1051908)

A yellow prism-like specimen of $C_{42}H_{34}N_6O_{10}$, approximate dimensions 0.0400 mm x 0.160 mm x 0.300 mm, was used for the X-ray crystallographic analysis. The X-ray intensity data were measured on a Bruker D8 QUEST system equipped with a multilayer monochromator and a Cu K/a Incoatec microfocus sealed tube (λ = 1.54178 Å).

A total of 2331 frames were collected. The total exposure time was 25.90 hours. The frames were integrated with the Bruker SAINT software package using a narrow-frame algorithm. The integration of the data using a triclinic unit cell yielded a total of 110777 reflections to a maximum θ angle of 67.29° (0.84 Å resolution), of which 13311 were independent (average redundancy 8.322, completeness = 98.7%, R_{int} = 7.51%, R_{sig} = 3.74%) and 11007 (82.69%) were greater than $2\sigma(F^2)$. The final cell constants of a = 8.8838(4) Å, b = 20.6864(10) Å, c = 22.5136(11) Å, α = 68.276(3)°, β = 78.717(3)°, γ = 89.509(3)°, volume = 3760.0(3)Å^3, are based upon the refinement of the XYZ-centroids of 9562 reflections above 20 σ(I) with 8.592° < 2θ < 133.8°. Data were corrected for absorption effects using the multi-scan method (SADABS). The ratio of minimum to maximum apparent transmission was 0.820. The calculated minimum and maximum transmission coefficients (based on crystal size) are 0.7872 and 0.9673.

The structure was solved and refined using the Bruker SHELXTL Software Package, using the space group P -1, with Z = 4 for the formula unit, $C_{42}H_{34}N_6O_{10}$. The final anisotropic full-matrix least-squares refinement on F^2 with 1063 variables converged at R1 = 11.87%, for the observed data and wR2 = 27.48% for all data. The goodness-of-fit was 1.173. The largest peak in the final difference electron density synthesis was 0.827 e^-/Å^3 and the largest hole was -0.737 e^-/Å^3 with an RMS deviation of 0.105 e^-

/Å^3. On the basis of the final model, the calculated density was 1.383 g/cm^3 and F(000), 1632 e$^-$.The poor overall precision resulted from the presence of a great deal of disordered picrate anions and methanol co-solvate molecules, coupled with poor overall crystal quality as a result of decomposition.

Table S2. Crystal data and structure refinement for **1c**

	**1c**
Empirical formula	$C_{42} H_{34} N_6 O_{10}$
Formula weight	782.75
T [K]	100(2)
Wavelength [Å]	1.54178
Crystal system	Triclinic
Space group	P-1
a (Å)	8.8838(4)
b (Å)	20.6864(10)
c (Å)	22.5136(11)
α (°)	68.276(3)
β (°)	78.717(3)
γ (°)	89.509(3)
V [Å^3]	3760.0(3)
Z	4
ρ [g·cm^{-3}]	1.383
μ [mm^{-1}]	0.838
F_{000}	1632
Crystal size [mm^3]	0.30 x 0.16 x 0.04
θ range (°)	2.50-67.29
h	-10 to 10
k	-24 to 24
l	-26 to 26
Reflections collected	110777
Independent reflections	13311
R(int)	0.0751
Refinement method	
Parameters	1063
Restraints	0
Goodness-of-fit on F^2	1.152
*R*1 [I > 2σ(I)]	0.1173
*wR*2 [I > 2σ(I)]	0.2660
*R*1 (all data)	0.1330
*wR*2 (all data)	0.2734
$\Delta\rho$ [e·Å^{-3}]	0.827/-0.737

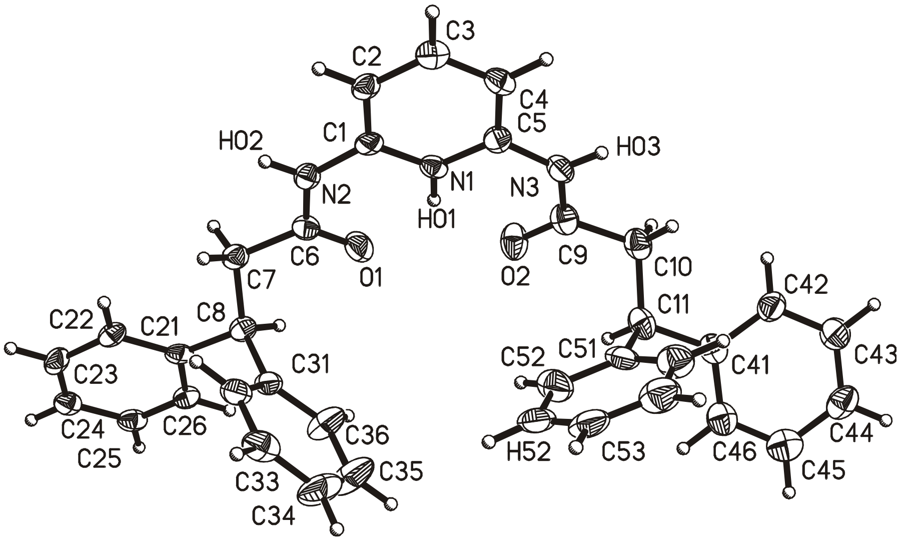

Figure S16. Molecular structure of salt **1c** (CCDC 1051908) with thermal ellipsoids drawn at 50% probability. For clarity, the picrate anion and a methanol co-solvate molecule have been omitted.

Table S3. Hydrogen bonds for rotaxane **1c** [Å and (°)].

D-H...A	d(D-H)	d(H...A)	d(D...A)	<(DHA)
N(1)-H(01)...O(1)	0.880	1.930	2.595	131.17
N(1)-H(01)...O(2)	0.880	2.034	2.682	129.59

Rotaxane 2b (CCDC 1051909)

A colorless prism-like specimen of $C_{67}H_{57.75}N_7O_7$, approximate dimensions 0.050 mm x 0.060 mm x 0.250 mm, was used for the X-ray crystallographic analysis. The X-ray intensity data were measured on a Bruker D8 QUEST system equipped with a multilayer monochromator and a Cu K/a Incoatec microfocus sealed tube (λ = 1.54178 Å).

A total of 4232 frames were collected. The total exposure time was 70.53 hours. The frames were integrated with the Bruker SAINT software package using a narrow-frame algorithm. The integration of the data using a triclinic unit cell yielded a total of 75241 reflections to a maximum θ angle of 66.78° (0.84 Å resolution), of which 9349 were independent (average redundancy 8.048, completeness = 99.5%, R_{int} = 4.94%, R_{sig} = 2.58%) and 8146 (87.13%) were greater than 2σ(F^2). The final cell constants of a = 10.4583(9) Å, b = 13.5669(12) Å, c = 19.9470(17) Å, α = 76.564(4)°, β = 75.557(4)°, γ = 80.757(4)°, volume = 2649.9(4) $Å^3$, are based upon the refinement of the XYZ-centroids of 9520 reflections above 20 σ(I) with 6.738° < 2θ < 133.5°. Data were corrected for absorption effects using the multi-scan method (SADABS). The ratio of minimum to maximum apparent transmission was 0.868. The calculated minimum and maximum transmission coefficients (based on crystal size) are 0.8426 and 0.9654.

The structure was solved and refined using the Bruker SHELXTL Software Package, using the space group P -1, with Z = 2 for the formula unit, $C_{67}H_{57.75}N_7O_7$. The final anisotropic full-matrix least-squares refinement on F^2 with 773 variables converged at R1 = 5.68%, for the observed data and wR2 = 13.16%

for all data. The goodness-of-fit was 1.104. The largest peak in the final difference electron density synthesis was 0.715 $e^-/Å^3$ and the largest hole was -0.504 $e^-/Å^3$ with an RMS deviation of 0.06 $e^-/Å^3$. On the basis of the final model, the calculated density was 1.345 g/cm^3 and F(000), 1130 e^-.

Table S4. Crystal data and structure refinement for **2b**

	**2b**
Empirical formula	$C_{67}H_{57.75}N_7O_7$
Formula weight	1072.95
T [K]	100(2)
Wavelength [Å]	1.54178
Crystal system	Triclinic
Space group	P -1
a (Å)	10.4583(9)
b (Å)	13.5669(12)
c (Å)	19.9470(17)
α (°)	76.564(4)
β (°)	75.557(4)
γ (°)	80.757(4)
V [$Å^3$]	2649.9(4)
Z	2
ρ [$g·cm^{-3}$]	1.345
μ [mm^{-1}]	0.709
F_{000}	1130
Crystal size [mm^3]	0.25 x 0.06 x 0.05
θ range (º)	3.37-66.78
h	-12 to 12
k	-16 to 16
l	-23 to 23
Reflections collected	75241
Independent reflections	9349
R(int)	0.0494
Refinement method	
Parameters	773
Restraints	0
Goodness-of-fit on F^2	1.104
*R*1 [I > 2σ(I)]	0.0568
*wR*2 [I > 2σ(I)]	0.1263
*R*1 (all data)	0.0662
*wR*2 (all data)	0.1316
$\Delta\rho$ [$e·Å^{-3}$]	0.715/-0.504

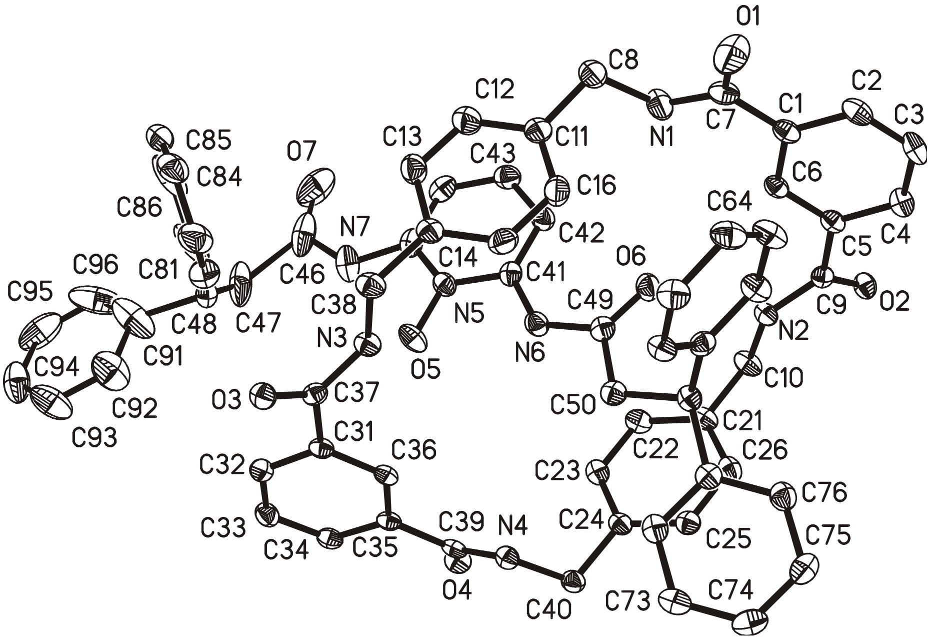

Figure S17. Molecular structure of rotaxane **2b** (CCDC 1051909) with thermal ellipsoids drawn at 50% probability.

Table S5. Hydrogen bonds for rotaxane **2b** [Å and (°)].

D-H...A	d(D-H)	d(H...A)	d(D...A)	<(DHA)
N(2)-H(02)...O(6)	0.86(3)	2.07(3)	2.922(2)	171(2)
N(3)-H(03)...O(5)	0.88(3)	2.00(3)	2.843(2)	162(2)
N(6)-H(06)...O(5)	0.90(3)	2.01(3)	2.526(2)	115(2)
N(7)-H(07)...O(5)	0.88(3)	2.04(3)	2.531(3)	114(2)
N(1)-H(01)...O(3)#1	0.96(3)	2.08(3)	3.010(3)	161(3)
N(4)-H(04)...O(2)#2	0.88(3)	2.09(3)	2.966(2)	174(2)
C(84)-H(84)...O(1)#3	0.95	2.58	3.242(3)	127.2
C(72)-H(72)...O(2)#2	0.95	2.44	3.237(3)	141.4
C(6)-H(6)...O(3)#1	0.95	2.53	3.117(3)	120.6
C(42)-H(42)...O(3)#1	0.95	2.38	3.131(3)	135.9
C(6)-H(6)...O(6)	0.95	2.36	3.123(3)	136.8

Symmetry transformations used to generate equivalent atoms: #1 x-1,y,z #2 x+1,y,z #3 -x+1,-y,-z+2

15. ^{1}H and ^{13}C NMR spectra of synthesized compounds

4 (^{1}H NMR, 400 MHz, $CDCl_3$, 298 K)

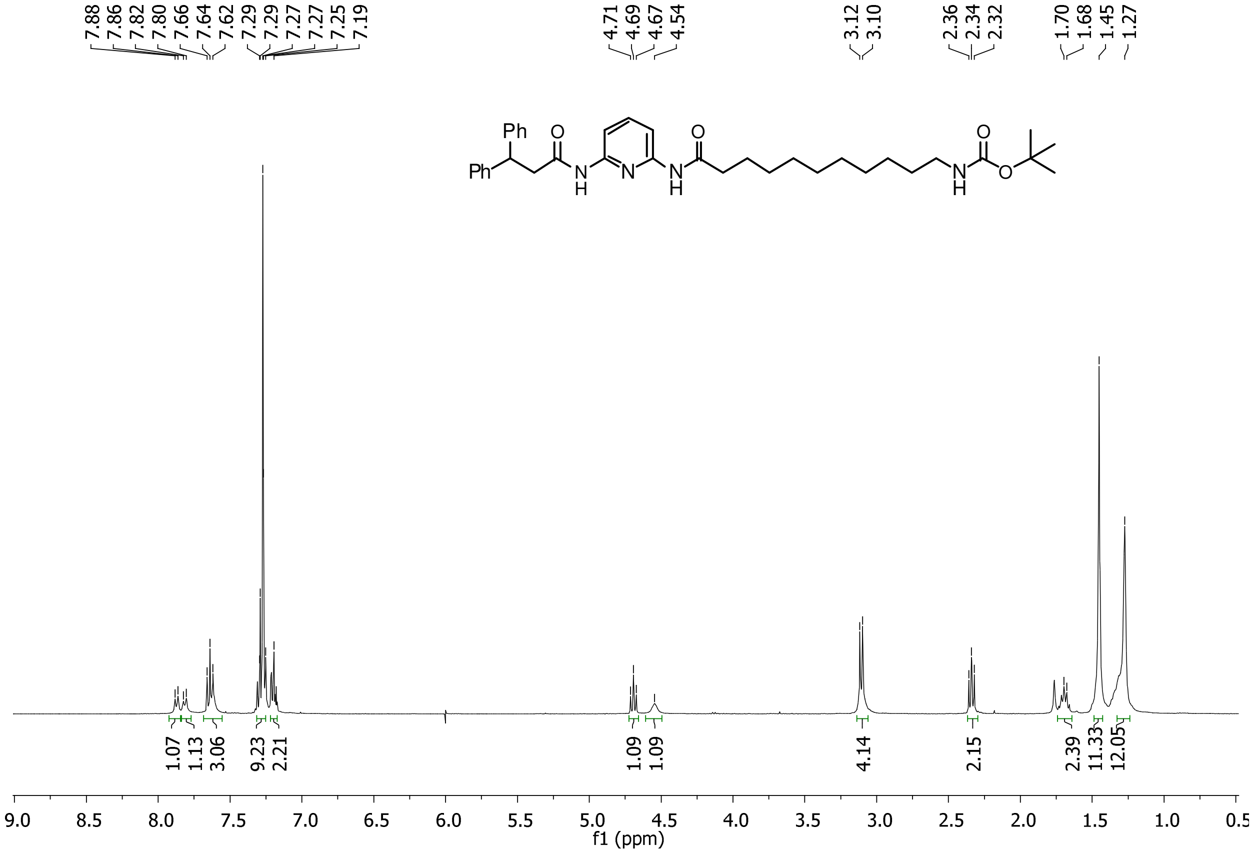

4 (^{13}C NMR, 100 MHz, $CDCl_3$, 298 K)

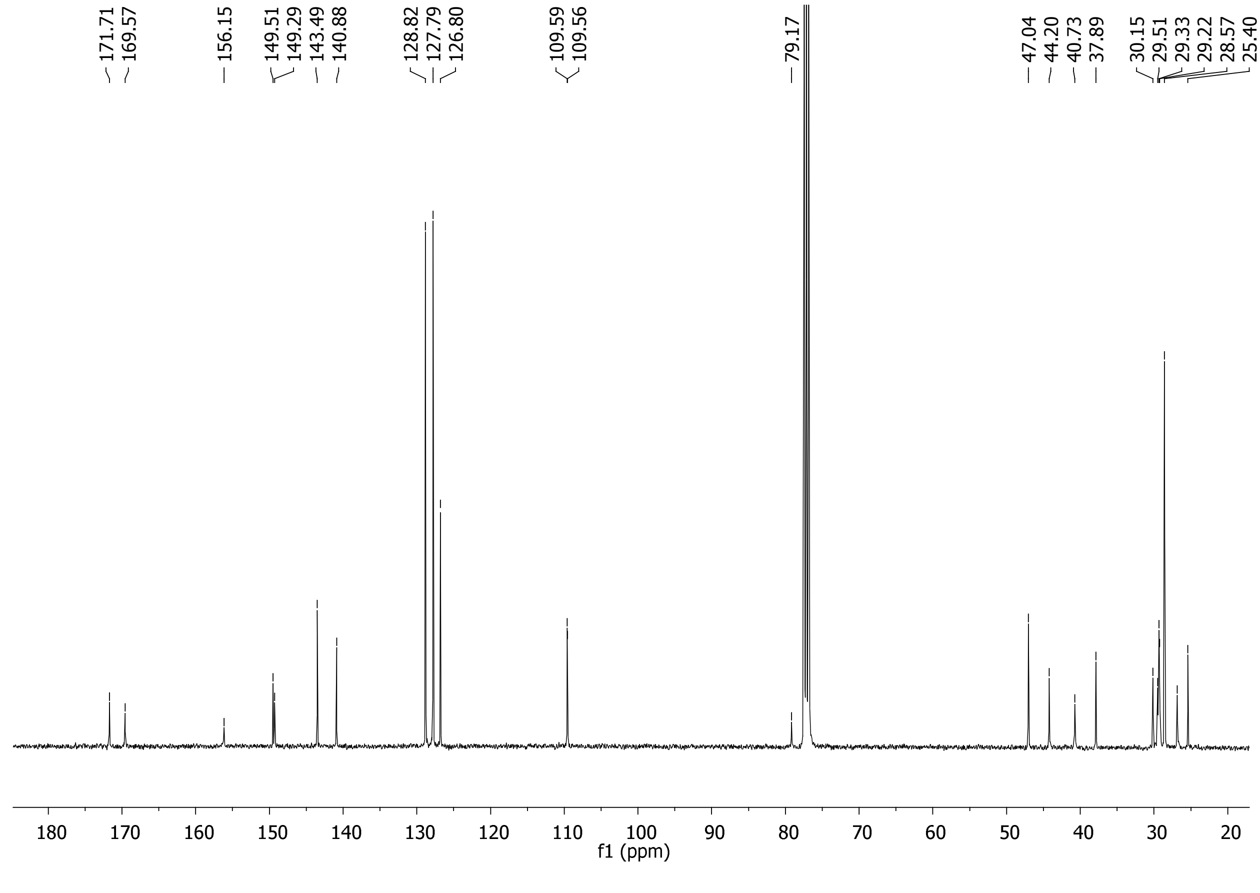

5 (^{1}H NMR, 300 MHz, CD_2Cl_2, 298 K)

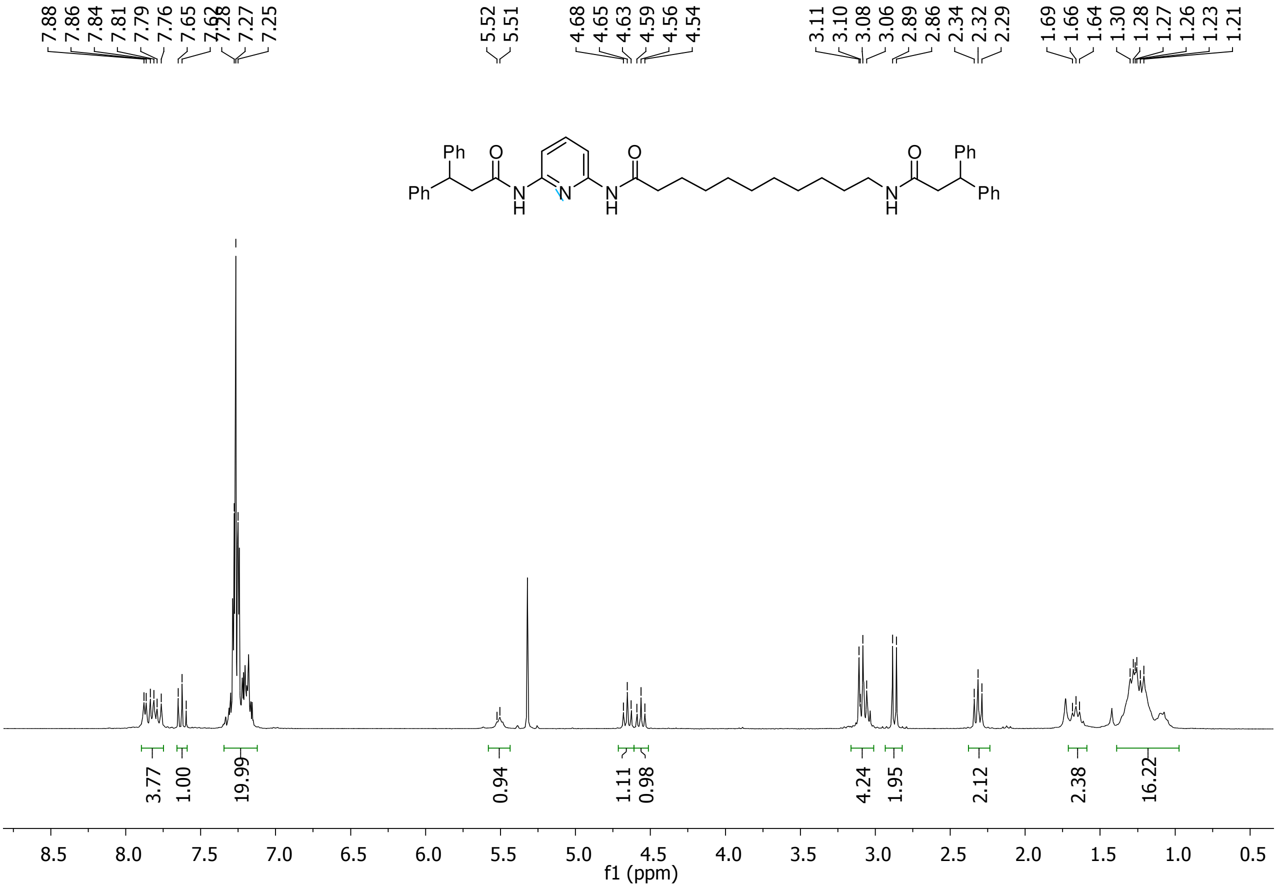

5 (^{13}C NMR, 75 MHz, CD_2Cl_2, 298 K)

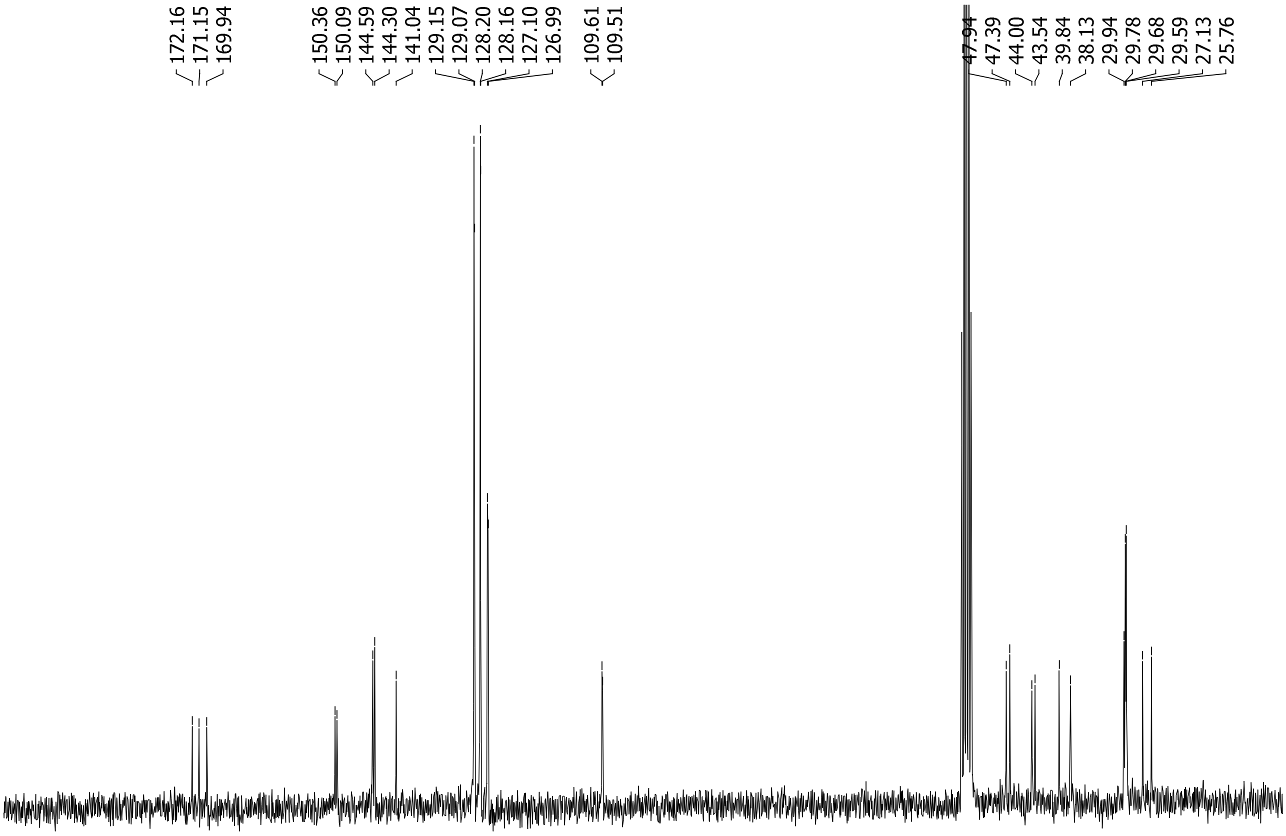

6 (^{1}H NMR, 300 MHz, $CDCl_3$, 298 K)

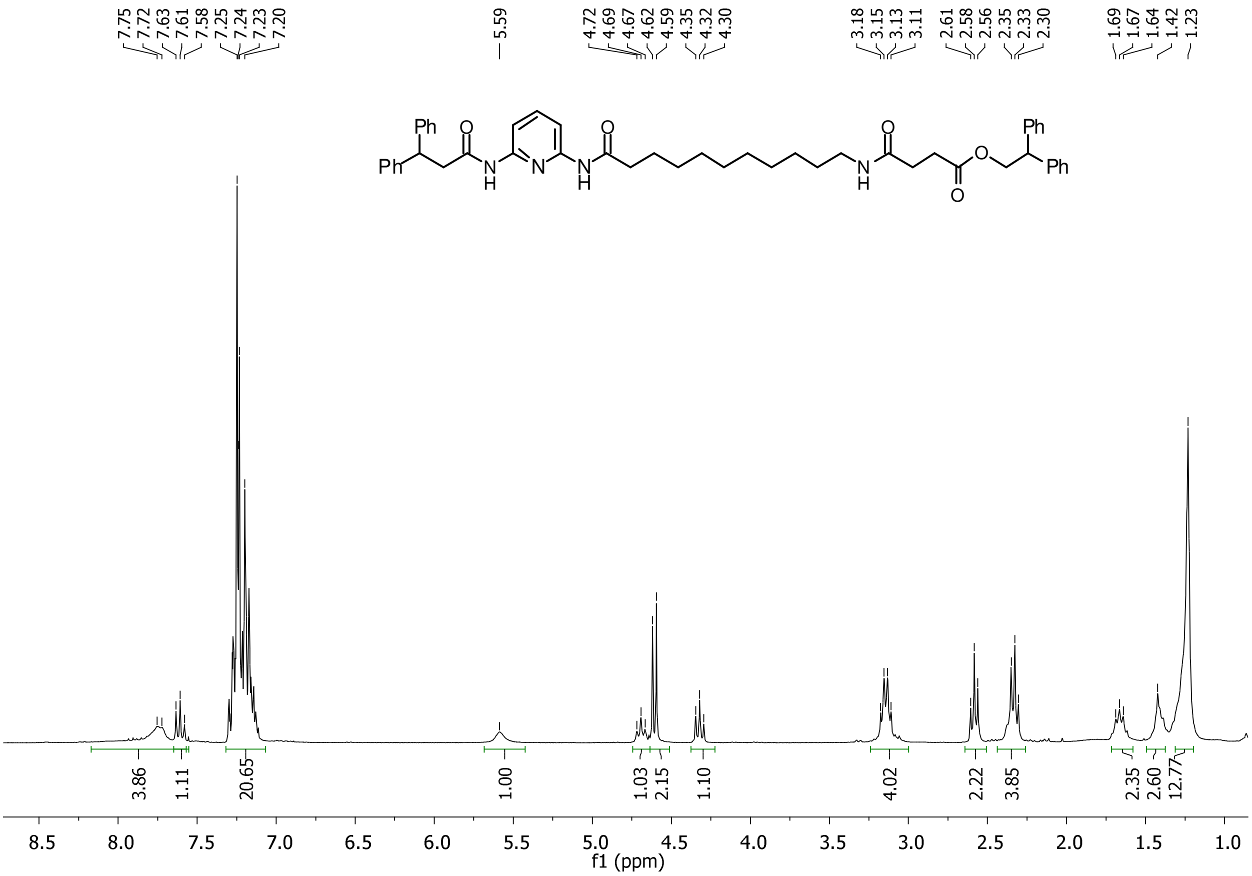

6 (^{13}C NMR, 75 MHz, $CDCl_3$, 298 K)

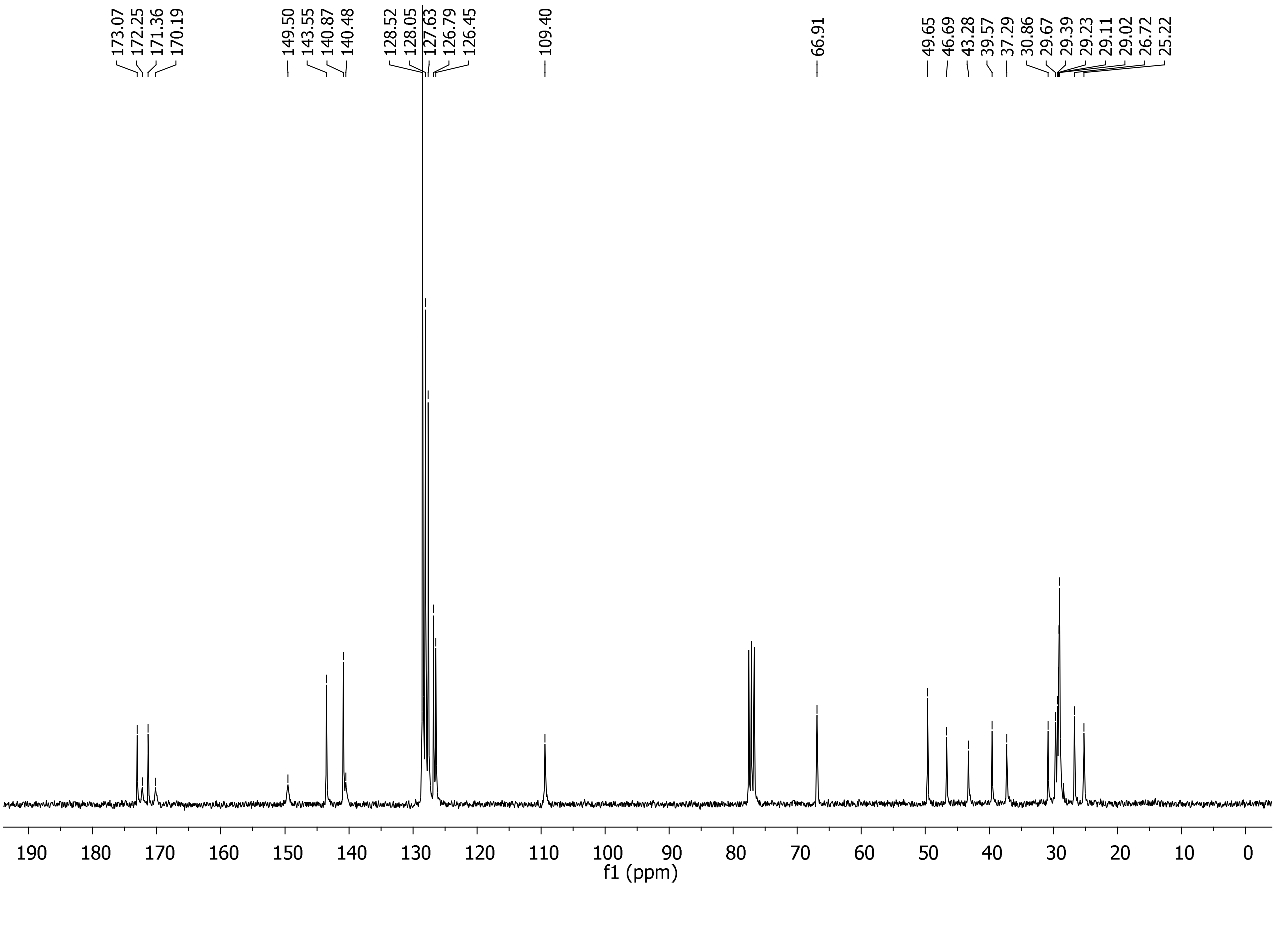

7 (^{1}H NMR, 400 MHz, CD_2Cl_2, 298 K)

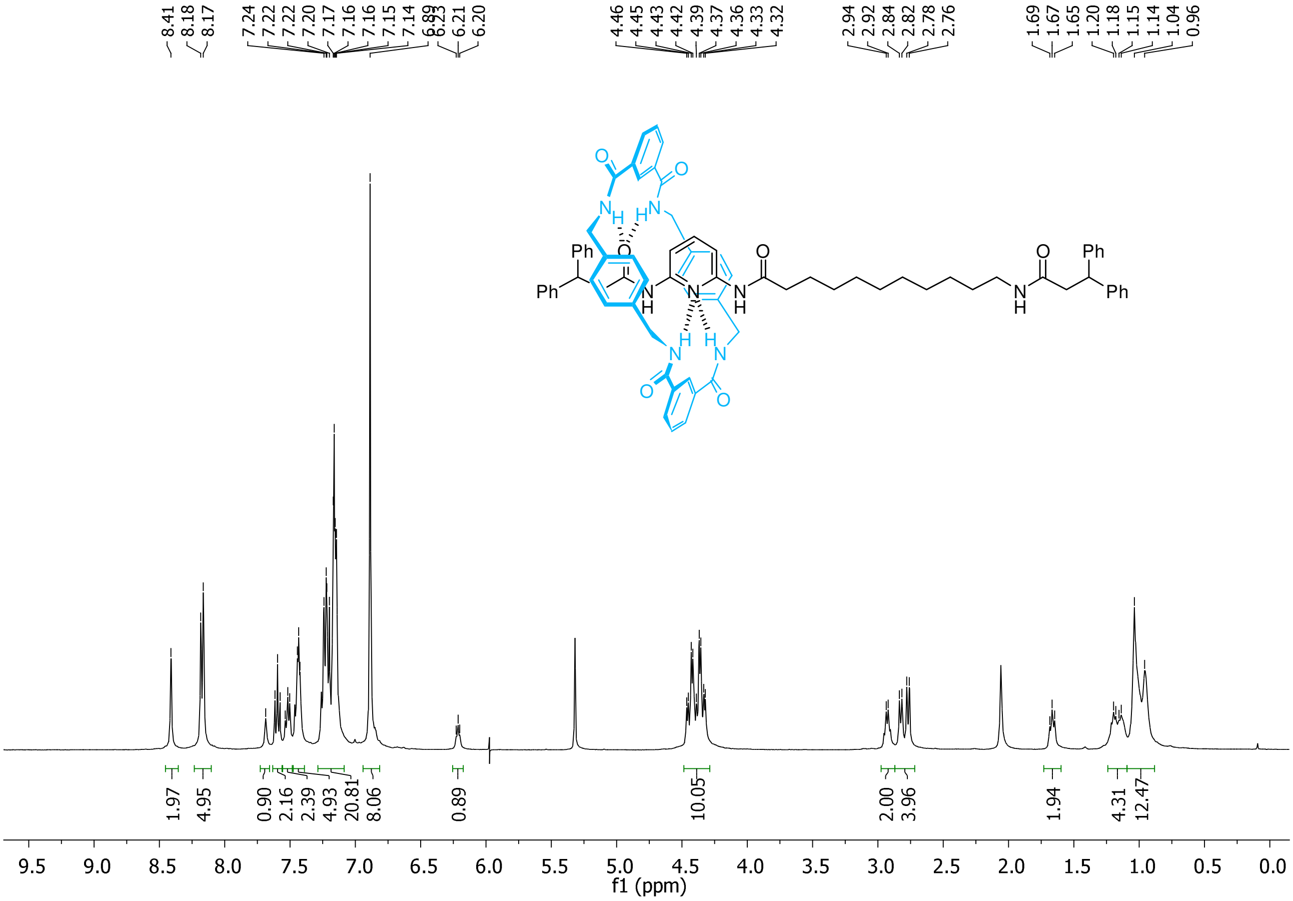

7 (^{13}C NMR, 100 MHz, CD_2Cl_2, 298 K)

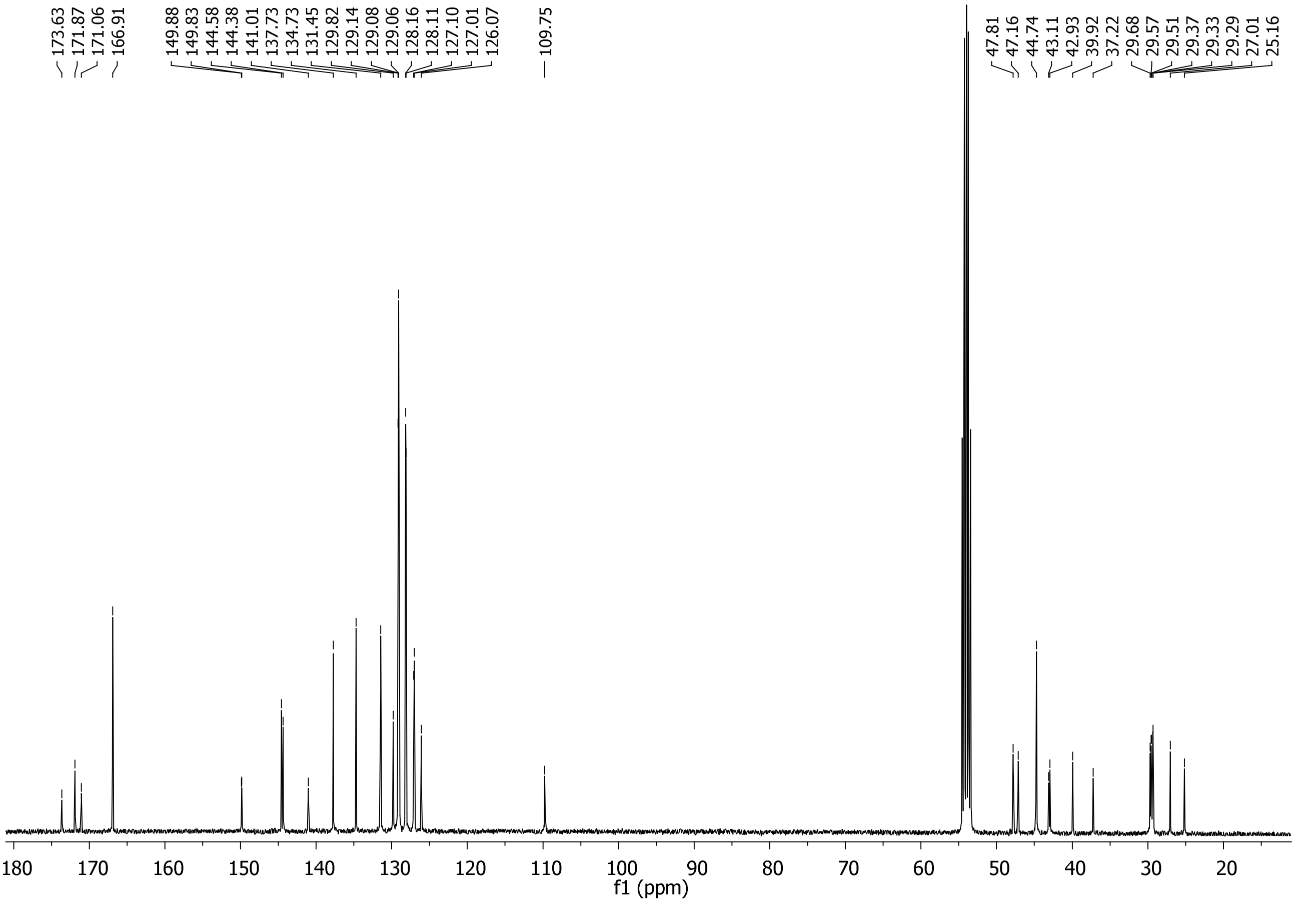

8 (^{1}H NMR, 300 MHz, CD_2Cl_2, 298 K)

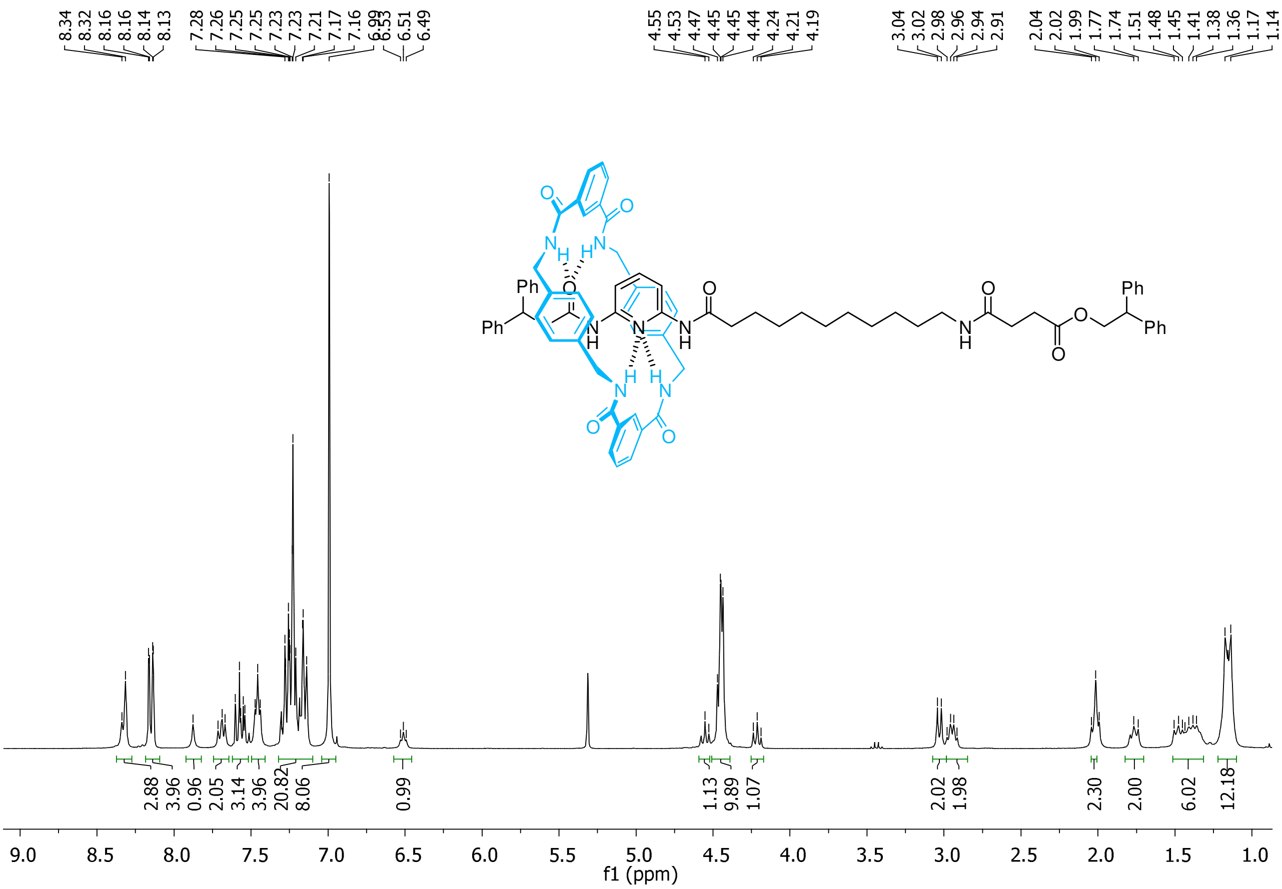

8 (^{13}C NMR, 75 MHz, CD_2Cl_2, 298 K)

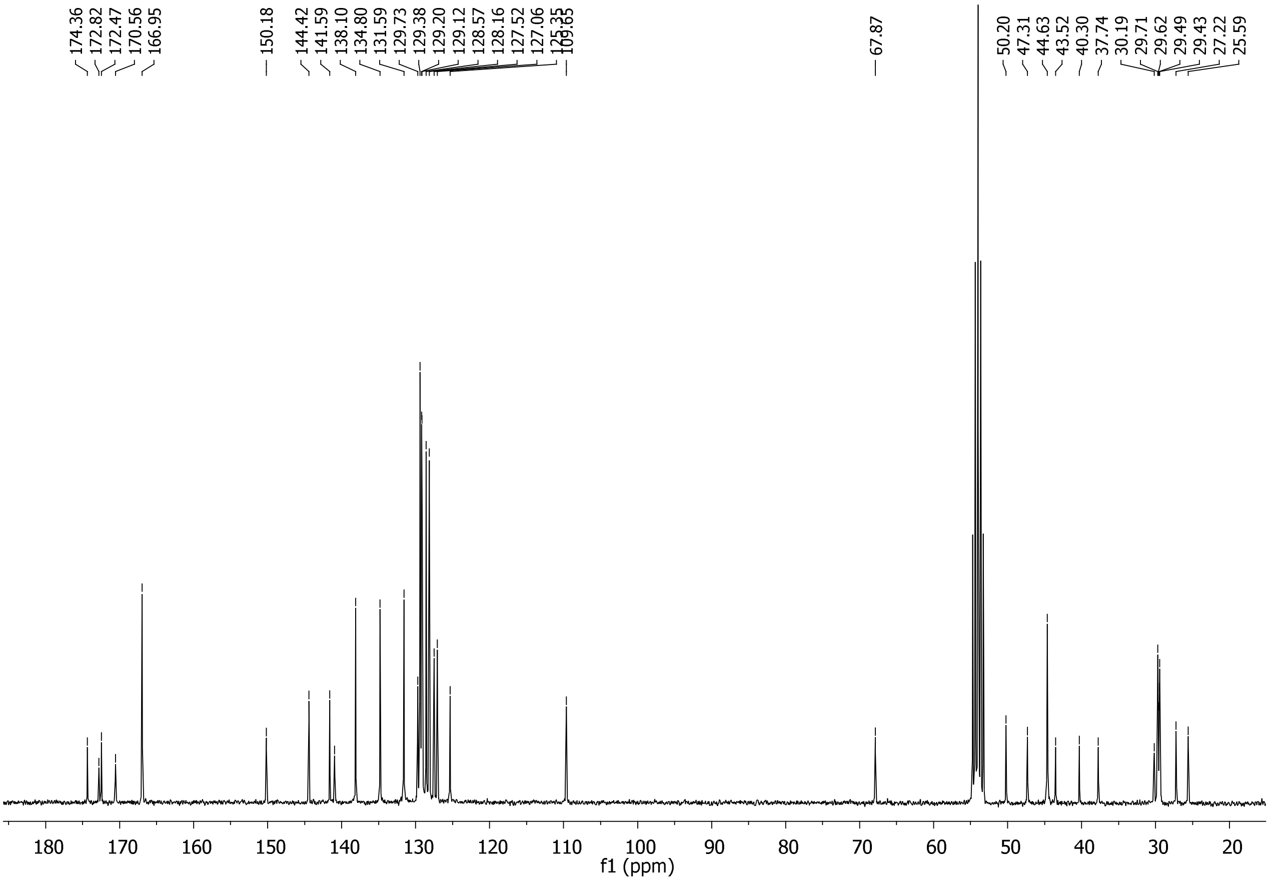

1b (^{1}H NMR, 400 MHz, CD_2Cl_2, 298 K)

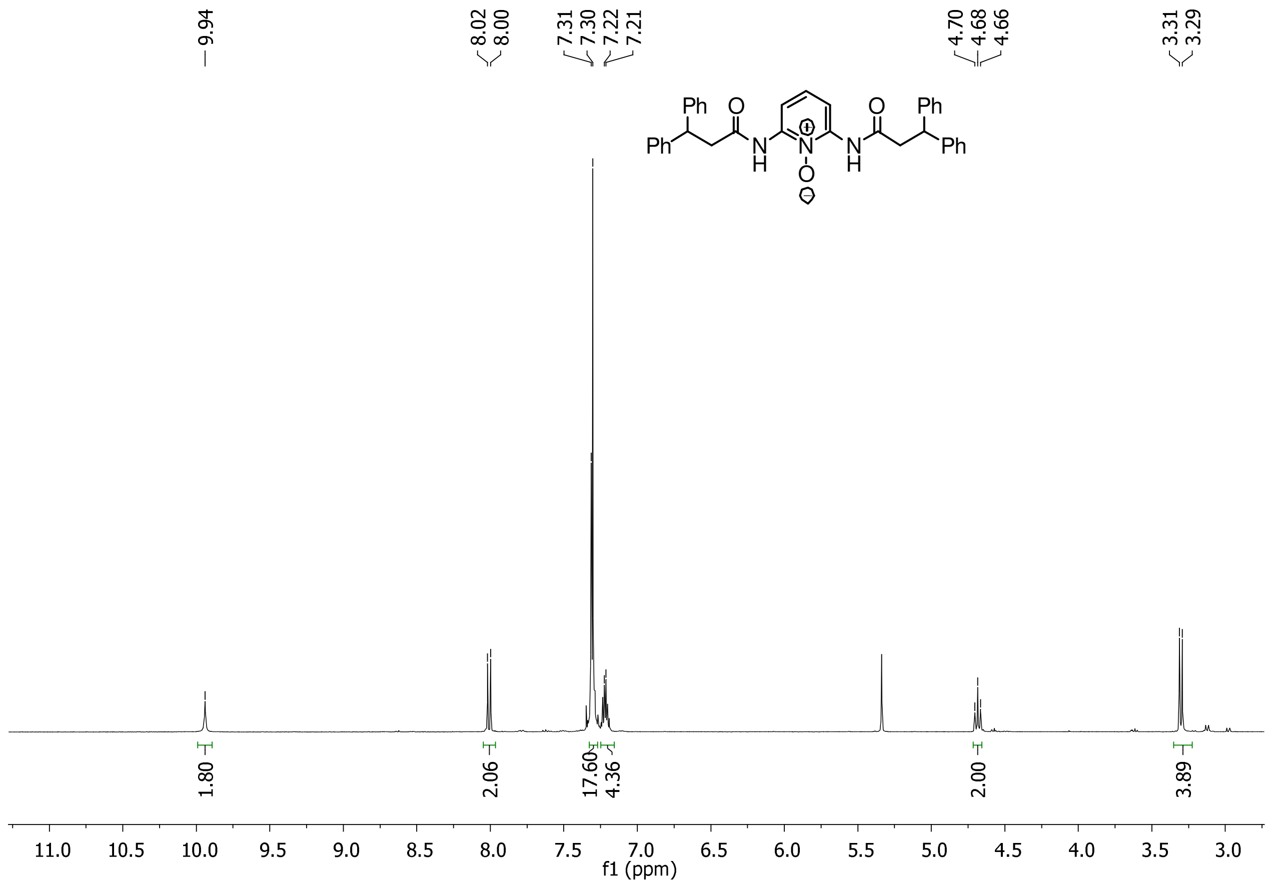

1b (^{13}C NMR, 100 MHz, CD_2Cl_2, 298 K)

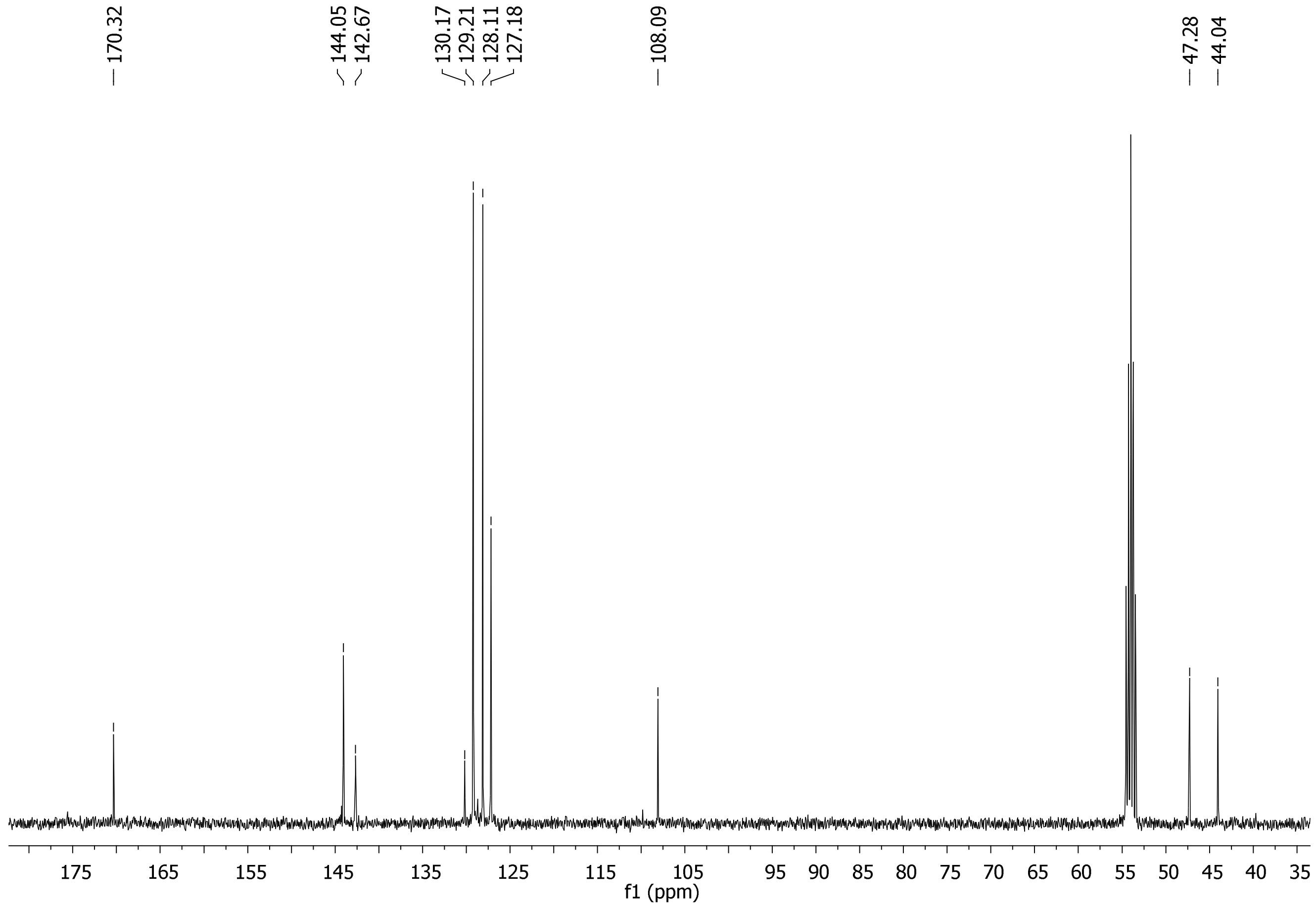

2b (^{1}H NMR, 400 MHz, $C_2D_2Cl_4$, **373** K)

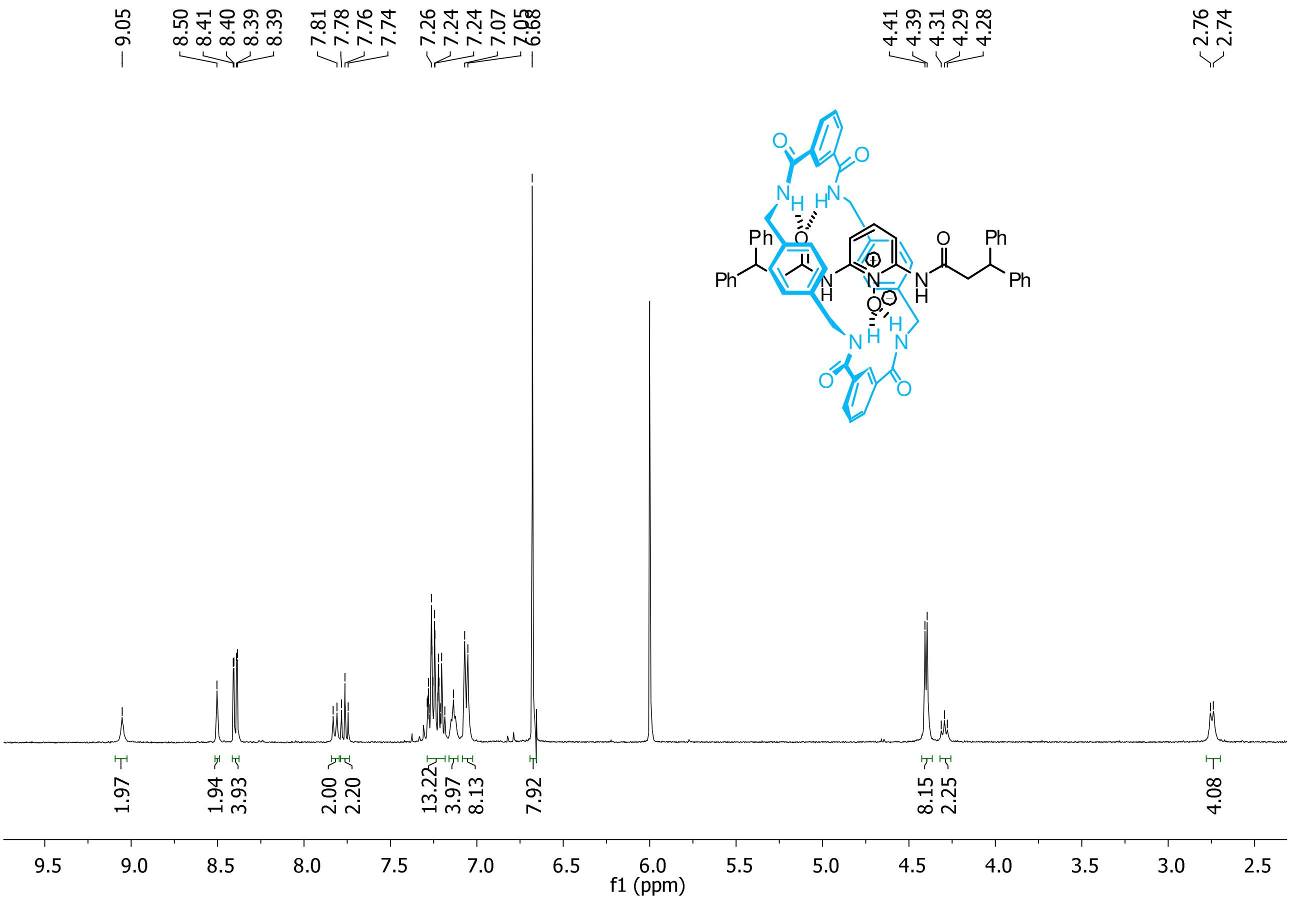

2b (^{13}C NMR, 100 MHz, $C_2D_2Cl_4$, **373** K)

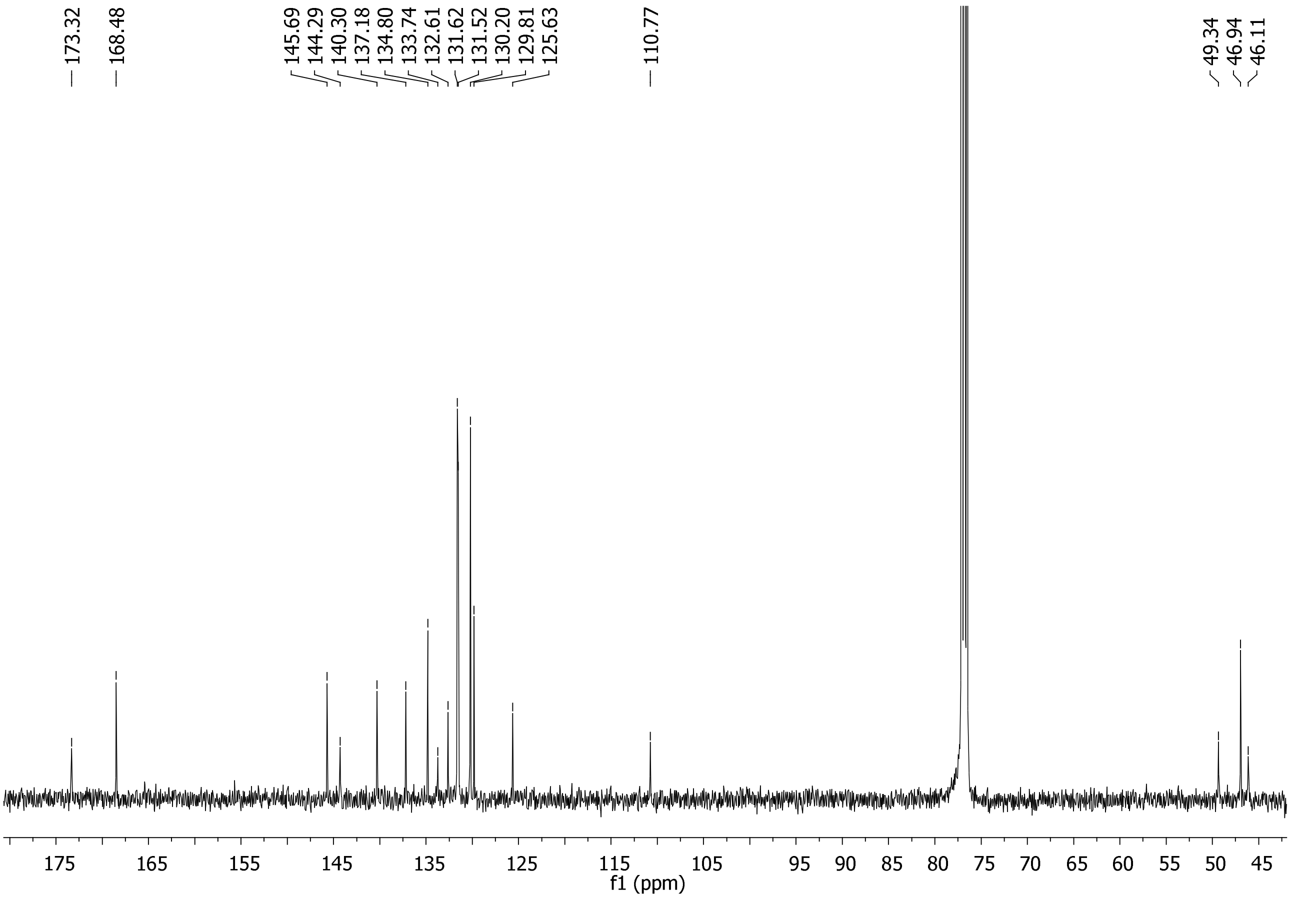

13 (^{1}H NMR, 400 MHz, $CDCl_3$, 298 K)

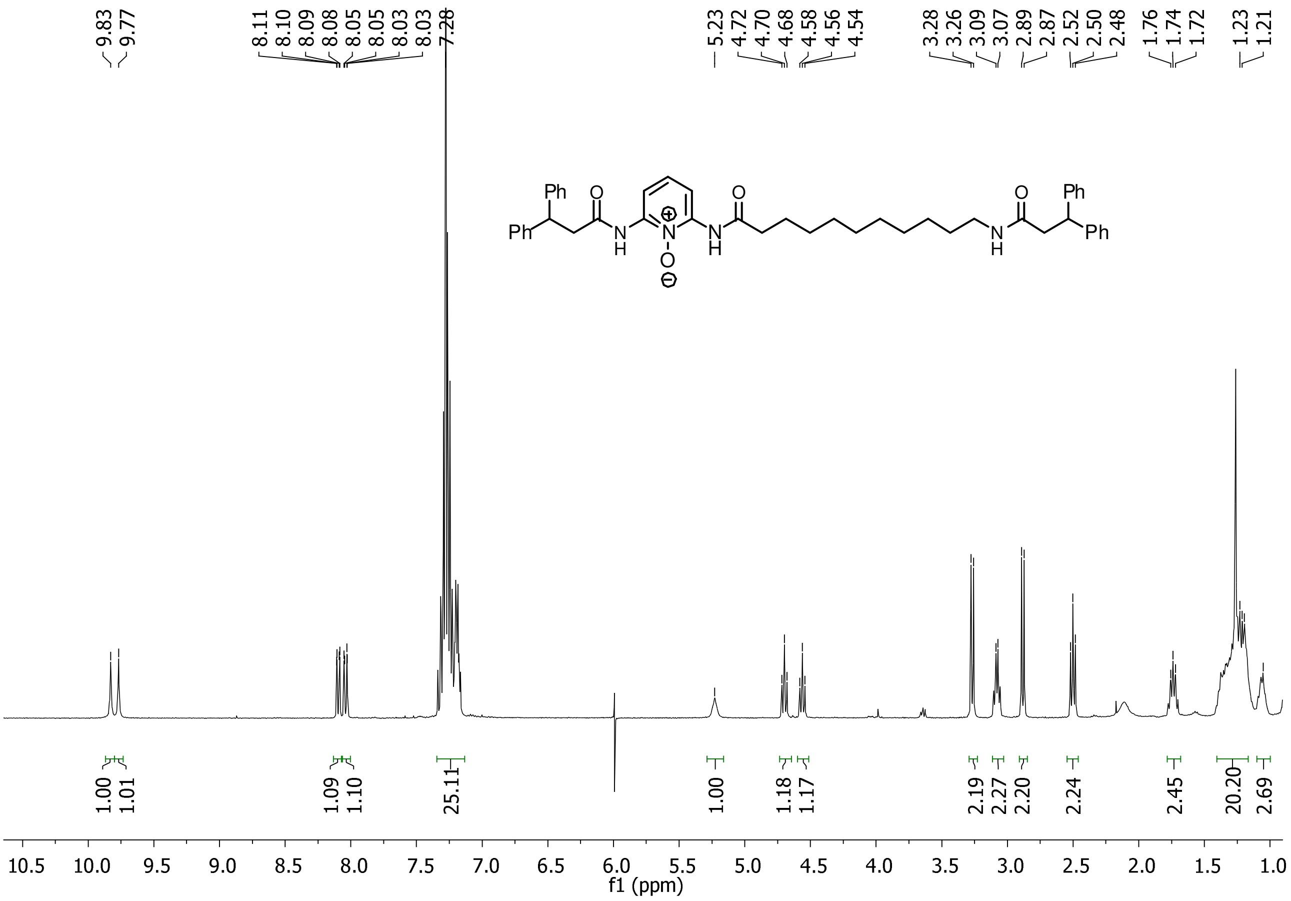

13 (^{13}C NMR, 100 MHz, $CDCl_3$, 298 K)

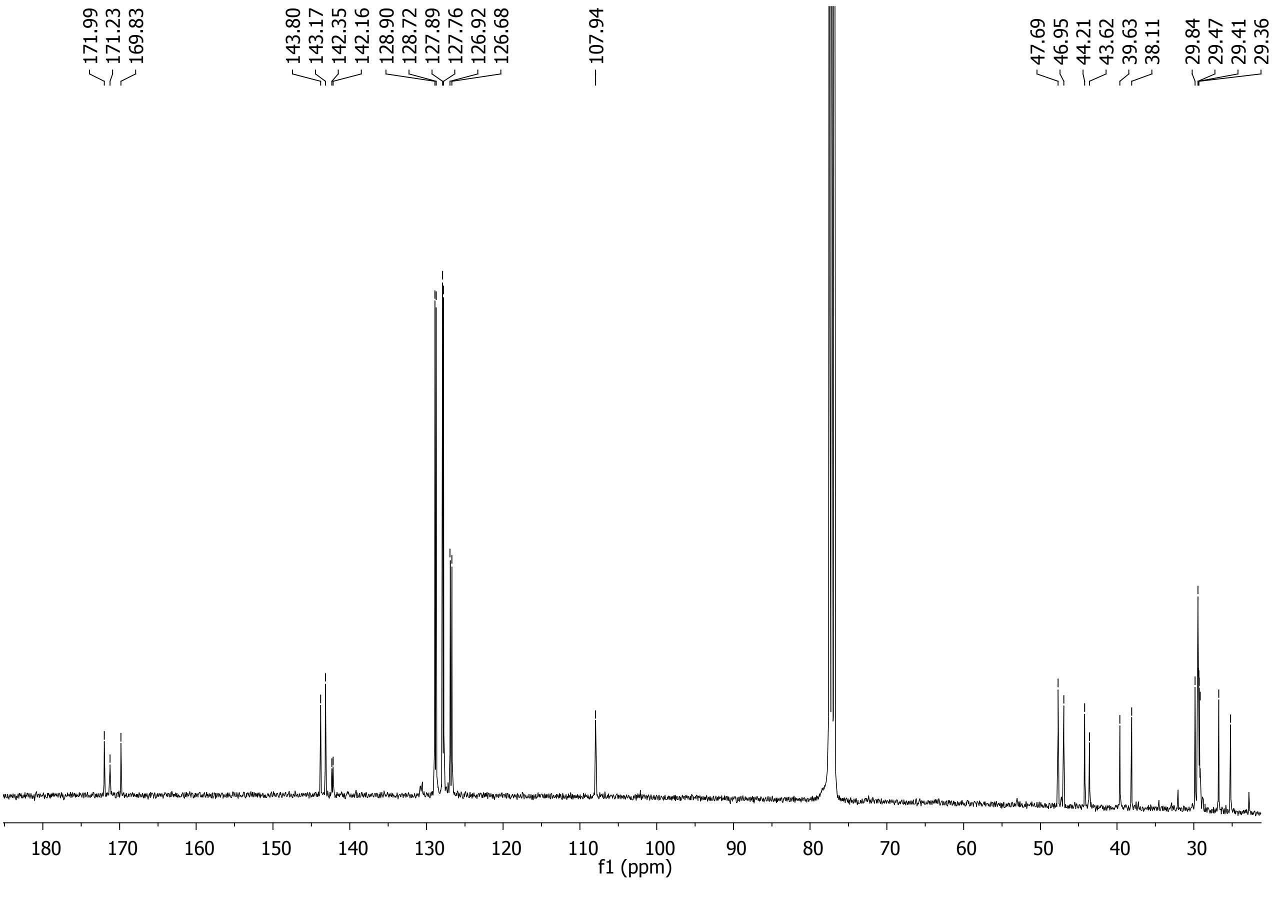

12 (^{1}H NMR, 400 MHz, $C_2D_2Cl_4$, **373** K)

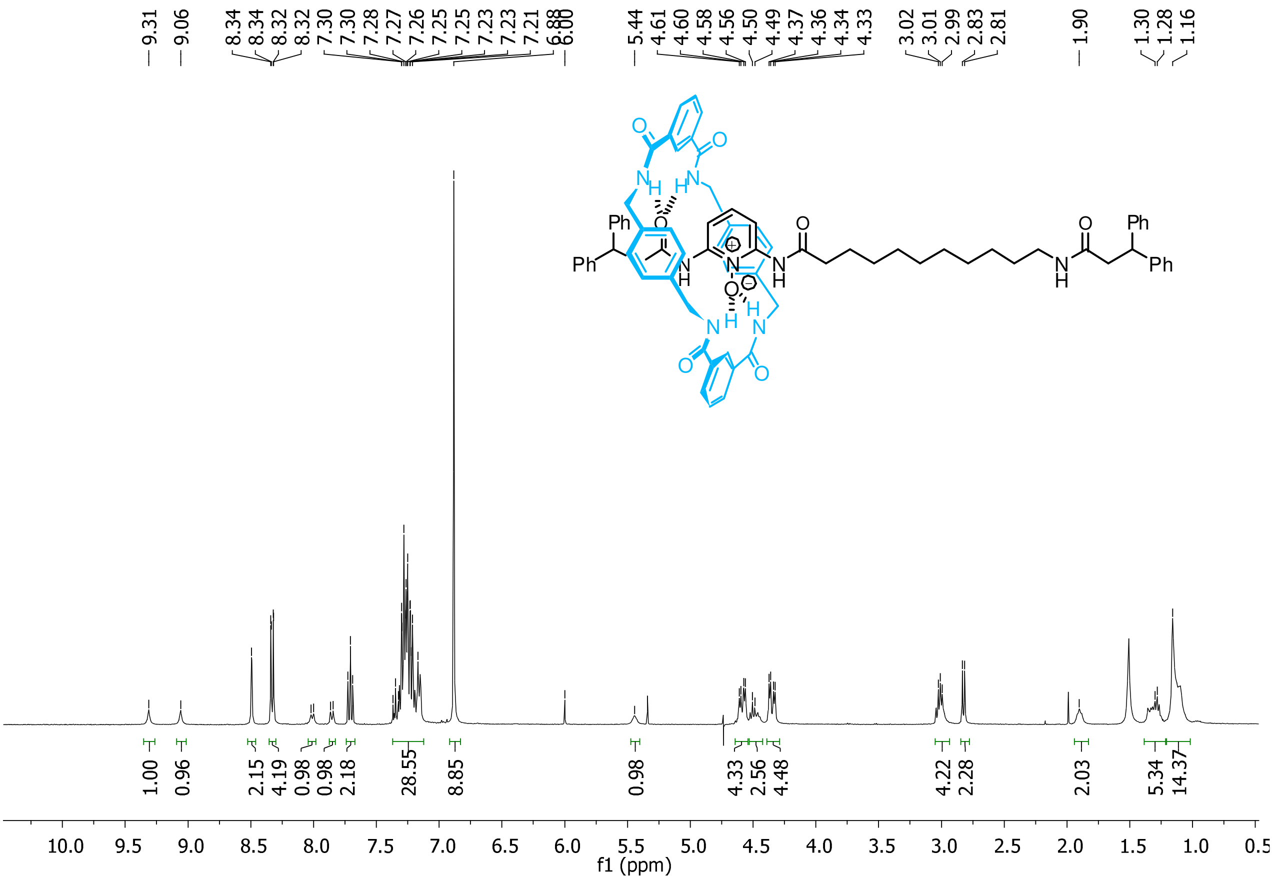

12 (^{13}C NMR, 100 MHz, $C_2D_2Cl_4$, **373** K)

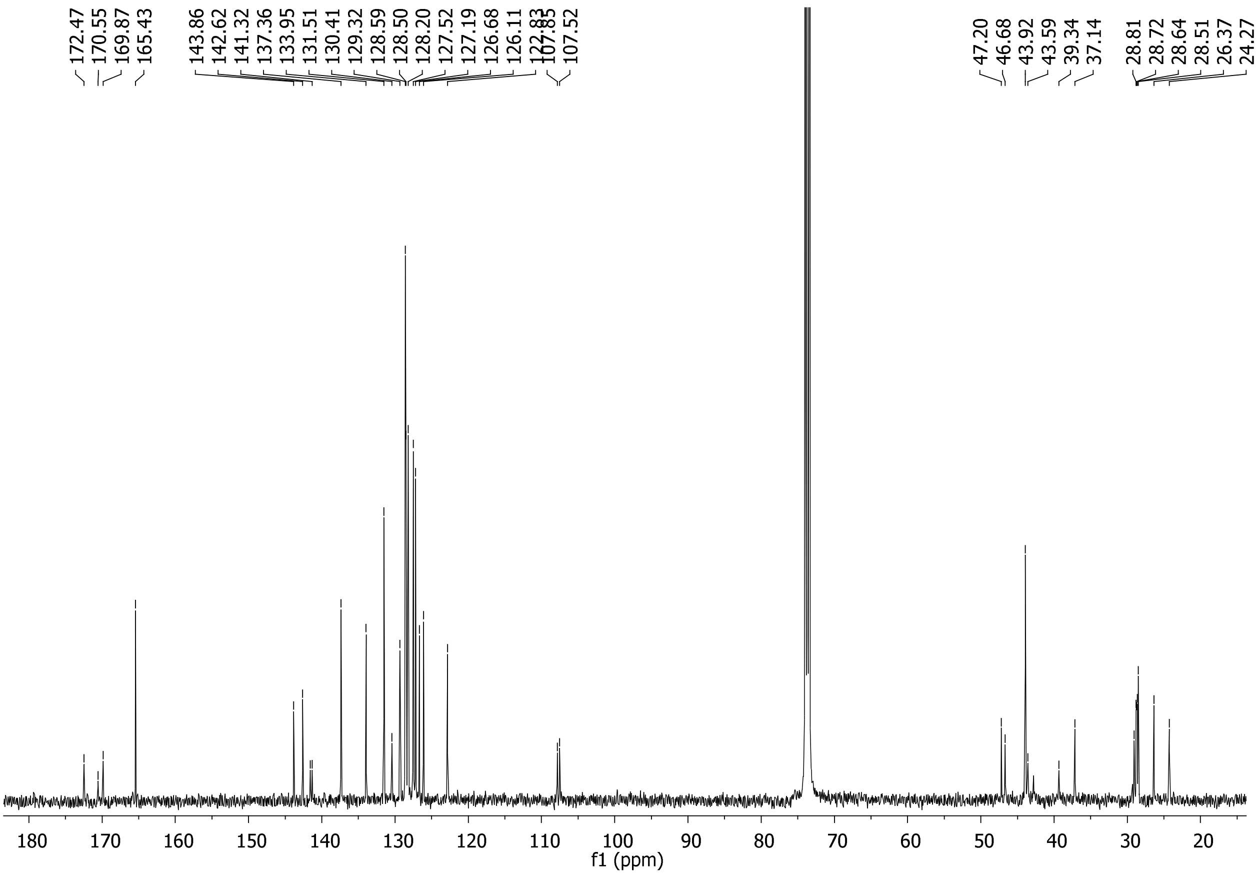

1c (^{1}H NMR, 300 MHz, CD_2Cl_2, 298 K)

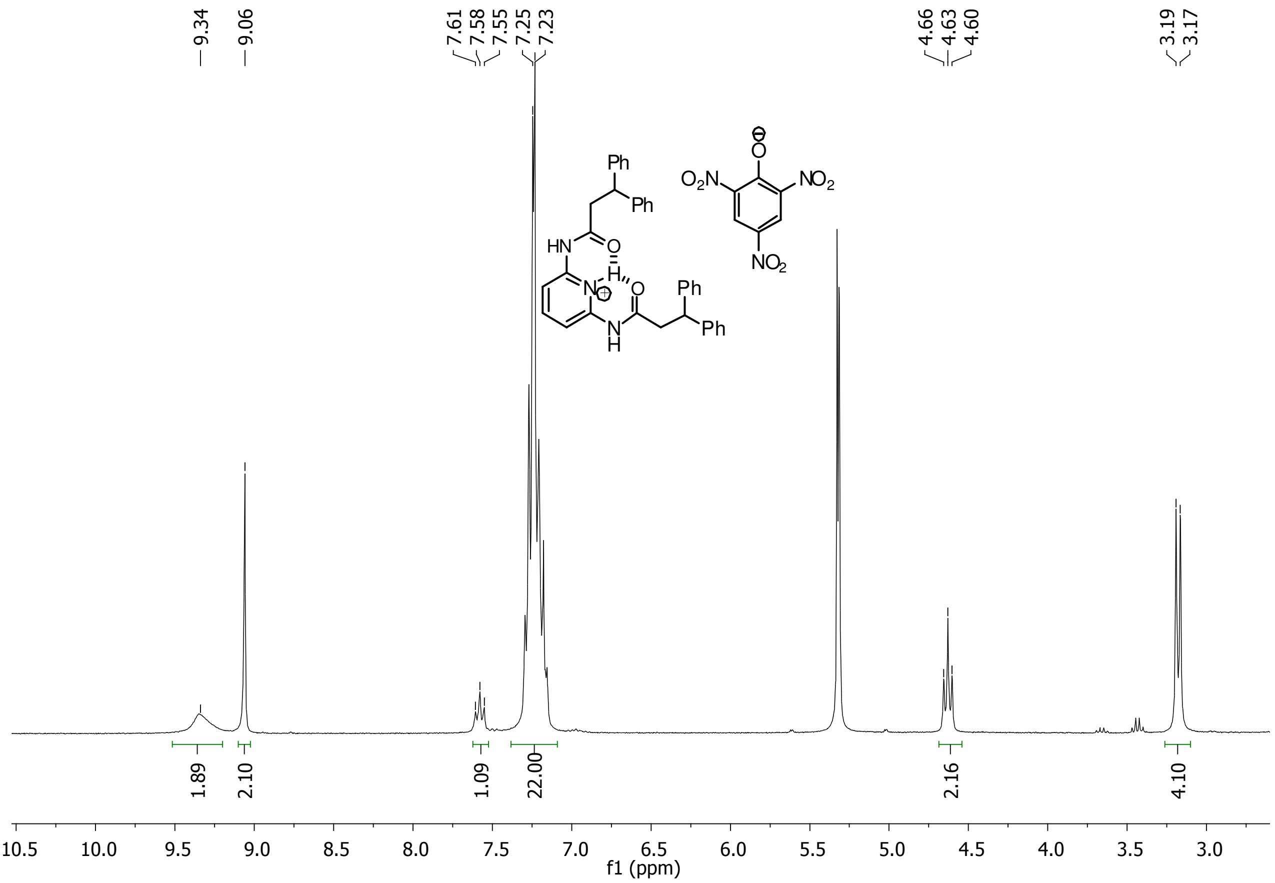

1c (^{13}C NMR, 75 MHz, CD_2Cl_2, 298 K)

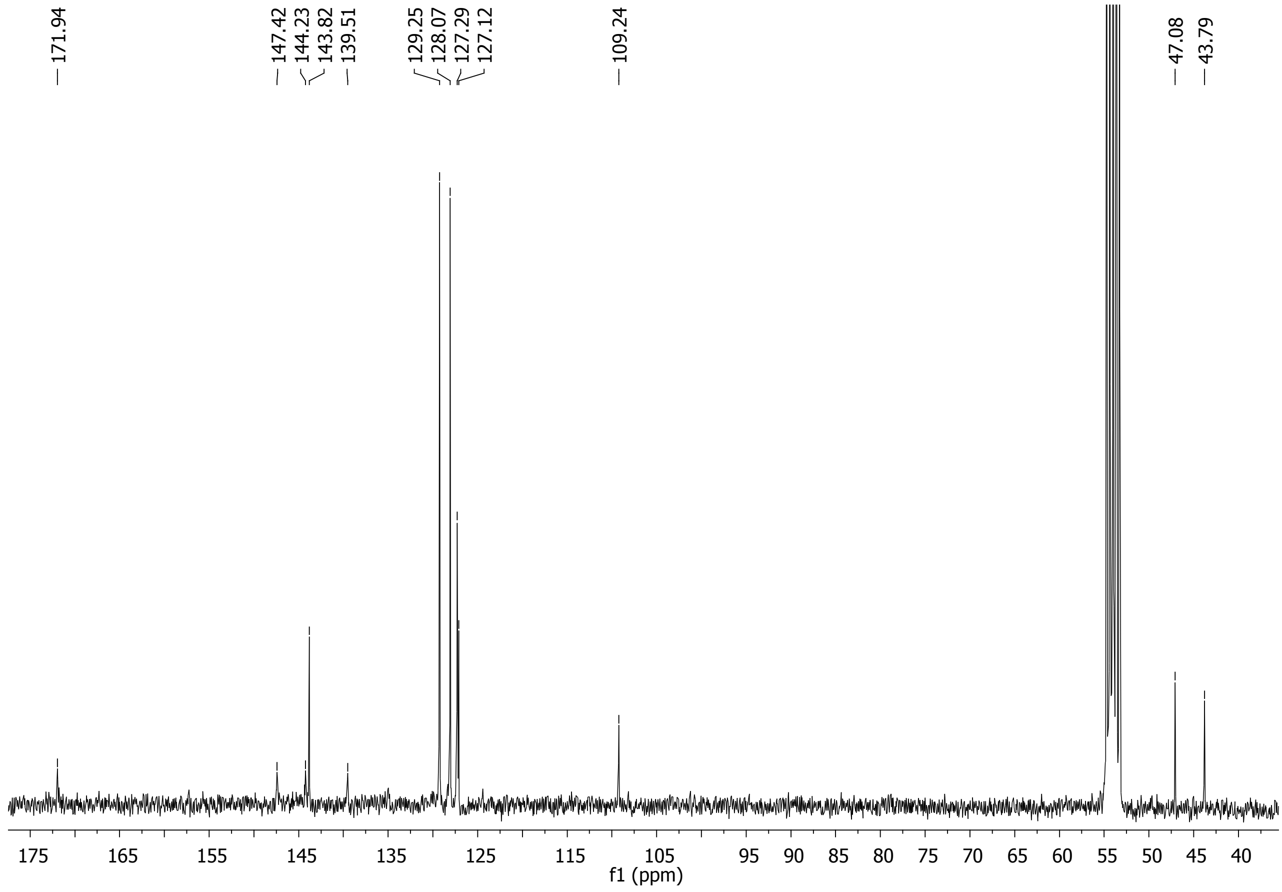

2c (^{1}H NMR, 400 MHz, CD_2Cl_2, 298 K)

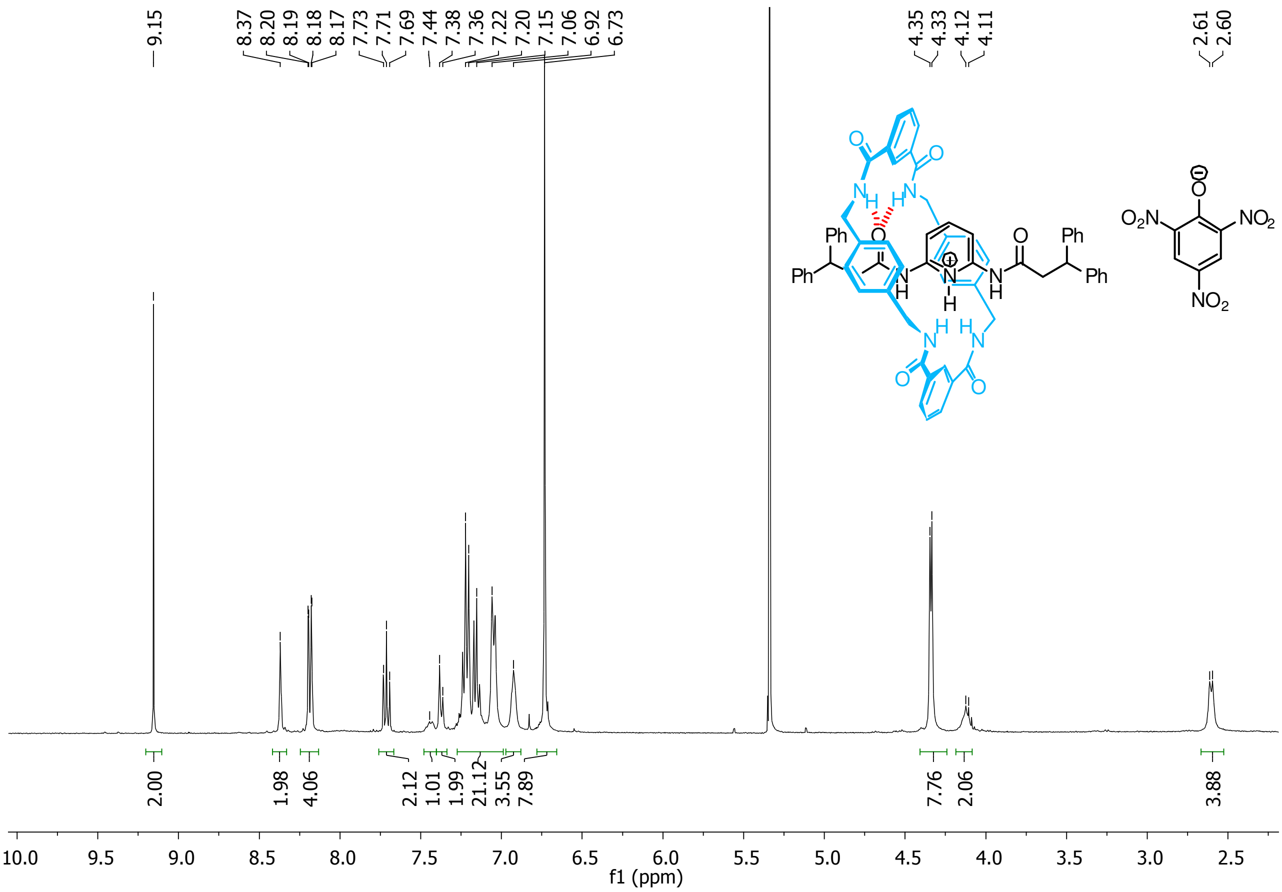

2c (^{13}C NMR, 100 MHz, CD_2Cl_2, 298 K)

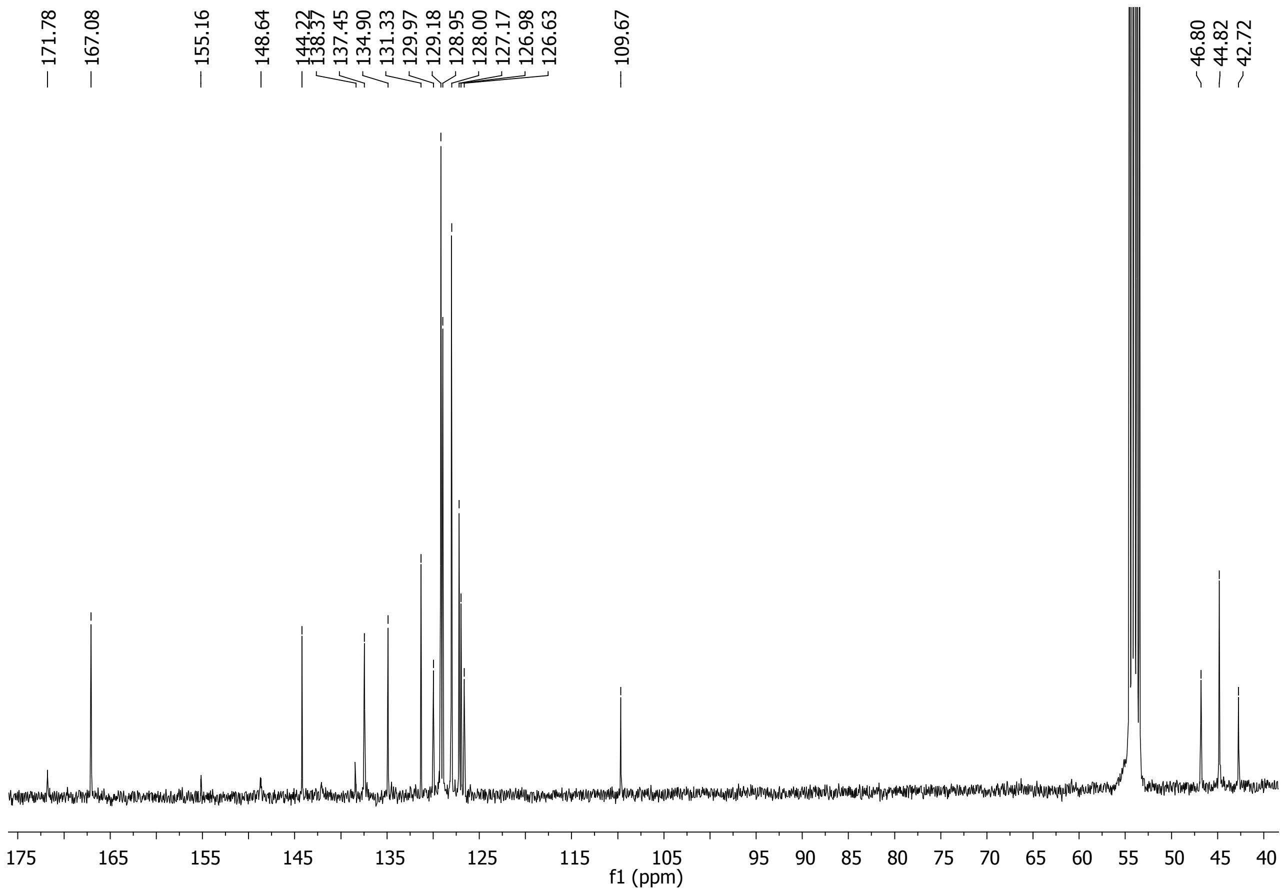

15 (^{1}H NMR, 400 MHz, CD_2Cl_2, 298 K)

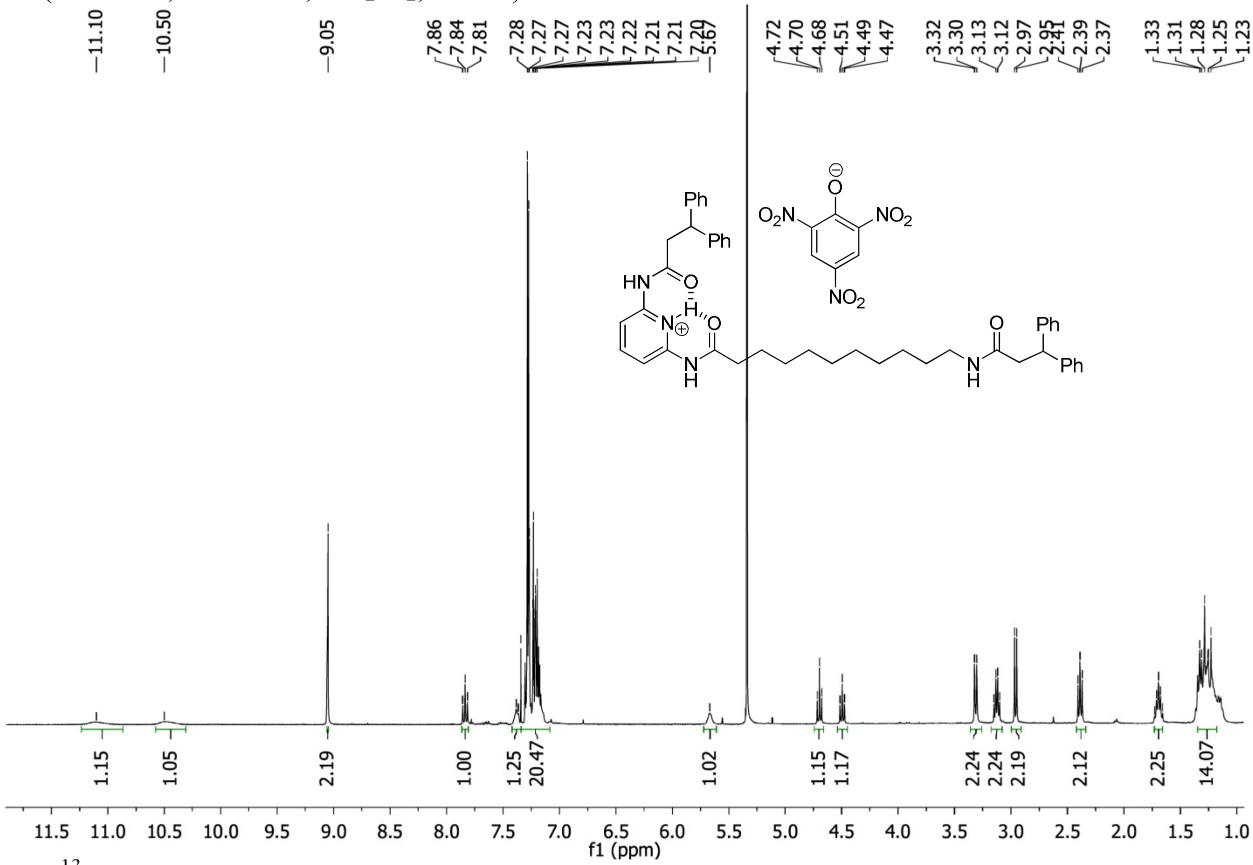

15 (^{13}C NMR, 100 MHz, CD_2Cl_2, 298 K)

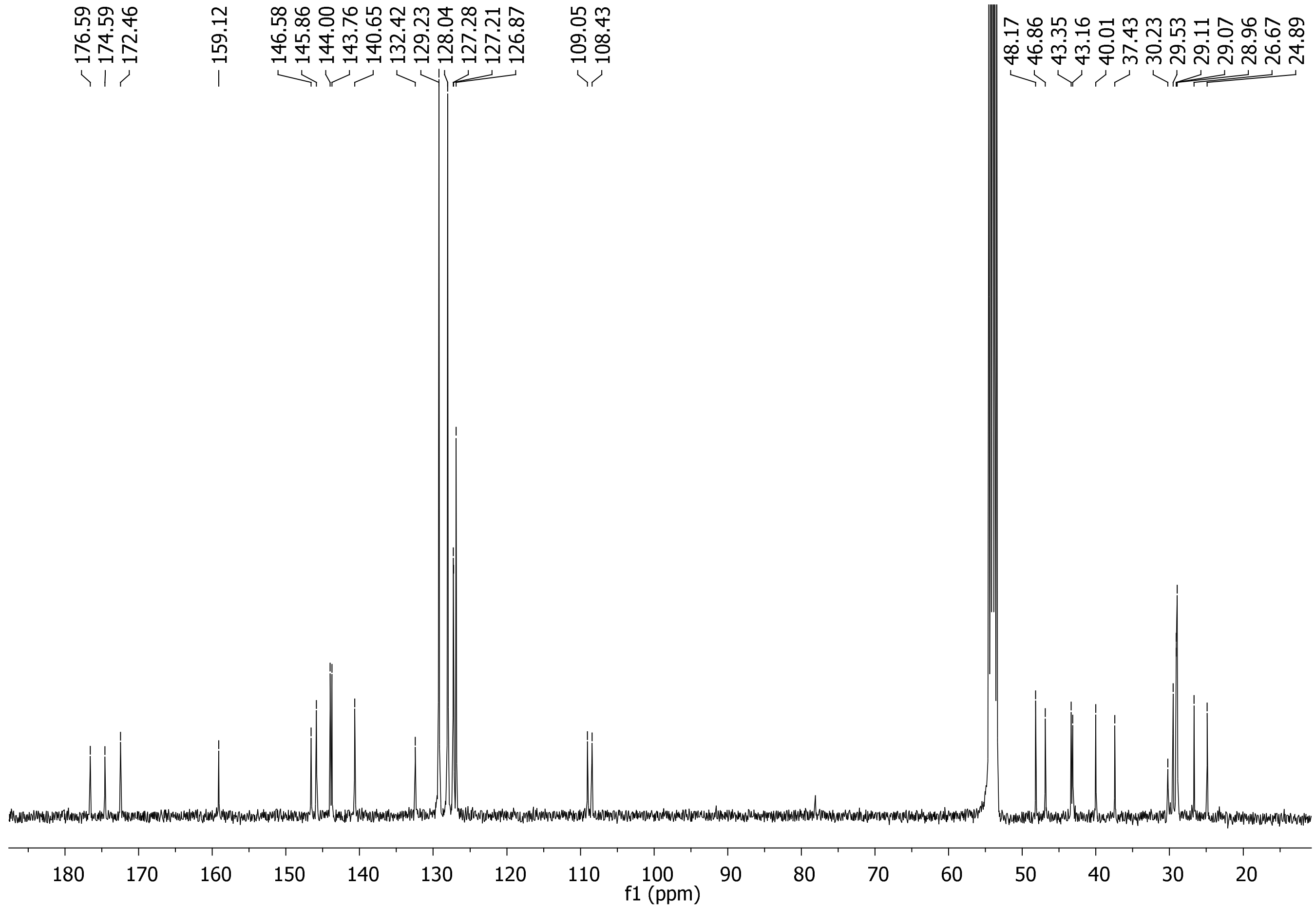

14 (^{1}H NMR, 300 MHz, CD_2Cl_2, 298 K)

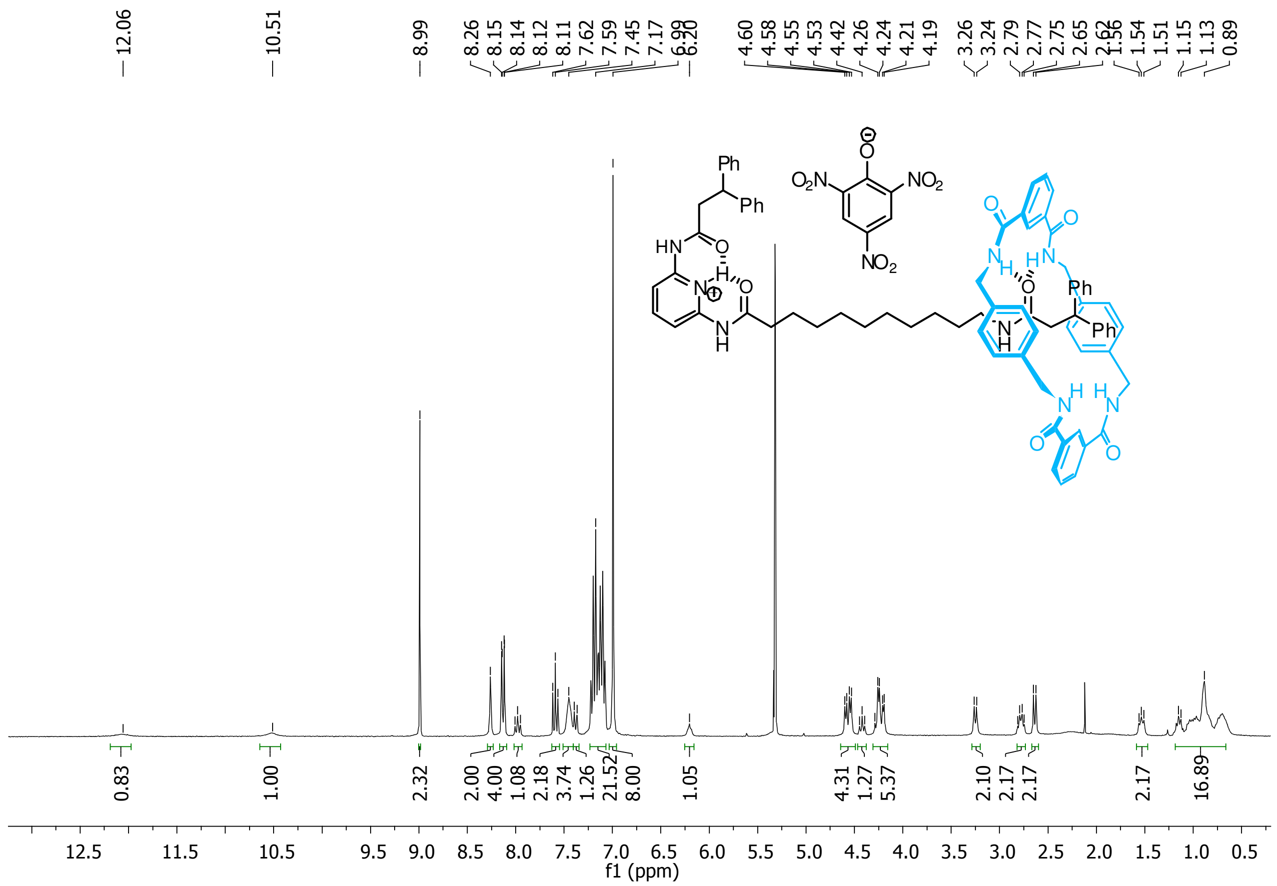

14 (^{1}H NMR, 75 MHz, CD_2Cl_2, 298 K)

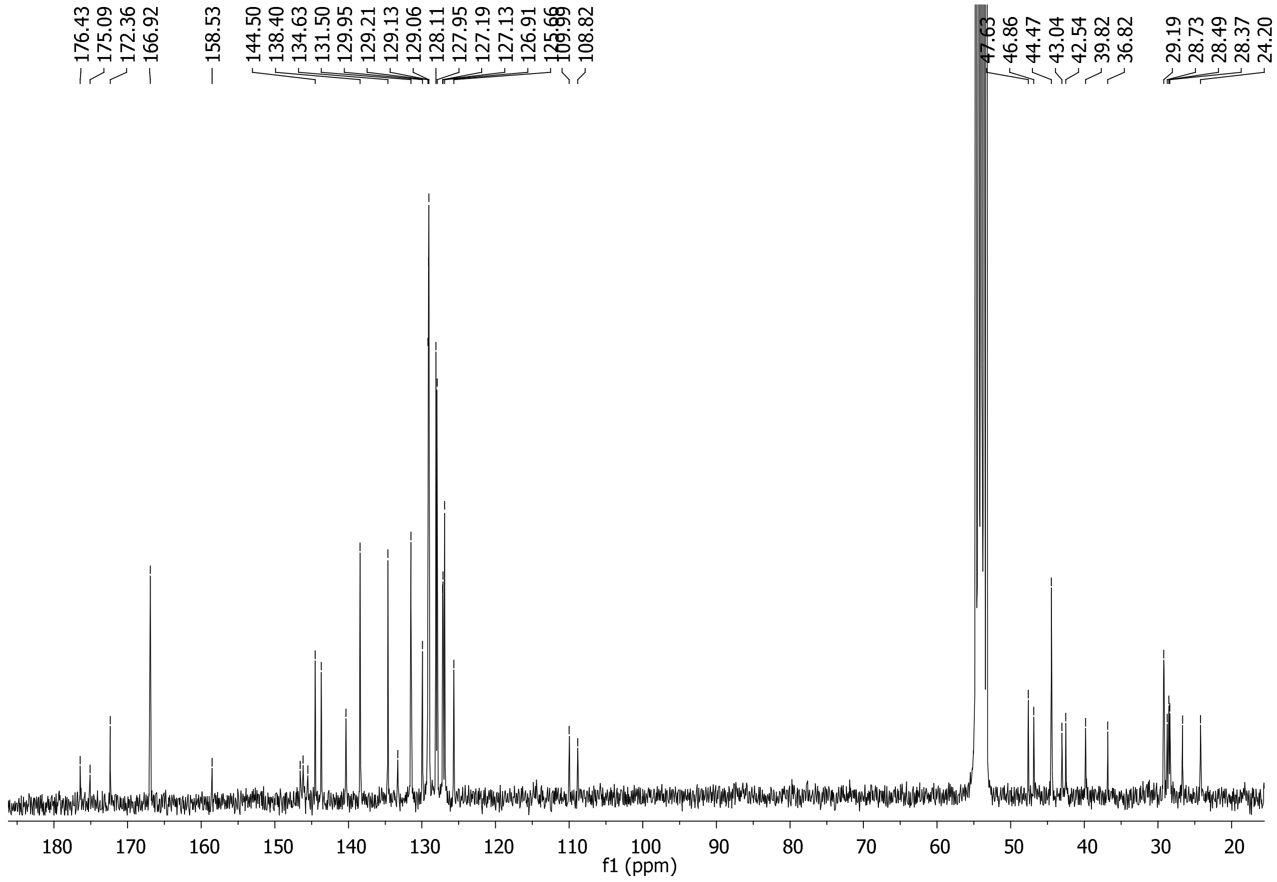

Supplement: Supplementary file 1 [file SC-006-C5SC00790A-s001.pdf]
